# A Bioconductor workflow for processing, evaluating, and interpreting expression proteomics data

**DOI:** 10.12688/f1000research.139116.1

**Published:** 2023-10-24

**Authors:** Charlotte Hutchings, Charlotte S. Dawson, Thomas Krueger, Kathryn S. Lilley, Lisa M. Breckels

**Affiliations:** 1 Cambridge Centre for Proteomics, University of Cambridge, Cambridge, CB2 1QR, UK; 2 Department of Biochemistry, University of Cambridge, Cambridge, CB2 1QR, UK

**Keywords:** Bioconductor, QFeatures, proteomics, shotgun proteomics, bottom-up proteomics, differential expression, mass spectrometry, quality control, data processing, limma

## Abstract

**Background:** Expression proteomics involves the global evaluation of protein abundances within a system. In turn, differential expression analysis can be used to investigate changes in protein abundance upon perturbation to such a system.

**Methods:** Here, we provide a workflow for the processing, analysis and interpretation of quantitative mass spectrometry-based expression proteomics data. This workflow utilizes open-source R software packages from the Bioconductor project and guides users end-to-end and step-by-step through every stage of the analyses. As a use-case we generated expression proteomics data from HEK293 cells with and without a treatment. Of note, the experiment included cellular proteins labelled using tandem mass tag (TMT) technology and secreted proteins quantified using label-free quantitation (LFQ).

**Results:** The workflow explains the software infrastructure before focusing on data import, pre-processing and quality control. This is done individually for TMT and LFQ datasets. The application of statistical differential expression analysis is demonstrated, followed by interpretation via gene ontology enrichment analysis.

**Conclusions:** A comprehensive workflow for the processing, analysis and interpretation of expression proteomics is presented. The workflow is a valuable resource for the proteomics community and specifically beginners who are at least familiar with R who wish to understand and make data-driven decisions with regards to their analyses.

## Introduction

Proteins are responsible for carrying out a multitude of biological tasks, implementing cellular functionality and determining phenotype. Mass spectrometry (MS)-based expression proteomics allows protein abundance to be quantified and compared between samples. In turn, differential protein abundance can be used to explore how biological systems respond to a perturbation. Many research groups have applied such methodologies to understand mechanisms of disease, elucidate cellular responses to external stimuli, and discover diagnostic biomarkers (see Refs. 1– 3 for recent examples). As the potential of proteomics continues to be realised, there is a clear need for resources demonstrating how to deal with expression proteomics data in a robust and standardised manner.

The data generated during an expression proteomics experiment are complex, and unfortunately there is no one-size-fits-all method for the processing and analysis of such data. The reason for this is two-fold. Firstly, there are a wide range of experimental methods that can be used to generate expression proteomics data. Researchers can analyse full-length proteins (top-down proteomics) or complete an enzymatic digestion and analyse the resulting peptides. This proteolytic digestion can be either partial (middle-down proteomics) or complete (bottom-up proteomics). The latter approach is most commonly used as peptides have a more favourable ionisation capacity, predictable fragmentation patterns, and can be separated via reversed phase liquid chromatography, ultimately making them more compatible with MS. ^4^ Within bottom-up proteomics, the relative quantitation of peptides can be determined using one of two approaches: (1) label-free or (2) label-based quantitation. Moreover, the latter can be implemented with a number of different peptide labelling chemistries, for example, using tandem mass tag (TMT), stable-isotope labelling by amino acids in cell culture (SILAC), isobaric tags for relative and absolute quantitation (iTRAQ), among others. ^5^ MS analysis can also be used in either data-dependent or data-independent acquisition (DDA or DIA) mode. ^6^^,^^7^ Although all of these experimental methods typically result in a similar output, a matrix of quantitative values, the data are different and must be treated as such. Secondly, data processing is dependent upon the experimental goal and biological question being asked.

Here, we provide a step-by-step workflow for processing, analysing and interpreting expression proteomics data derived from a bottom-up experiment using DDA and either LFQ or TMT label-based peptide quantitation. We outline how to process the data starting from a peptide spectrum match (PSM)- or peptide- level .txt file. Such files are the outputs of most major third party search software (e.g. Proteome Discoverer, MaxQuant, FragPipe). We begin with data import and then guide users through the stages of data processing including data cleaning, quality control filtering, management of missing values, imputation, and aggregation to protein-level. Finally, we finish with how to discover differentially abundant proteins and carry out biological interpretation of the resulting data. The latter will be achieved through the application of gene ontology (GO) enrichment analysis. Hence, users can expect to generate lists of proteins that are significantly up- or downregulated in their system of interest, as well as the GO terms that are significantly over-represented in these proteins.

Using the R statistical programming environment ^8^ we make use of several state-of-the-art packages from the open-source, open-development Bioconductor project ^9^ to analyse use-case expression proteomics datasets ^10^ from both LFQ and label-based technologies.

### Package installation

In this workflow we make use of open-source software from the R Bioconductor ^9^ project. The Bioconductor initiative provides R software packages dedicated to the processing of high-throughput complex biological data. Packages are open-source, well-documented and benefit from an active community of developers. We recommend users to download the RStudio integrated development environment (IDE) which provides a graphical interface to R programming language.

Detailed instructions for the installation of Bioconductor packages are documented on the Bioconductor Installation page. The main packages required for this workflow are installed using the code below.

if (!require("BiocManager", quietly = TRUE)) {
install.packages("BiocManager")
}


BiocManager::install(c("QFeatures",
                       "ggplot2",
                       "stringr"
                       "NormalyzerDE",
                       "corrplot",
                       "Biostrings",
                       "limma",
                       "impute",
                       "dplyr",
                       "tibble",
                       "org.Hs.eg.db",
                       "clusterProfiler",
                       "enrichplot"))


After installation, each package must be loaded before it can be used in the R session. This is achieved via the library function. For example, to load the QFeatures package one would type library("QFeatures") after installation. Here we load all packages included in this workflow.

library("QFeatures")
library("ggplot2")
library("stringr")
library("dplyr")
library("tibble")
library("NormalyzerDE")
library("corrplot")
library("Biostrings")
library("limma")
library("org.Hs.eg.db")
library("clusterProfiler")
library("enrichplot")


## The use-case: exploring changes in protein abundance in HEK293 cells upon perturbation

**Figure 1.**
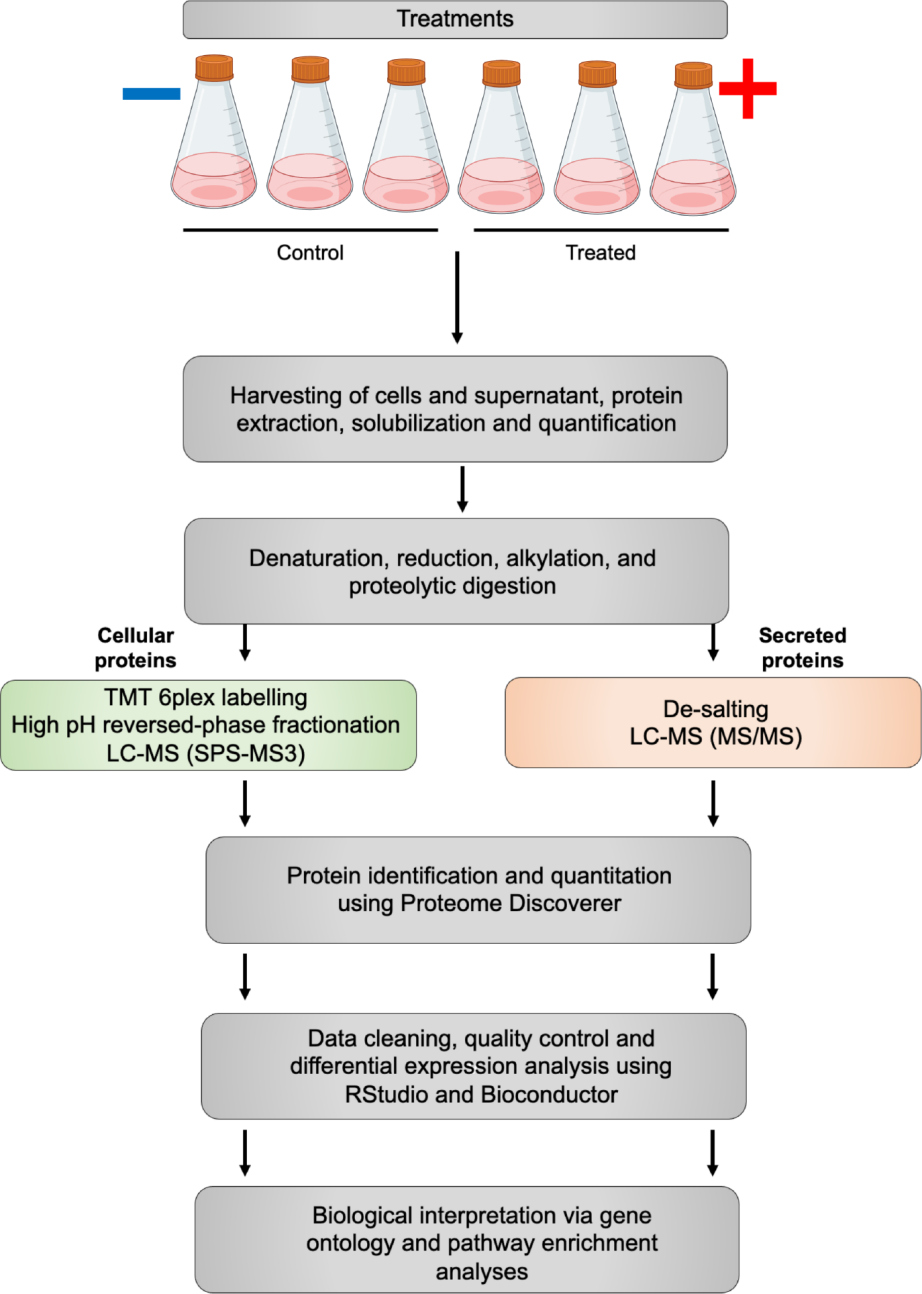
A schematic summary of the experimental protocol used to generate the use-case data.

As a use-case, we analyse two quantitative proteomics datasets derived from a single experiment. The aim of the experiment was to reveal the differential abundance of proteins in HEK293 cells upon a particular treatment, the exact details of which are anonymised for the purpose of this workflow. An outline of the experimental method is provided in Figure 1.

Briefly, HEK293 cells were either (i) left untreated, or (ii) provided with the treatment of interest. These two conditions are referred to as ‘control’ and ‘treated’, respectively. Each condition was evaluated in triplicate. At 96-hours post-treatment, samples were collected and separated into cell pellet and supernatant fractions containing cellular and secreted proteins, respectively. Both fractions were denatured, alkylated and digested to peptides using trypsin.

The supernatant fractions were de-salted and analysed over a two-hour gradient in an Orbitrap Fusion™ Lumos™ Tribrid™ mass spectrometer coupled to an UltiMate™ 3000 HPLC system (Thermo Fisher Scientific). LFQ was achieved at the MS1 level based on signal intensities. Cell pellet fractions were labelled using TMT technology before being pooled and subjected to high pH reversed-phase peptide fractionation giving a total of 8 fractions. As before, each fraction was analysed over a two-hour gradient in an Orbitrap Fusion™ Lumos™ Tribrid™ mass spectrometer coupled to an UltiMate™ 3000 HPLC system (Thermo Fisher Scientific). To improve the accuracy of the quantitation of TMT-labelled peptides, synchronous precursor selection (SPS)-MS3 data acquisition was employed. ^11^^,^^12^ Of note, TMT labelling of cellular proteins was achieved using a single TMT6plex. Hence, this workflow will not include guidance on multi-batch TMT effects or the use of internal reference scaling. For more information about the use of multiple TMTplexes users are directed to Refs. 13, 14.

The cell pellet and supernatant datasets were handled independently and we take advantage of this to discuss the processing of TMT-labelled and LFQ proteomics data. In both cases, the raw MS data were processed using Proteome Discoverer v2.5 (Thermo Fisher Scientific). While the focus in the workflow presented below is differential protein expression analysis, the data processing and quality control steps described here are applicable to any TMT or LFQ proteomics dataset. Importantly, however, the experimental aim will influence data-guided decisions and the considerations discussed here likely differ from those of spatial proteomics, for example.

### Downloading the data

The files required for this workflow can be found deposited to the ProteomeXchange Consortium via the PRIDE ^15^^,^^16^ partner repository with the dataset identifier PXD041794, Zenodo at http://doi.org/10.5281/zenodo.7837375 and at the Github repository https://github.com/CambridgeCentreForProteomics/f1000_expression_proteomics/. Users are advised to download these files into their current working directory. In R the setwd function can be used to specify a working directory, or if using RStudio one can use the Session -> Set Working Directory menu.

## The infrastructure: QFeatures and SummarizedExperiments

**Figure 2.**
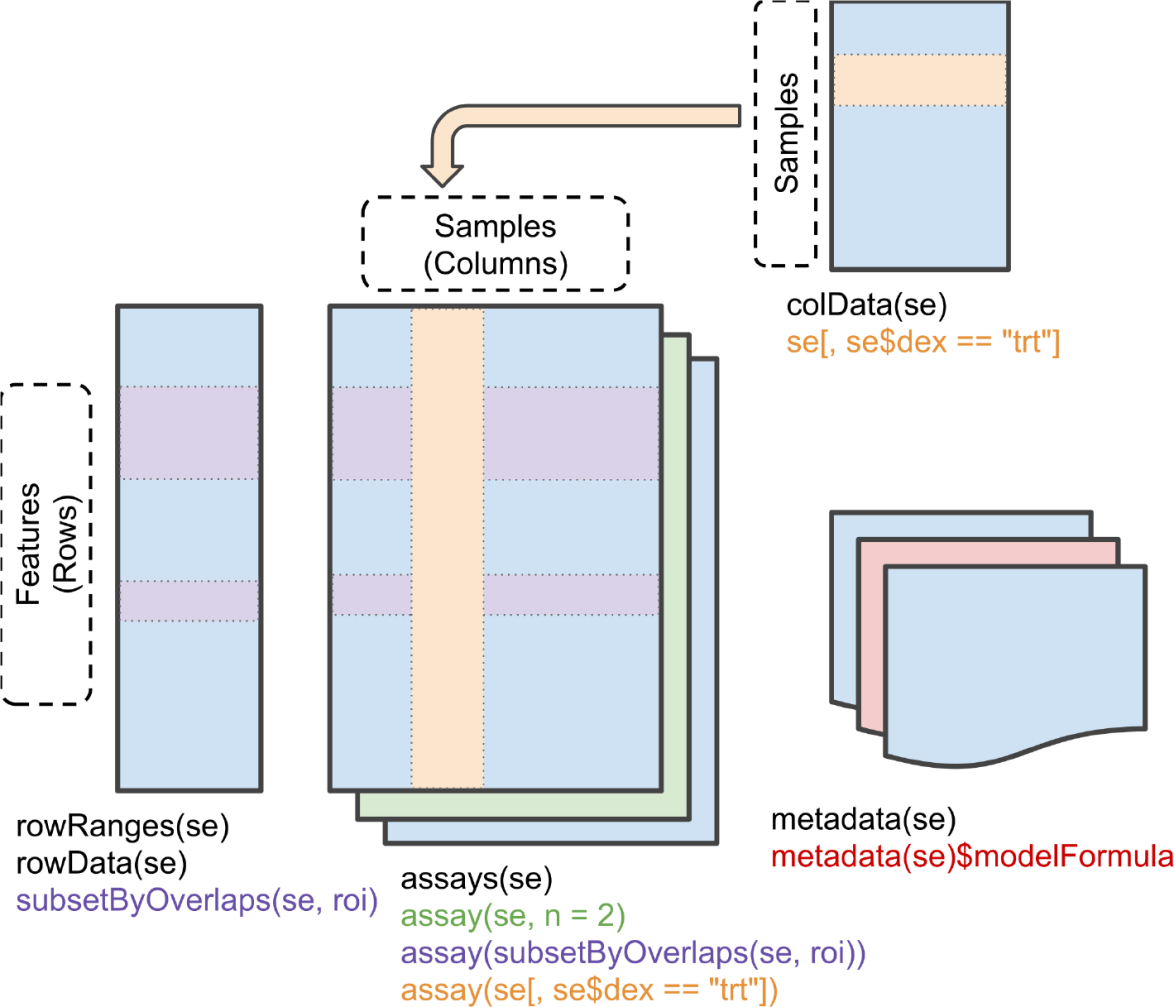
A graphic representation of the SummarizedExperiment (SE) object structure. Figure reproduced from the SummarizedExperiment package ^18^ vignette with permission.

To be able to conveniently track each step of this workflow, users should make use of the Quantitative features for mass spectrometry, or QFeatures, Bioconductor package. ^17^ Prior to utilising the QFeatures infrastructure, it is first necessary to understand the structure of a SummarizedExperiment ^18^ object as QFeatures objects are based on the SummarizedExperiment class. A SummarizedExperiment, often referred to as an SE, is a data container and S4 object comprised of three components: (1) the colData (column data) containing sample metadata, (2) the rowData containing data features, and (3) the assay storing quantitation data, as illustrated in Figure 2. The sample metadata includes annotations such as condition and replicate, and can be accessed using the colData function. Data features, accessed via the rowData function, represent information derived from the identification search. Examples include peptide sequence, master protein accession, and confidence scores. Finally, quantitative data is stored in the assay slot. These three independent data structures are neatly stored within a single SummarizedExperiment object.

**Figure 3.**
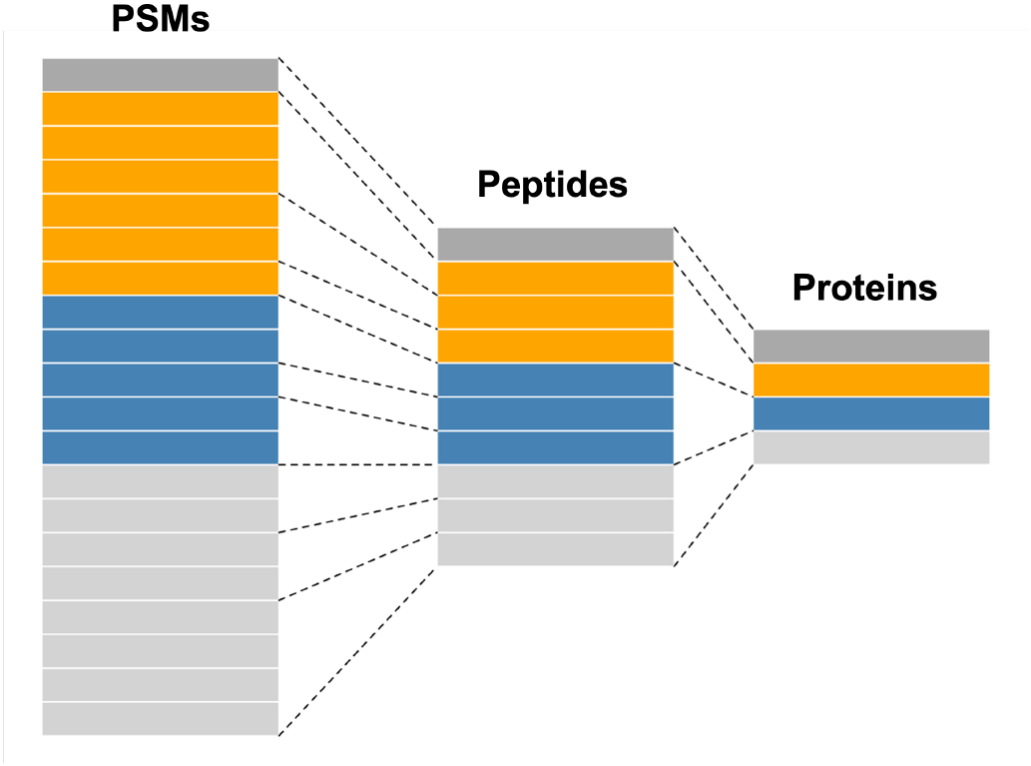
A graphic representation of the QFeatures object structure showing the relationship between assays. Figure modified from the QFeatures ^17^ vignette with permission.

A QFeatures object holds each level of quantitative proteomics data, namely (but not limited to) the PSM, peptide and protein-level data. Each level of the data is stored as its own SummarizedExperiment within a single QFeatures object. The lowest level data e.g. PSM, is first imported into a QFeatures object before aggregating upward towards protein-level ( Figure 3). During this process of aggregation, QFeatures maintains the hierarchical links between quantitative levels whilst allowing easy access to all data levels for individual proteins of interest. This key aspect of QFeatures will be exemplified throughout this workflow. Additional guidance on the use of QFeatures can be found in Ref. 17. For visualisation of the data, all plots are generated using standard ggplot functionality, but could equally be produced using base R.

## Processing and analysing quantitative TMT data

First, we provide a workflow for the processing and quality control of quantitative TMT-labelled data. As outlined above, the cell pellet fractions of triplicate control and treated HEK293 cells were labelled using a TMT6plex. Labelling was as outlined in Table 1.

**Table 1.** TMT labelling strategy in the use-case experiment.

Sample name	Condition	Replicate	Tag
S1	Treated	1	TMT128
S2	Treated	2	TMT127
S3	Treated	3	TMT131
S4	Control	1	TMT129
S5	Control	2	TMT126
S6	Control	3	TMT130

### Identification search of raw data

The first processing step in any MS-based proteomics experiment involves an identification search using the raw data. The aim of this search is to identify which peptide sequences, and therefore proteins, correspond to the raw spectra output from the mass spectrometer. Several third-party software exist to facilitate identification searches of raw MS data but ultimately the output of any search is a list of PSMs, peptides and protein identifications along with their corresponding quantification data.

The use-case data presented here was processed using Proteome Discoverer 2.5 and additional information about this search is provided in an appendix in the GitHub repository https://github.com/CambridgeCentreForProteomics/f1000_expression_proteomics. Further, we provide template workflows for both the processing and consensus steps of the Proteome Discoverer identification runs. It is also possible to determine several of the key parameter settings during the preliminary data exploration. This step will be particularly important for those using publicly available data without detailed knowledge of the identification search parameters. For now, we simply export the PSM-level .txt file from the Proteome Discoverer output.

### Importing data into R and creating a QFeatures object

Data cleaning, exploration and filtering at the PSM-level is performed in R using QFeatures. The function readQFeatures is used to import the PSM-level .txt file. As the cell pellet TMT data we will use is derived from one TMT6plex, only one PSM-level .txt file needs to be imported. This file should be stored within the users working directory.

The columns containing quantitative data also need to be identified before import. To check the column names we use names and read.delim (the equivalent for a .csv file would be read.csv). In the current experiment the order of TMT labels was randomised in an attempt to minimise the effect of TMT channel leakage. For ease of grouping and simplification of downstream visualisation, samples are re-ordered during the import step. This is done by creating a vector containing the sample column names in their correct order. If samples are already in the desired order, the vector can be created by simply indexing the quantitative columns.

## Locate the PSM .txt file
cp_psm <- "cell_pellet_tmt_results_psms.txt"


## Identify columns containing quantitative data
cp_psm %>%
  read.delim() %>%
  names()

##  [1] "PSMs.Workflow.ID"                  "PSMs.Peptide.ID"
##  [3] "Checked"                           "Tags"
##  [5] "Confidence"                        "Identifying.Node.Type"
##  [7] "Identifying.Node"                  "Search.ID"
##  [9] "Identifying.Node.No"               "PSM.Ambiguity"
## [11] "Sequence"                          "Annotated.Sequence"
## [13] "Modifications"                     "Number.of.Proteins"
## [15] "Master.Protein.Accessions"         "Master.Protein.Descriptions"
## [17] "Protein.Accessions"                "Protein.Descriptions"
## [19] "Number.of.Missed.Cleavages"        "Charge"
## [21] "Original.Precursor.Charge"         "Delta.Score"
## [23] "Delta.Cn"                          "Rank"
## [25] "Search.Engine.Rank"                "Concatenated.Rank"
## [27] "mz.in.Da"                          "MHplus.in.Da"
## [29] "Theo.MHplus.in.Da"                 "Delta.M.in.ppm"
## [31] "Delta.mz.in.Da"                    "Ions.Matched"
## [33] "Matched.Ions"                      "Total.Ions"
## [35] "Intensity"                         "Activation.Type"
## [37] "NCE.in.Percent"                    "MS.Order"
## [39] "Isolation.Interference.in.Percent" "SPS.Mass.Matches.in.Percent"
## [41] "Average.Reporter.SN"               "Ion.Inject.Time.in.ms"
## [43] "RT.in.min"                         "First.Scan"
## [45] "Last.Scan"                         "Master.Scans"
## [47] "Spectrum.File"                     "File.ID"
## [49] "Abundance.126"                     "Abundance.127"
## [51] "Abundance.128"                     "Abundance.129"
## [53] "Abundance.130"                     "Abundance.131"
## [55] "Quan.Info"                         "Peptides.Matched"
## [57] "XCorr"                             "Number.of.Protein.Groups"
## [59] "Percolator.q.Value"                "Percolator.PEP"
## [61] "Percolator.SVMScore"

## Store location of quantitative columns in a vector in the desired order
abundance_ordered <- c("Abundance.128",
                       "Abundance.127",
                       "Abundance.131",
                       "Abundance.129",
                       "Abundance.126",
                       "Abundance.130")


Now that the necessary file and its quantitative data columns have been identified, we can pass this to the readQFeatures function and provide these two pieces of information. We also specify that the file is tab-delimited by including sep = “\t” (similarly you would use sep = “,” for a .csv file). Of note, the readQFeatures function can also take fnames as an argument to specify a column to be used as the row names of the imported object. Whilst previous QFeatures vignettes used the “Sequence” or “Annotated.Sequence” as row names, we advise against this because of the presence of PSMs matched to the same peptide sequence with different modifications. In such cases, multiple rows would have the same name forcing the readQFeatures function to output a “making assay row names unique” message and add an identifying number to the end of each duplicated row name. These sequences would then be considered as unique during the aggregation of PSM to peptide, thus resulting in two independent peptide-level quantitation values rather than one. Therefore, we do not pass a fnames argument and the row names automatically become indices. Finally, we pass the name argument to indicate the type of data added.

## Create QFeatures
cp_qf <- readQFeatures(table = cp_psm,
                       ecol = abundance_ordered,
                       sep = "\t",
                       name = "psms_raw")


### Accessing the QFeatures infrastructure

As outlined above, a QFeatures data object is a list of SummarizedExperiment objects. As such, an individual SummarizedExperiment can be accessed using the standard double bracket nomenclature, as demonstrated in the code chunk below.

## Index using position
cp_qf[[1]]

## class: SummarizedExperiment
## dim: 48832 6
## metadata(0):
## assays(1): ”
## rownames(48832): 1 2 … 48831 48832
## rowData names(55): PSMs.Workflow.ID PSMs.Peptide.ID … Percolator.PEP
##   Percolator.SVMScore
## colnames(6): Abundance.128 Abundance.127 … Abundance.126
##   Abundance.130
## colData names(0):

## Index using name
cp_qf[["psms_raw"]]

## class: SummarizedExperiment
## dim: 48832 6
## metadata(0):
## assays(1): ”
## rownames(48832): 1 2 … 48831 48832
## rowData names(55): PSMs.Workflow.ID PSMs.Peptide.ID … Percolator.PEP
##   Percolator.SVMScore
## colnames(6): Abundance.128 Abundance.127 … Abundance.126
##   Abundance.130
## colData names(0):


A summary of the data contained in the slots is printed to the screen. To retrieve the rowData, colData or assay data from a particular SummarizedExperiment within a QFeatures object users can make use of the rowData, colData and assay functions. For plotting or data transformation it is necessary to convert to a data.frame or tibble.

## Access feature information with rowData
## The output should be converted to data.frame/tibble for further processing
cp_qf[["psms_raw"]] %>%
  rowData() %>%
  as_tibble() %>%
  summarise(mean_intensity = mean(Intensity))

## # A tibble: 1 x 1
##    mean_intensity
##             <dbl>
## 1       13281497.


### Adding metadata

Having imported the data, each sample is first annotated with its TMT label, sample reference and condition. As this information is experimental metadata, it is added to the colData slot. It is also useful to clean up sample names such that they are short, intuitive and informative. This is done by editing the colnames. These steps may not always be necessary depending upon the identification search output.

## Clean sample names
colnames(cp_qf[["psms_raw"]]) <- paste0("S", 1:6)

## Add sample info as colData to QFeatures object
cp_qf$label <- c("TMT128",
                 "TMT127",
                 "TMT131",
                 "TMT129",
                 "TMT126",
                 "TMT130")

cp_qf$sample <- paste0("S", 1:6)

cp_qf$condition <- rep(c("Treated", "Control"), each = 3)

## Verify
colData(cp_qf)

## DataFrame with 6 rows and 3 columns
##           label        sample     condition
##     <character>   <character>   <character>
## S1       TMT128            S1       Treated
## S2       TMT127            S2       Treated
## S3       TMT131            S3       Treated
## S4       TMT129            S4       Control
## S5       TMT126            S5       Control
## S6       TMT130            S6       Control

## Assign the colData to first assay as well
colData(cp_qf[["psms_raw"]]) <- colData(cp_qf)


### Preliminary data exploration

As well as cleaning and annotating the data, it is always advisable to check that the import worked and that the data looks as expected. Further, preliminary exploration of the data can provide an early sign of whether the experiment and subsequent identification search were successful. Importantly, however, the names of key parameters will vary depending on the software used, and will likely change over time. Users will need to be aware of this and modify the code in this workflow accordingly.

## Check what information has been imported
cp_qf[["psms_raw"]] %>%
  rowData() %>%
  colnames()

##  [1] "PSMs.Workflow.ID"                  "PSMs.Peptide.ID"
##  [3] "Checked"                           "Tags"
##  [5] "Confidence"                        "Identifying.Node.Type"
##  [7] "Identifying.Node"                  "Search.ID"
##  [9] "Identifying.Node.No"               "PSM.Ambiguity"
## [11] "Sequence"                          "Annotated.Sequence"
## [13] "Modifications"                     "Number.of.Proteins"
## [15] "Master.Protein.Accessions"         "Master.Protein.Descriptions"
## [17] "Protein.Accessions"                "Protein.Descriptions"
## [19] "Number.of.Missed.Cleavages"        "Charge"
## [21] "Original.Precursor.Charge"         "Delta.Score"
## [23] "Delta.Cn"                          "Rank"
## [25] "Search.Engine.Rank"                "Concatenated.Rank"
## [27] "mz.in.Da"                          "MHplus.in.Da"
## [29] "Theo.MHplus.in.Da"                 "Delta.M.in.ppm"
## [31] "Delta.mz.in.Da"                    "Ions.Matched"
## [33] "Matched.Ions"                      "Total.Ions"
## [35] "Intensity"                         "Activation.Type"
## [37] "NCE.in.Percent"                    "MS.Order"
## [39] "Isolation.Interference.in.Percent" "SPS.Mass.Matches.in.Percent"
## [41] "Average.Reporter.SN"               "Ion.Inject.Time.in.ms"
## [43] "RT.in.min"                         "First.Scan"
## [45] "Last.Scan"                         "Master.Scans"
## [47] "Spectrum.File"                     "File.ID"
## [49] "Quan.Info"                         "Peptides.Matched"
## [51] "XCorr"                             "Number.of.Protein.Groups"
## [53] "Percolator.q.Value"                "Percolator.PEP"
## [55] "Percolator.SVMScore"

## Find out how many PSMs are in the data
cp_qf[["psms_raw"]] %>%
  dim()

##  [1]  48832  6

original_psms <- cp_qf[["psms_raw"]] %>%
  nrow() %>%
  as.numeric()


We can see that the original data includes 48832 PSMs across the 6 samples. It is also useful to make note of how many peptides and proteins the raw PSM data corresponds to, and to track how many we remove during the subsequent filtering steps. This can be done by checking how many unique entries are located within the “Sequence” and “Master.Protein.Accessions” for peptides and proteins, respectively. Of note, searching for unique peptide sequences means that the number of peptides does not include duplicated sequences with different modifications.

## Find out how many peptides and master proteins are in the data
original_peps <- cp_qf[["psms_raw"]] %>%
  rowData() %>%
  as_tibble() %>%
  pull(Sequence) %>%
  unique() %>%
  length() %>%
  as.numeric()

original_prots <- cp_qf[["psms_raw"]] %>%
  rowData() %>%
  as_tibble() %>%
  pull(Master.Protein.Accessions) %>%
  unique() %>%
  length() %>%
  as.numeric()

print(c(original_peps, original_prots))

##  [1]  25969  5040


Hence, the output of the identification search contains 48832 PSMs corresponding to 25969 peptide sequences and 5040 master proteins. Finally, we confirm that the identification search was carried out as expected. For this, we print summaries of the key search parameters using the table function for discrete parameters and summary for those which are continuous. This is also helpful for users who are analysing publicly available data and have limited knowledge about the identification search parameters.

## Check missed cleavages
cp_qf[["psms_raw"]] %>%
  rowData() %>%
  as_tibble() %>%
  pull(Number.of.Missed.Cleavages) %>%
  table()

## .
##     0     1    2
## 46164  2592   76

## Check precursor mass tolerance
cp_qf[["psms_raw"]] %>%
  rowData() %>%
  as_tibble() %>%
  pull(Delta.M.in.ppm) %>%
  summary()

##    Min.   1st Qu.  Median    Mean  3rd Qu.   Max.
## -8.9300  -0.6000  0.3700  0.6447  1.3100  9.6700

## Check fragment mass tolerance
cp_qf[["psms_raw"]] %>%
  rowData() %>%
  as_tibble() %>%
  pull(Delta.mz.in.Da) %>%
  summary()

##       Min.      1st Qu.    Median       Mean     3rd Qu.      Max.
## -0.0110400  -0.0004100  0.0002500  0.0006812  0.0010200  0.0135100

## Check PSM confidence allocations
cp_qf[["psms_raw"]] %>%
  rowData() %>%
  as_tibble() %>%
  pull(Confidence) %>%
  table()

## .
##   High
##  48832


### Experimental quality control checks

Experimental quality control of TMT-labelled quantitive proteomics data takes place in two steps: (1) assessment of the raw mass spectrometry data, and (2) evaluation of TMT labelling efficiency.

### Quality control of the raw mass spectrometry data

Having taken an initial look at the output of the identification search, it is possible to create some simple plots to inspect the raw mass spectrometry data. Such plots are useful in revealing problems that may have occurred during the mass spectrometry run but are far from extensive. Users who wish to carry out a more in-depth evaluation of the raw mass spectrometry data may benefit from use of the Spectra Bioconductor package which allows for visualisation and exploration of raw chromatograms and spectra, among other features. ^19^

The first plot we generate looks at the delta precursor mass, that is the difference between observed and estimated precursor mass, across retention time. Importantly, exploration of this raw data feature can only be done when using the raw data prior to recalibration. For users of Proteome Discoverer, this means using the spectral files node rather than the spectral files recalibration node.

## Generate scatter plot of mass accuracy
cp_qf[["psms_raw"]] %>%
  rowData() %>%
  as_tibble() %>%
  ggplot(aes(x = RT.in.min, y = Delta.M.in.ppm)) +
  geom_point(size = 0.5, shape = 4) +
  geom_hline(yintercept = 5, linetype = "dashed", color = "red") +
  geom_hline(yintercept = -5, linetype = "dashed", color = "red") +
  labs(x = "RT (min)", y = "Delta precursor mass (ppm)") +
  scale_x_continuous(limits = c(0, 120), breaks = seq(0, 120, 20)) +
  scale_y_continuous(limits = c(-10, 10), breaks = c(-10, -5, 0, 5, 10)) +
  ggtitle("PSM retention time against delta precursor mass") +
  theme_bw()

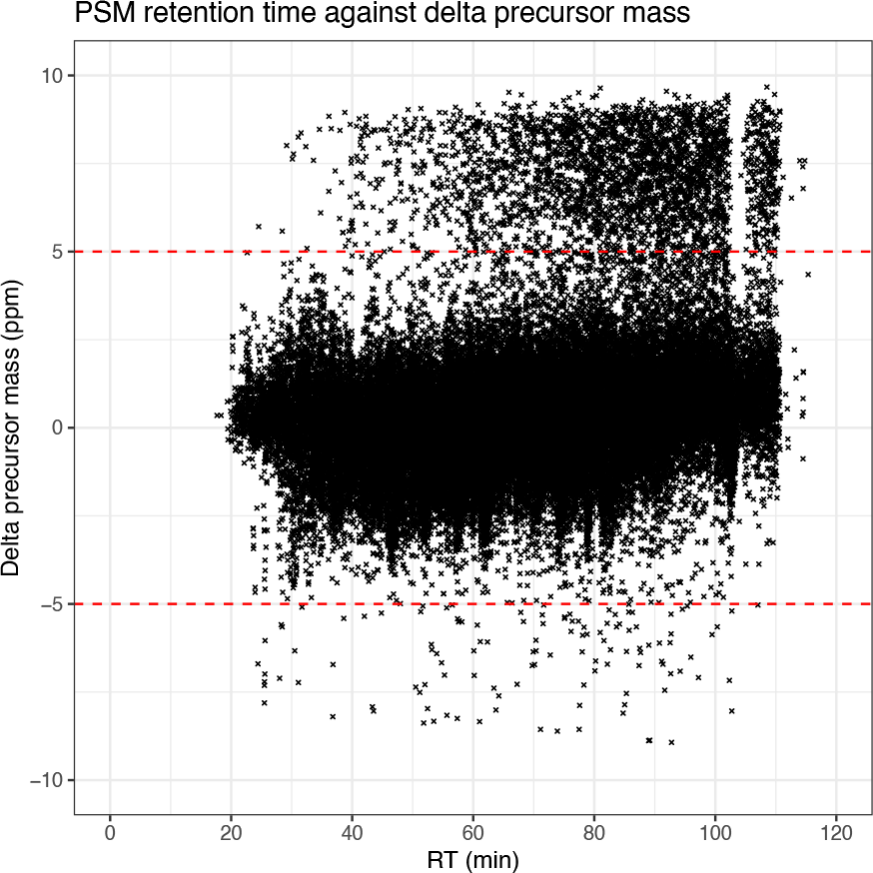


Since we applied a precursor mass tolerance of 10 ppm during the identification search, all of the PSMs are within ±10 ppm. Ideally, however, we want the majority of the data to be within ±5 ppm since smaller delta masses correspond to a greater rate of correct peptide identifications. From the graph we have plotted we can see that indeed the majority of PSMs are within ±5 ppm. If users find that too many PSMs are outside of the desired ±5 ppm, it is advisable to check the calibration of the mass spectrometer.

The second quality control plot of raw data is that of MS2 ion inject time across the retention time gradient. Here, it is desirable to achieve an average MS2 injection time of 50 ms or less, although the exact target threshold will depend upon the sample load. If the average ion inject time is longer than desired, then the ion transfer tube and/or front end optics of the instrument may require cleaning.

## Generate scatter plot of ion inject time across retention time
cp_qf[["psms_raw"]] %>%
  rowData() %>%
  as_tibble() %>%
  ggplot(aes(x = RT.in.min, y = Ion.Inject.Time.in.ms)) +
  geom_point(size = 0.5, shape = 4) +
  geom_hline(yintercept = 50, linetype = "dashed", color = "red") +
  labs(x = "RT (min)", y = "Ion inject time (ms)") +
  scale_x_continuous(limits = c(0, 120), breaks = seq(0, 120, 20)) +
  scale_y_continuous(limits = c(0, 60), breaks = seq(0, 60, 10)) +
  ggtitle("PSM retention time against ion inject time") +
  theme_bw()

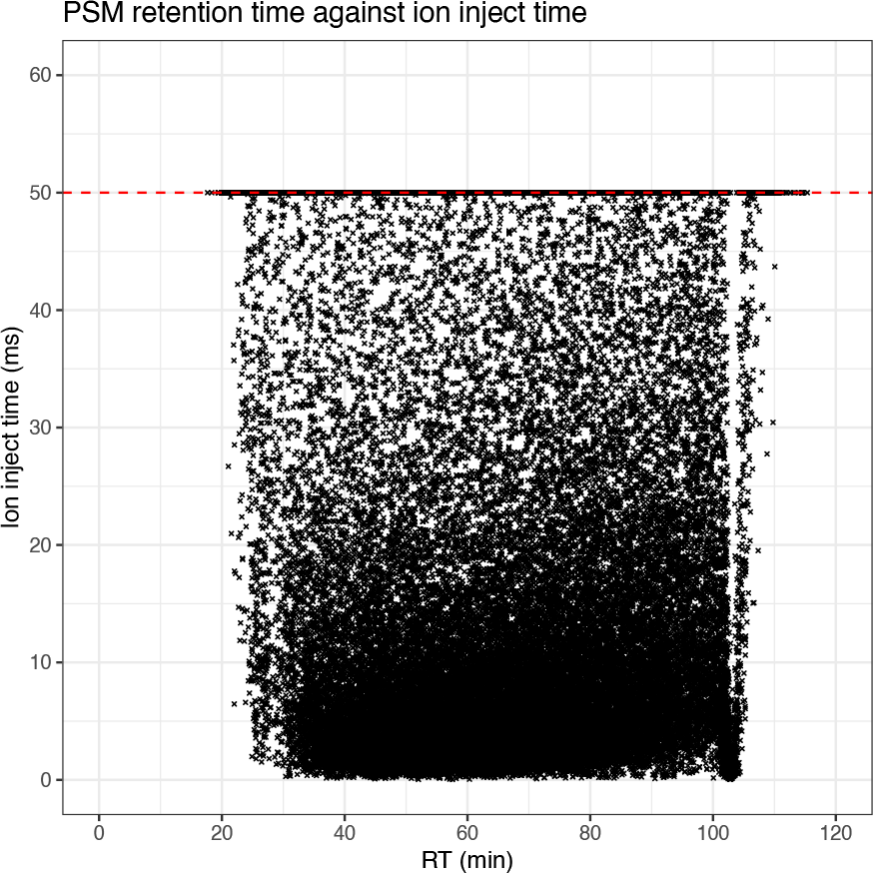


From this plot we can see that whilst there is a high density of PSMs at low inject times, there are also many data points found at the 50 ms threshold. This indicates that by increasing the time allowed for ions to accumulate in the ion trap, the number of PSMs could also have been increased. Finally, we inspect the distribution of PSMs across both the ion injection time and retention time by plotting histograms.

## Plot histogram of PSM ion inject time
cp_qf[["psms_raw"]] %>%
  rowData() %>%
  as_tibble() %>%
  ggplot(aes(x = Ion.Inject.Time.in.ms)) +
  geom_histogram(binwidth = 1) +
  labs(x = "Ion inject time (ms)", y = "Frequency") +
  scale_x_continuous(limits = c(-0.5, 52.5), breaks = seq(0, 50, 5)) +
  ggtitle("PSM frequency across ion injection time") +
  theme_bw()

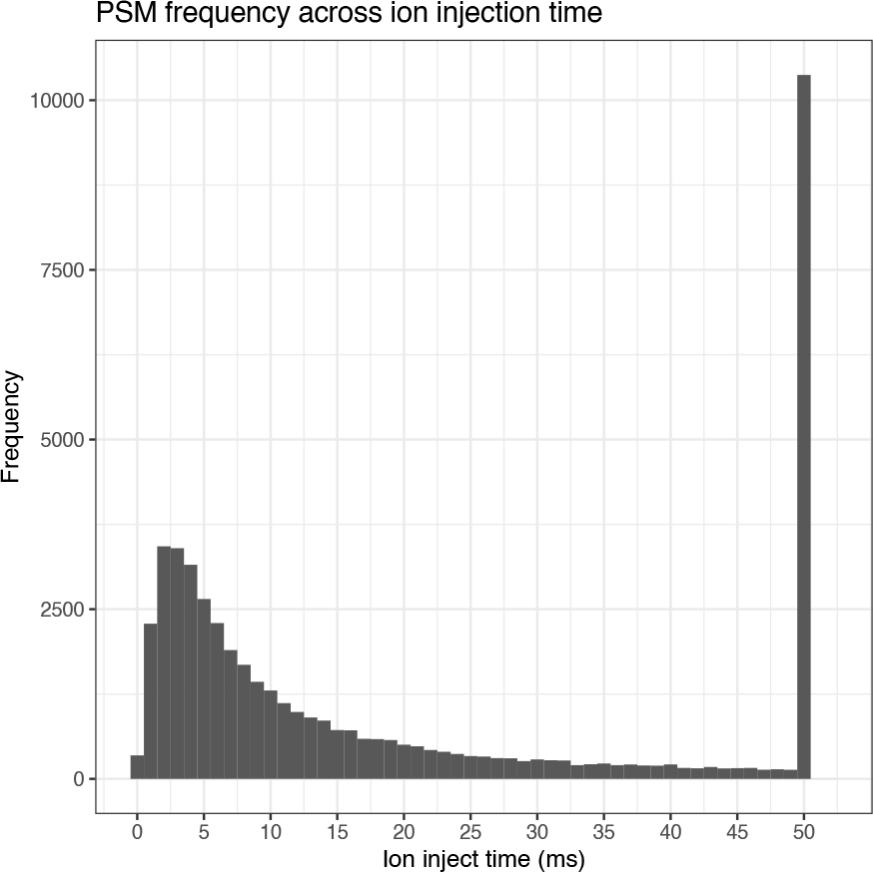


## Plot histogram of PSM retention time
cp_qf[["psms_raw"]] %>%
  rowData() %>%
  as_tibble() %>%
  ggplot(aes(x = RT.in.min)) +
  geom_histogram(binwidth = 1) +
  labs(x = "RT (min)", y = "Frequency") +
  scale_x_continuous(breaks = seq(0, 120, 20)) +
  ggtitle("PSM frequency across retention time") +
  theme_bw()

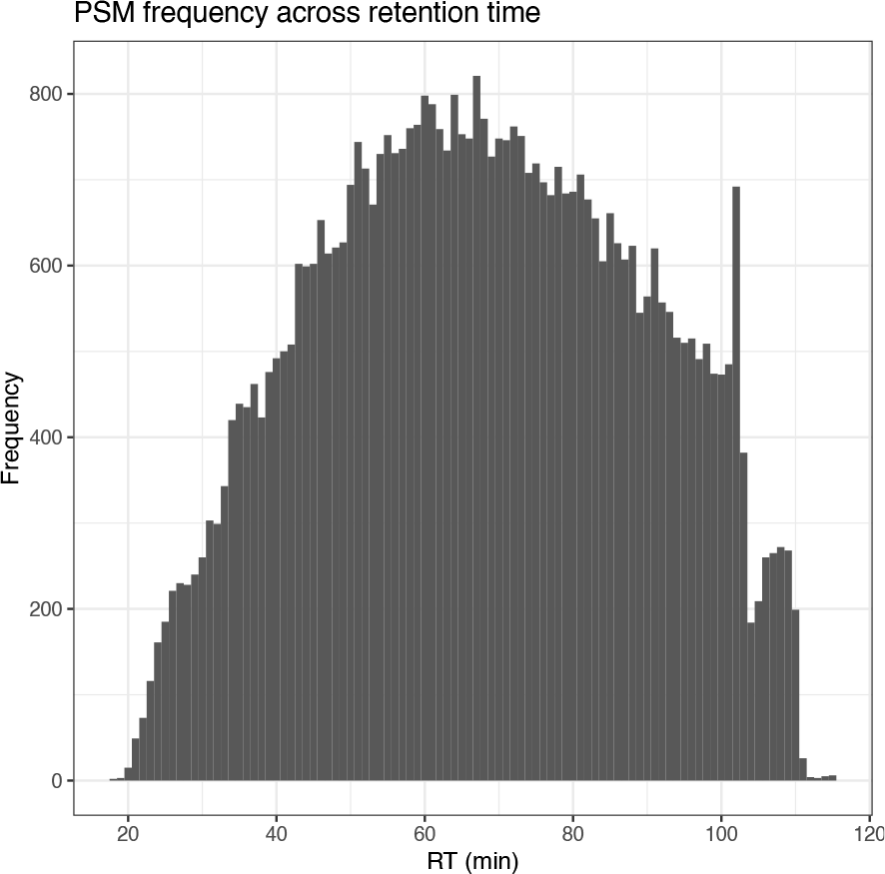


The four plots that we have generated look relatively standard with no obvious problems indicated. Therefore, we continue by evaluating the quality of the processed data.

### Checking the efficiency of TMT labelling

The most fundamental data quality control step in a TMT experiment is to check the TMT labelling efficiency. TMT labels react with amine groups present at the peptide N-terminus as well as the side chain of lysine (K) residues. Of note, lysine residues can be TMT modified regardless of whether they are present at the C-terminus of a trypic peptide or internally following miscleavage.

To evaluate the TMT labelling efficiency, a separate identification search of the raw data was completed with lysine (K) and peptide N-termini TMT labels considered as dynamic modifications rather than static. No additional residues (S or T) were evaluated for labelling in the search. This allows the search engine to assess the presence of both the modified (TMT labelled) and unmodified (original) forms of each peptide. The relative proportions of modified and unmodified peptides can then be used to calculate the TMT labelling efficiency. To demonstrate how to check for TMT labelling efficiency, only two of the eight fractions were utilised for this search.

As we will only look at TMT efficiency at the PSM-level, here we upload the .txt file directly as a SummarizedExperiment rather than a QFeatures object. This is done using the readSummarizedExperiment function and the same arguments as those in readQFeatures.

## Locate the PSM .txt file
tmt_psm <- "cell_pellet_tmt_efficiency_psms.txt"

## Identify columns containing quantitative data
tmt_psm %>%
  read.delim() %>%
  names()

##  [1] "PSMs.Workflow.ID"                  "PSMs.Peptide.ID"
##  [3] "Checked"                           "Tags"
##  [5] "Confidence"                        "Identifying.Node.Type"
##  [7] "Identifying.Node"                  "Search.ID"
##  [9] "Identifying.Node.No"               "PSM.Ambiguity"
## [11] "Sequence"                          "Annotated.Sequence"
## [13] "Modifications"                     "Number.of.Proteins"
## [15] "Master.Protein.Accessions"         "Master.Protein.Descriptions"
## [17] "Protein.Accessions"                "Protein.Descriptions"
## [19] "Number.of.Missed.Cleavages"        "Charge"
## [21] "Original.Precursor.Charge"         "Delta.Score"
## [23] "Delta.Cn"                          "Rank"
## [25] "Search.Engine.Rank"                "Concatenated.Rank"
## [27] "mz.in.Da"                          "MHplus.in.Da"
## [29] "Theo.MHplus.in.Da"                 "Delta.M.in.ppm"
## [31] "Delta.mz.in.Da"                    "Ions.Matched"
## [33] "Matched.Ions"                      "Total.Ions"
## [35] "Intensity"                         "Activation.Type"
## [37] "NCE.in.Percent"                    "MS.Order"
## [39] "Isolation.Interference.in.Percent" "SPS.Mass.Matches.in.Percent"
## [41] "Average.Reporter.SN"               "Ion.Inject.Time.in.ms"
## [43] "RT.in.min"                         "First.Scan"
## [45] "Last.Scan"                         "Master.Scans"
## [47] "Spectrum.File"                     "File.ID"
## [49] "Abundance.126"                     "Abundance.127"
## [51] "Abundance.128"                     "Abundance.129"
## [53] "Abundance.130"                     "Abundance.131"
## [55] "Quan.Info"                         "Peptides.Matched"
## [57] "XCorr"                             "Number.of.Protein.Groups"
## [59] "Contaminant"                       "Percolator.q.Value"
## [61] "Percolator.PEP"                    "Percolator.SVMScore"

## Read in as a SummarizedExperiment
tmt_se <- readSummarizedExperiment(table = tmt_psm,
                                   ecol = abundance_ordered,
                                   sep = "\t")

## Clean sample names
colnames(tmt_se) <- paste0("S", 1:6)

## Add sample info as colData to QFeatures object
tmt_se$label <- c("TMT128",
                  "TMT127",
                  "TMT131",
                  "TMT129",
                  "TMT126",
                  "TMT130")

tmt_se$sample <- paste0("S", 1:6)

tmt_se$condition <- rep(c("Treated", "Control"), each = 3)

## Verify
colData(tmt_se)

## DataFrame with 6 rows and 3 columns
##          label      sample   condition
##    <character> <character> <character>
## S1      TMT128          S1     Treated
## S2      TMT127          S2     Treated
## S3      TMT131          S3     Treated
## S4      TMT129          S4     Control
## S5      TMT126          S5     Control
## S6      TMT130          S6     Control


Information about the presence of labels is stored within the ‘Modifications’ feature of the rowData. Using this information, the TMT labelling efficiency of the experiment is calculated using the code chunks below. Users should alter this code if TMTpro reagents are being used such that “TMT6plex” is replaced by “TMTpro”.

First we consider the efficiency of peptide N-termini TMT labelling. We use the grep function to identify PSMs which are annotated as having an N-Term TMT6plex modification. We then calculate the number of PSMs with this annotation as a proportion of the total number of PSMs.

## Count the total number of PSMs
tmt_total <- length(tmt_se)

## Count the number of PSMs with an N-terminal TMT modification
nterm_labelled_rows <- grep("N-Term\\(TMT6plex\\)",
                            rowData(tmt_se)$Modifications)
nterm_psms_labelled <- length(nterm_labelled_rows)

## Calculate N-terminal TMT labelling efficiency
efficiency_nterm <- (nterm_psms_labelled / tmt_total) * 100

efficiency_nterm %>%
  round(digits = 1) %>%
  print()

## [1] 96.8


Secondly, we consider the TMT labelling efficiency of lysine (K) residues. As mentioned above, lysine residues can be TMT labelled regardless of their position within a peptide. Hence, we here calculate lysine labelling efficiency on a per lysine residue basis.

## Count the number of lysine TMT6plex modifications in the PSM data
k_tmt <- str_count(string = rowData(tmt_se)$Modifications,
                   pattern = "K[0-9]{1,2}\\(TMT6plex\\)") %>%
  sum() %>%
  as.numeric()

## Count the number of lysine residues in the PSM data
k_total <- str_count(string = rowData(tmt_se)$Sequence,
                     pattern = "K") %>%
  sum() %>%
  as.numeric()

## Determine the percentage of TMT labelled lysines
efficiency_k <- (k_tmt / k_total) * 100

efficiency_k %>%
  round(digits = 1) %>%
  print()

## [1] 98.5


Users should aim for an overall TMT labelling efficiency >90% in order to achieve reliable quantitation. In cases where labelling efficiency is towards the lower end of the acceptable range, TMT labels should be set as dynamic modifications during the final identification search, although this will increase the search space and time as well as influencing false discovery rate (FDR) calculations. A summary of the current advice from Thermo Fisher is provided in Table 2. Where labelling efficiency is calculated as being between categories, how to progress is ultimately decided by the user.

**Table 2.** ThermoFisher search strategy recommendations based on TMT labelling efficiency.

N-term efficiency	K efficiency	Suggested search method
>98%	>98%	Both modifications as ’static’
85-95%	>98%	N-terminal modification ’dynamic’ and K modification ’static’
<84%	<84%	Data not suitable for quantitation

Since the use-case data has a sufficiently high TMT labelling efficiency, we can continue to use the output of the identification search. This search considered TMT labelling of lysines as a static modification whilst N-terminal labelling was kept as dynamic, to investigate the presence of protein N-terminal modifications.

### Basic data cleaning

Being confident that the experiment and identification search were successful, we can now begin with some basic data cleaning. However, we also want to keep a copy of the raw PSM data. Therefore, we first create a second copy of the PSM SummarizedExperiment, called “psms_filtered”, and add it to the QFeatures object. This is done using the addAssay function. All changes made at the PSM-level will then only be applied to this second copy, so that we can refer back to the original data if needed.

## Extract the "psms_raw" SummarizedExperiment
data_copy <- cp_qf[["psms_raw"]]

## Add copy of SummarizedExperiment
cp_qf <- addAssay(x = cp_qf,
                  y = data_copy,
                  name = "psms_filtered")

## Verify
cp_qf

## An instance of class QFeatures containing 2 assays:
## [1] psms_raw: SummarizedExperiment with 48832 rows and 6 columns
## [2] psms_filtered: SummarizedExperiment with 48832 rows and 6 columns


Of note, manually adding an assay (or SummarizedExperiment) to the QFeatures object does not automatically generate links between these assays. We will manually add the explicit links later, after we complete data cleaning and filtering.

The cleaning steps included in this section are non-specific and should be applied to all quantitative proteomics datasets. The names of key parameters will vary in data outputs from alternative third party software, however, and users should remain aware of both terminology changes over time as well as the introduction of new filters. All data cleaning steps are completed in the same way. We first determine how many rows, here PSMs, meet the conditions for removal. This is achieved by using the dplyr::count function. The unwanted rows are removed using the filterFeatures function. Since we only wish to apply the filters to the “psms_filtered” level, we specify this by using the i = argument. If this argument is not used, filterFeatures will remove features from all assays within a QFeatures object.

### Removing PSMs not matched to a master protein

The first common cleaning step we carry out is the removal of PSMs that have not been assigned to a master protein during the identification search. This can happen when the search software is unable to resolve conflicts caused by the presence of the isobaric amino acids leucine and isoleucine. Before implementing the filter, it is useful to find out how many PSMs we expect to remove. This is easily done by using the dplyr::count on the master protein column. Any master proteins that return TRUE will be removed by filtering. If this returns no TRUE values, users should move on to the next filtering step without removing rows as this will introduce an error.

## Find out how many PSMs we expect to lose
cp_qf[["psms_filtered"]] %>%
  rowData() %>%
  as_tibble() %>%
  dplyr::count(Master.Protein.Accessions == "")

## # A tibble: 2 x 2
##   ‘Master.Protein.Accessions == ""‘ n
##   <lgl>                         <int>
## 1 FALSE                         48660
## 2 TRUE                            172


For users who wish to explicitly track the process of data cleaning, the code chunk below demonstrates how to print a message containing the number of features removed.

paste("Removing",
      length(which(rowData(
        cp_qf[["psms_filtered"]])$Master.Protein.Accessions == "")),
      "PSMs without a master protein accession") %>%
  message()

## Removing 172 PSMs without a master protein accession


This code could be adapted to each cleaning and filtering step. To maintain simplicity of this workflow, we will not print explicit messages at each step. Instead, the decision to do so is left to the user.

## Remove PSMs without a master protein accession using filterFeatures
cp_qf <- cp_qf %>%
  filterFeatures(~ !Master.Protein.Accessions == "",
                 i = "psms_filtered")


### Removing PSMs matched to a contaminant protein

Next we remove PSMs corresponding to contaminant proteins. Such proteins can be introduced intentionally as reagents during sample preparation, as is the case for digestive enzymes, or accidentally, as seen with human keratins derived from skin and hair. Since these proteins do not contribute to the biological question being asked and it is standard practice to remove them from the data. This is done by using a carefully curated, sample-specific contaminant database. Critically, the database used for filtering should be the same one that was used during the identification search. Whilst it is possible to remove contaminants using the filterFeatures function on a contaminants annotation column (as per the QFeatures processing vignette), we demonstrate how to filter using only contaminant protein accessions for users who do not have contaminant annotations within their identification data.

For this experiment, a contaminant database from Ref. 20 was used. The .fasta file for this database is available at the Hao Group’s Github Repository for Protein Contaminant Libraries for DDA and DIA Proteomics and specifically can be found at https://github.com/HaoGroup-ProtContLib/Protein-Contaminant-Libraries-for-DDA-and-DIA-Proteomics/tree/main/Universal%20protein%20contaminant%20FASTA. Here, we import this file using the fasta.index function from the Biostrings package. ^21^ This function requires a file path to the .fasta file and then asks users to specify the sequence type. In this case we have amino acid sequences so pass seqtype = "AA". The function returns a data.frame with one row per FASTA entry. We then can extract the protein accessions from the fasta file. Users will need to alter the below code according to the contaminant file used.

## Load Hao group .fasta file used in search
cont_fasta <- "220813_universal_protein_contaminants_Haogroup.fasta"
conts <- Biostrings::fasta.index(cont_fasta, seqtype = "AA")

## Extract only the protein accessions (not Cont_ at the start)
cont_acc <- regexpr("(?<=\\_).*?(?=\\|)", conts$desc, perl = TRUE) %>%
  regmatches(conts$desc, .)


Now we have our contaminant list by accession number, we can identify and remove PSMs with any contaminant protein within their “Protein.Accessions”. Importantly, filtering on “Protein.Accessions” ensures the removal of PSMs which matched to a protein group containing a contaminant protein, even if the contaminant protein is not the group’s master protein.

## Define function to find contaminants
find_cont <- function(se, cont_acc) {
  cont_indices <- c()
  for (i in 1:length(cont_acc)) {
    cont_protein <- cont_acc[i]
    cont_present <- grep(cont_protein, rowData(se)$Protein.Accessions)
    output <- c(cont_present)
    cont_indices <- append(cont_indices, output)
  }
  cont_psm_indices <- cont_indices
}

## Store row indices of PSMs matched to a contaminant-containing protein group
cont_psms <- find_cont(cp_qf[["psms_filtered"]], cont_acc)

## If we find contaminants, remove these rows from the data
if (length(cont_psms) > 0)
  cp_qf[["psms_filtered"]] <- cp_qf[["psms_filtered"]][-cont_psms, ]


At this point, users can also remove any additional proteins which may not have been included in the contaminant database. For example, users may wish to remove human trypsin (accession P35050) should it appear in their data.

Several third party softwares also have the option to directly annotate which fasta file (here, the human proteome or contaminant database) a PSM is derived from. In such cases, filtering can be simplified by removing PSMs annotated as contaminants in the output file.

### Removing PSMs which lack quantitative data

Now that we are left with only PSMs matched to proteins of interest, we filter out PSMs which cannot be used for quantitation. This includes some PSMs which lack quantitative information altogether. In outputs derived from Proteome Discoverer this information is included in the “Quan.Info” column where PSMs are annotated as having “NoQuanLabels”. For users who have considered both lysine and N-terminal TMT labels as static modifications, the data should not contain any PSMs without quantitative information. However, since the use-case data was derived from a search in which N-terminal TMT modifications were dynamic, the data does include this annotation. Users are reminded that column names are typically software-specific as the “Quan.Info” column is found only in outputs derived from Proteome Discoverer. However, the majority of alternative third party softwares will have an equivalent column containing the same information.

## Find out how many PSMs we expect to lose
cp_qf[["psms_filtered"]] %>%
  rowData() %>%
  as_tibble() %>%
  dplyr::count(Quan.Info == "NoQuanLabels")

## # A tibble: 2 x 2
##  ‘Quan.Info == "NoQuanLabels"‘    n
##  <lgl>                        <int>
## 1 FALSE                       47241
## 2 TRUE                          228

## Drop these rows from the data
cp_qf <- cp_qf %>%
  filterFeatures(~ !Quan.Info == "NoQuanLabels",
                 i = "psms_filtered")


This point in the workflow is a good time to check whether there are any other annotations within the “Quan.Info” column. For example, if there are any PSMs which have been “ExcludedByMethod”, this indicates that a PSM-level filter was applied in Proteome Discoverer during the identification search. If this is the case, users should determine which filter has been applied to the data and decide whether to remove the PSMs which were “ExcludedByMethod” (thereby applying the pre-set threshold) or leave them in (disregard the threshold).

## Are there any remaining annotations in the Quan.Info column?
cp_qf[["psms_filtered"]] %>%
  rowData() %>%
  as_tibble() %>%
  pull(Quan.Info) %>%
  table()

## .
##
## 47241


In the above code chunk we see there are no remaining annotations in the “Quan.Info” column so we can continue.

### Removing PSMs which are not unique to a protein

The next step is to consider which PSMs are to be used for quantitation. There are two ways in which a PSM can be considered as unique. The first and most pure form of uniqueness comes from a PSM corresponding to a single protein only. This results in the PSM being allocated to one protein and one protein group. However, it is common to expand the definition of unique to include PSMs that map to multiple proteins within a single protein group. That is PSMs which are allocated to more than one protein but only one protein group. This distinction is ultimately up to the user. By contrast, PSMs corresponding to razor and shared peptides are linked to multiple proteins across multiple protein groups. In this workflow, the final grouping of peptides to proteins will be done based on master protein accession. Therefore, differential expression analysis will be based on protein groups, and we here consider unique as any PSM linked to only one protein group. This means removing PSMs where “Number.of.Protein.Groups” is not equal to 1.

In the below code chunk we count the number of PSMs linked to more than 1 protein group.

## Find out how many PSMs we expect to lose
cp_qf[["psms_filtered"]] %>%
  rowData() %>%
  as_tibble() %>%
  dplyr::count(Number.of.Protein.Groups != 1)

## # A tibble: 2 x 2
##   ‘Number.of.Protein.Groups != 1‘     n
##   <lgl>                           <int>
## 1 FALSE                           44501
## 2 TRUE                             2740


We again use the filterFeatures function to retain PSMs linked to only 1 protein group and discard any PSMs linked to more 1 group.

## Remove these rows from the data
cp_qf <- cp_qf %>%
  filterFeatures(~ Number.of.Protein.Groups == 1,
                 i = "psms_filtered")


**Additional considerations regarding protein isoforms**

Users searching against a database that includes protein isoforms must take extra caution when defining ‘unique’ PSMs. A PSM that corresponds to a single protein when data is searched against the proteome without isoforms may correspond to multiple proteins once additional isoforms are included. As a result, PSMs or peptides that were previously mapped to one protein and one protein group could instead be mapped to multiple proteins and one protein group. These PSMs would be filtered out by defining ‘unique’ as corresponding to only one protein and one protein group, but would be retained if the definition was expanded to multiple proteins and one protein group. Users should be aware of these possibilities and select their filtering strategy based on the biological question of interest.

### Removing PSMs that are not rank 1

Another filter that is important for quantitation is that of PSM rank. Since individual spectra can have multiple candidate peptide matches, Proteome Discoverer uses a scoring algorithm to determine the probability of a PSM being incorrect. Once each candidate PSM has been given a score, the one with the lowest score (lowest probability of being incorrect) is allocated rank 1. The PSM with the second lowest probability of being incorrect is rank 2, and so on. For the analysis, we only want rank 1 PSMs to be retained.

## Find out how many PSMs we expect to lose
cp_qf[["psms_filtered"]] %>%
  rowData() %>%
  as_tibble() %>%
  dplyr::count(Rank != 1)

## # A tibble: 2 x 2
##  ‘Rank != 1‘      n
##  <lgl>        <int>
## 1 FALSE       43426
## 2 TRUE         1075

## Drop these rows from the data
cp_qf <- cp_qf %>%
  filterFeatures(~ Rank == 1,
                 i = "psms_filtered")


The majority of search engines, including SequestHT, also provide their own PSM rank. To be conservative and ensure accurate quantitation, we also only retain PSMs that have a search engine rank of 1.

## Find out how many PSMs we expect to lose
cp_qf[["psms_filtered"]] %>%
  rowData() %>%
  as_tibble() %>%
  dplyr::count(Search.Engine.Rank != 1)

## # A tibble: 2 x 2
##   ‘Search.Engine.Rank != 1‘     n
##   <lgl>                     <int>
## 1 FALSE                     43153
## 2 TRUE                        273

## Drop these rows from the data
cp_qf <- cp_qf %>%
  filterFeatures(~ Search.Engine.Rank == 1,
                 i = "psms_filtered")


### Removing ambiguous PSMs

Finally, we retain only unambiguous PSMs. Since there are several candidate peptides for each spectra, Proteome Discoverer allocates each PSM a level of ambiguity to indicate whether it was possible to determine a definite PSM or whether one had to be selected from a number of candidates. The allocation of PSM ambiguity takes place during the process of protein grouping and the definitions of each ambiguity assignment are given below in Table 3.

**Table 3.** Definitions of PSM ambiguity categories based on Proteome Discoverer outputs.

PSM category	Definition
Unambiguous	The only candidate PSM
Selected	PSM was selected from a group of candidates
Rejected	PSM was rejected from a group of candidates
Ambiguous	Two or more candidate PSMs could not be distinguished
Unconsidered	PSM was not considered suitable

Importantly, depending upon the software being used, output files may already have excluded some of these categories. It is still good to check before proceeding with the data.

## Find out how many PSMs we expect to lose
cp_qf[["psms_filtered"]] %>%
  rowData() %>%
  as_tibble() %>%
  dplyr::count(PSM.Ambiguity != "Unambiguous")

## # A tibble: 1 x 2
##   ‘PSM.Ambiguity != "Unambiguous"‘     n
##   <lgl>                            <int>
## 1 FALSE                            43153

## No PSMs to remove so proceed


### Assessing the impact of non-specific data cleaning

Now that we have finished the non-specific data cleaning, we can pause and check to see what this has done to the data. We determine the number and proportion of PSMs, peptides, and proteins lost from the original dataset.

## Determine number and proportion of PSMs removed
psms_remaining <- cp_qf[["psms_filtered"]] %>%
  nrow() %>%
  as.numeric()

psms_removed <- original_psms - psms_remaining
psms_removed_prop <- ((psms_removed / original_psms) * 100) %>%
  round(digits = 2)

## Determine number and proportion of peptides removed
peps_remaining <- cp_qf[["psms_filtered"]] %>%
  rowData() %>%
  as_tibble() %>%
  pull(Sequence) %>%
  unique() %>%
  length() %>%
  as.numeric()

peps_removed <- original_peps - peps_remaining
peps_removed_prop <- ((peps_removed / original_peps) * 100) %>%
  round(digits = 2)

## Determine number and proportion of proteins removed
prots_remaining <- cp_qf[["psms_filtered"]] %>%
  rowData() %>%
  as_tibble() %>%
  pull(Master.Protein.Accessions) %>%
  unique() %>%
  length() %>%
  as.numeric()

prots_removed <- original_prots - prots_remaining
prots_removed_prop <- ((prots_removed / original_prots) * 100) %>%
  round(digits = 2)

## Print as a table
data.frame("Feature" = c("PSMs",
                         "Peptides",
                         "Proteins"),
           "Number lost" = c(psms_removed,
             peps_removed,
             prots_removed),
           "Percentage lost" = c(psms_removed_prop,
                peps_removed_prop,
                prots_removed_prop))

##    Feature Number.lost Percentage.lost
## 1     PSMs        5679           11.63
## 2 Peptides        1565            6.03
## 3 Proteins         452            8.97


### PSM quality control filtering

The next step is to take a look at the data and make informed decisions about in-depth filtering. Here, we focus on three key quality control filters for TMT data: 1) average reporter ion signal-to-noise (S/N) ratio, 2) percentage co-isolation interference, and 3) percentage SPS mass match. It is possible to set thresholds for these three parameters during the identification search. However, specifying thresholds prior to exploring the data could lead to unnecessarily excessive data exclusion or the retention of poor quality PSMs. We suggest that users set the thresholds for all three aforementioned filters to 0 during the identification search, thus allowing maximum flexibility during data processing. In all cases, quality control filtering represents a trade-off between ensuring high quality data and losing potentially informative data. This means that the thresholds used for such filtering will likely depend upon the initial quality of the data and the number of PSMs, as well as the experimental goal being stringent or exploratory.

### Quality control: Average reporter ion signal-to-noise

Intensity measurements derived from a small number of ions tend to be more variable and less accurate. Therefore, reporter ion spectra with peaks generated from a small number of ions should be filtered out to ensure accurate quantitation and avoid stochastic ion effects. When using an orbitrap analyser, as was the case in the collection of the use-case data, the number of ions is proportional to the S/N value of a peak. Hence, the average reporter ion S/N ratio can be used to filter out quantification based on too few ions.

To determine an appropriate reporter ion S/N threshold we need to understand the original, unfiltered data. Here, we print a summary of the average reporter S/N before plotting a simple histogram to visualise the data. The default threshold for average reporter ion S/N when filtering within Proteome Discoverer is 10, or 1 on the base-10 logarithmic scale displayed here. We include a line to show where this threshold would be on the data distribution.

## Get summary information
cp_qf[["psms_filtered"]] %>%
  rowData() %>%
  as_tibble() %>%
  pull(Average.Reporter.SN) %>%
  summary()

##   Min.   1st Qu.   Median   Mean   3rd Qu.     Max.   NA’s
##    0.3      84.2    215.8  321.8     450.3   3008.2    140

## Plot histogram of reporter ion signal-to-noise
cp_qf[["psms_filtered"]] %>%
  rowData() %>%
  as_tibble() %>%
  ggplot(aes(x = log10(Average.Reporter.SN))) +
  geom_histogram(binwidth = 0.05) +
  geom_vline(xintercept = 1, linetype = "dashed", color = "red") +
  labs(x = "log10(average reporter SN)", y = "Frequency") +
  ggtitle("Average reporter ion S/N") +
  theme_bw()

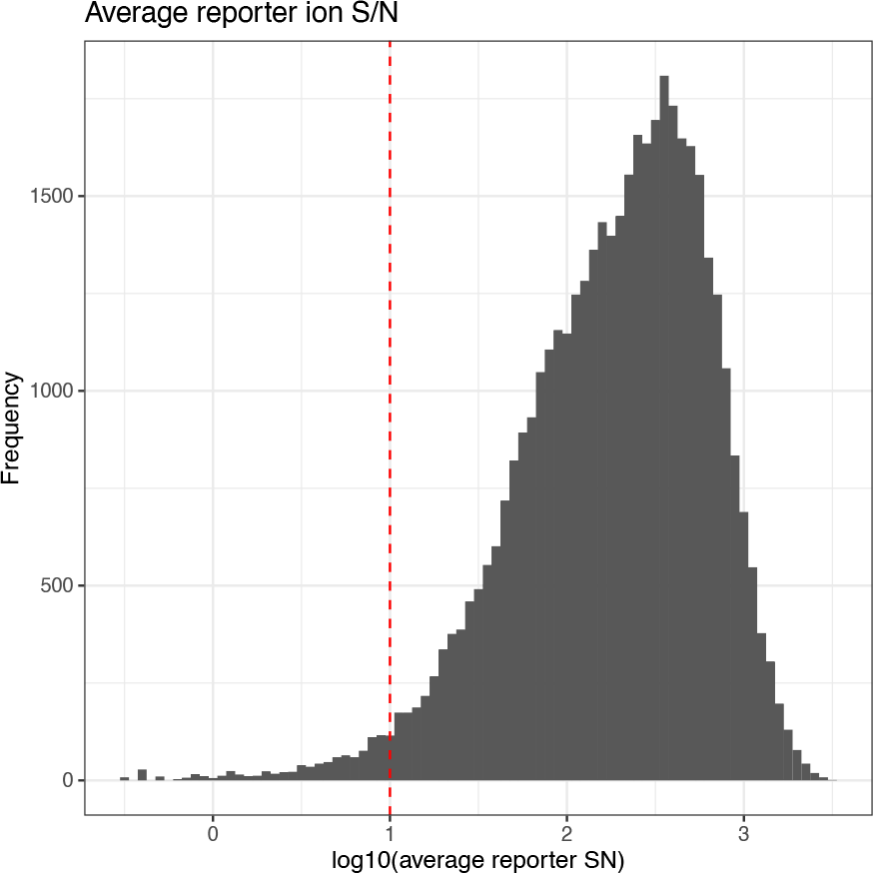


From the distribution of the data it is clear that applying such a threshold would not result in dramatic data loss. Whilst we could set a higher threshold for more stringent analysis, this would lead to unnecessary data loss. Therefore, we keep PSMs with an average reporter ion S/N threshold of 10 or more. We also remove PSMs that have an NA value for their average reporter ion S/N since their quality cannot be guaranteed. This is done by including na.rm = TRUE.

## Find out how many PSMs we expect to lose
cp_qf[["psms_filtered"]] %>%
  rowData() %>%
  as_tibble() %>%
  dplyr::count(Average.Reporter.SN < 10)

## # A tibble: 3 x 2
##   ‘Average.Reporter.SN < 10‘     n
##   <lgl>                      <int>
## 1 FALSE                      42066
## 2 TRUE                         947
## 3 NA                           140

## Drop these rows from the data
cp_qf <- cp_qf %>%
  filterFeatures(~ Average.Reporter.SN >= 10,
                 na.rm = TRUE,
                 i = "psms_filtered")


### Quality control: Isolation interference

A second data-dependent quality control parameter which should be considered is the isolation interference. The first type of interference that occurs during a TMT experiment is reporter ion interference, also known as cross-label isotopic impurity. This type of interference arises from manufacturing-level impurities and experimental error. The former should be reduced somewhat by the inclusion of lot-specific correction factors in the search set-up and users should ensure that these corrections are applied. In Proteome Discoverer this means setting “Apply Quan Value Corrections” to “TRUE” within the reporter ions quantifier node. The second form of interference is co-isolation interference which occurs during the MS run when multiple labelled precursor peptides are co-isolated in a single data acquisition window. Following fragmentation of the co-isolated peptides, this results in an MS2 or MS3 reporter ion peak (depending upon the experimental design) derived from multiple precursor peptides. Hence, co-isolation interference leads to inaccurate quantitation of the identified peptide. This problem is reduced by filtering out PSMs with a high percentage isolation interference value. As was the case for reporter ion S/N, Proteome Discoverer has a suggested default threshold for isolation interference - 50% for MS2 experiments and 75% for SPS-MS3 experiments.

Again, we get a summary and visualise the data using the code chunk below.

## Get summary information
cp_qf[["psms_filtered"]] %>%
  rowData() %>%
  as_tibble() %>%
  pull(Isolation.Interference.in.Percent) %>%
  summary()

##   Min.  1st Qu.   Median    Mean  3rd Qu.    Max.
##  0.000    0.000    8.385  12.637  21.053   84.379

## Plot histogram of co-isolation interference
cp_qf[["psms_filtered"]] %>%
  rowData() %>%
  as_tibble() %>%
  ggplot(aes(x = Isolation.Interference.in.Percent)) +
  geom_histogram(binwidth = 2) +
  geom_vline(xintercept = 75, linetype = "dashed", color = "red") +
  labs(x = "Isolation inteference (%)", y = "Frequency") +
  ggtitle("Co-isolation interference %") +
  theme_bw()

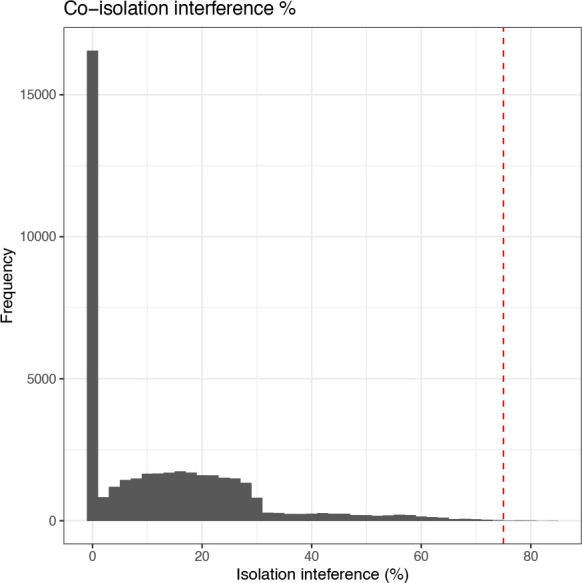


Looking at the data, very few PSMs have an isolation interference above the suggested threshold, and hence minimal data will be lost. Again, we choose to apply the standard threshold with the understanding that decreasing the threshold would result in greater data loss. Importantly, we are able to apply relatively standard thresholds here as the preliminary exploration did not expose any problems with the experimental data (in terms of labelling or MS analysis). If users have reason to believe the data is of poorer quality then more stringent thresholding should be considered.

## Find out how many PSMs we expect to lose
cp_qf[["psms_filtered"]] %>%
  rowData() %>%
  as_tibble() %>%
  dplyr::count(Isolation.Interference.in.Percent > 75)

## # A tibble: 2 x 2
##   ‘Isolation.Interference.in.Percent > 75‘      n
##   <lgl>                                     <int>
## 1 FALSE                                     42007
## 2 TRUE                                         59

## Remove these rows from the data
cp_qf <- cp_qf %>%
  filterFeatures(~ Isolation.Interference.in.Percent <= 75,
                 na.rm = TRUE,
                 i = "psms_filtered")


### Quality control: SPS mass match

The final quality control filter that we will apply is a percentage SPS mass match threshold. SPS mass match is a metric which has been introduced by Proteome Discoverer versions 2.3 and above to quantify the percentage of SPS-MS3 fragments that can still be explicitly traced back to the precursor peptide. This parameter is of particular importance given that quantitation is based on the SPS-MS3 spectra. Unfortunately, the SPS Mass Match percentage is currently only a feature of Proteome Discoverer (2.3 and above) and will not be available to users of other third party software.

We follow the same format as before to investigate the SPS Mass Match (%) distribution of the data. The default threshold within Proteome Discoverer is a SPS Mass Match above 65%. In reality, since SPS Mass Match is only reported to the nearest 10%, removing PSMs annotated with a value below 65% means removing those with 60% or less. Hence, only PSMs with 70% SPS Mass Match or above would be retained. We can see how many PSMs would be lost based on such thresholds using the code chunk below.

## Get summary information
cp_qf[["psms_filtered"]] %>%
  rowData() %>%
  as_tibble() %>%
  pull(SPS.Mass.Matches.in.Percent) %>%
  summary()

##   Min.  1st Qu.   Median   Mean  3rd Qu.    Max.
##   0.00    50.00    70.00  64.31   80.00   100.00

## Plot histogram of SPS mass match %
cp_qf[["psms_filtered"]] %>%
  rowData() %>%
  as_tibble() %>%
  ggplot(aes(x = SPS.Mass.Matches.in.Percent)) +
  geom_histogram(binwidth = 10) +
  geom_vline(xintercept = 65, linetype = "dashed", color = "red") +
  labs(x = "SPS mass matches (%)", y = "Frequency") +
  scale_x_continuous(breaks = seq(0, 100, 10)) +
  ggtitle("SPS mass match %") +
  theme_bw()

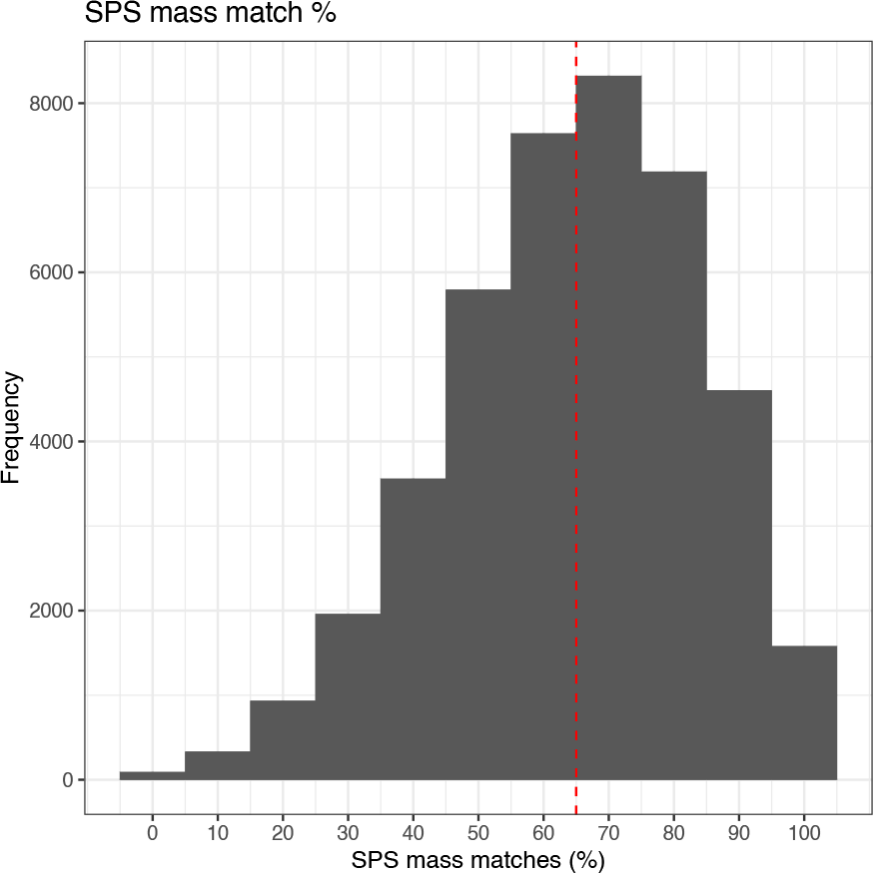


From the summary and histogram we can see that the distribution of SPS Mass Matches is much less skewed than that of average reporter ion S/N or isolation interference. This means that whilst the application of thresholds on average reporter ion S/N and isolation interference led to minimal data loss, attempting to impose a threshold on SPS Mass Match represents a much greater trade-off between data quality and quantity. For simplicity, here we choose to use the standard threshold of 65%.

## Find out how many PSMs we expect to lose
cp_qf[["psms_filtered"]] %>%
  rowData() %>%
  as_tibble() %>%
  dplyr::count(SPS.Mass.Matches.in.Percent < 65)

## # A tibble: 2 x 2
##   ‘SPS.Mass.Matches.in.Percent < 65‘      n
##   <lgl>                               <int>
## 1 FALSE                               21697
## 2 TRUE                                20310

## Drop these rows from the data
cp_qf <- cp_qf %>%
  filterFeatures(~ SPS.Mass.Matches.in.Percent >= 65,
                 na.rm = TRUE,
                 i = "psms_filtered")


### Assessing the impact of data-specific filtering

As we did after the non-specific cleaning steps, we check to see how many PSMs, peptides and proteins have been removed throughout the in-depth data-specific filtering.

## Summarize the effect of data-specific filtering

## Determine the number and proportion of PSMs removed
psms_remaining_2 <- cp_qf[["psms_filtered"]] %>%
  nrow() %>%
  as.numeric()

psms_removed_2 <- psms_remaining - psms_remaining_2
psms_removed_prop_2 <- ((psms_removed_2 / original_psms) * 100) %>%
  round(digits = 2)

## Determine number and proportion of peptides removed
peps_remaining_2 <- rowData(cp_qf[["psms_filtered"]])$Sequence %>%
  unique() %>%
  length() %>%
  as.numeric()

peps_removed_2 <- peps_remaining - peps_remaining_2
peps_removed_prop_2 <- ((peps_removed_2 / original_peps) * 100) %>%
  round(digits = 2)

## Determine number and proportion of proteins removed
prots_remaining_2 <- cp_qf[["psms_filtered"]] %>%
  rowData() %>%
  as_tibble() %>%
  pull(Master.Protein.Accessions) %>%
  unique() %>%
  length() %>%
  as.numeric()

prots_removed_2 <- prots_remaining - prots_remaining_2
prots_removed_prop_2 <- ((prots_removed_2 / original_prots) * 100) %>%
  round(digits = 2)

## Print as a table
data.frame("Feature" = c("PSMs",
                         "Peptides",
                         "Proteins"),
           "Number lost" = c(psms_removed_2,
                             peps_removed_2,
                             prots_removed_2),
           "Percentage lost" = c(psms_removed_prop_2,
                                 peps_removed_prop_2,
                                 prots_removed_prop_2))

##    Feature Number.lost Percentage.lost
## 1     PSMs       21456           43.94
## 2 Peptides       10162           39.13
## 3 Proteins        1299           25.77


### Managing missing data

Having finished the data cleaning at the PSM-level, the final step is to deal with missing data. Missing values represent a common challenge in quantitative proteomics and there is no consensus within the literature on how this challenge should be addressed. Indeed, missing values fall into different categories based on the reason they were generated, and each category is best dealt with in a different way. There are three main categories of missing data: missing completely at random (MCAR), missing at random (MAR) and missing not at random (MNAR). Within proteomics, values which are MCAR arise due to technical variation or stochastic fluctuations and emerge in a uniform, intensity-independent distribution. Examples include values for peptides which cannot be consistently identified or are unable to be efficiently ionised. By contrast, MNAR values are expected to occur in an intensity-dependent manner due to the presence of peptides at abundances below the limit of detection. ^17^^,^^22^^,^^23^ In many cases this is due to the biological condition being evaluated, for example the cell type or treatment applied.

To simplify this process, we consider the management of missing data in three steps. The first step is to determine the presence and pattern of missing values within the data. Next, we filter out data which exceed the desired proportion of missing values. This includes removing PSMs with a greater number of missing values across samples than we deem acceptable, as well as whole samples in cases where the proportion of missing values is substantially higher than the average. Finally, imputation can be used to replace any remaining NA values within the dataset. This final step is optional and can equally be done prior to filtering if the user wishes to impute all missing values without removing any PSMs, although this is not recommended. Further, whilst it is possible to complete such steps at the peptide- or protein-level, we advise management of missing values at the lowest data level to minimise the effect of implicit imputation during aggregation.

### Exploring the presence of missing values

First, to determine the presence of missing values in the PSM-level data we use the nNA function within the QFeatures infrastructure. This function will return the absolute number and percentage of missing values both per sample and as an average. Importantly, alternative third-party software may output missing values in formats other than NA, such as zero, or infinite. In such cases, missing values can be converted directly into NA values through use of the zeroIsNA or infIsNA functions within the QFeatures infrastructure.

## Determine whether there are any NA values in the data
cp_qf[["psms_filtered"]] %>%
  assay() %>%
  anyNA()

## [1] TRUE

## Determine the amount and distribution of NA values in the data
cp_qf[["psms_filtered"]] %>%
  nNA()

## $nNA
## DataFrame with 1 row and 2 columns

##         nNA        pNA
##   <integer>  <numeric>
## 1         4 0.00307262
##
## $nNArows
## DataFrame with 21697 rows and 3 columns
##              name       nNA       pNA
##       <character> <integer> <numeric>
## 1              13         0         0
## 2              20         0         0
## 3              25         0         0
## 4              26         0         0
## 5              29         0         0
## ...           ...       ...       ...
## 21693       48786         0         0
## 21694       48792         0         0
## 21695       48797         0         0
## 21696       48810         0         0
## 21697       48819         0         0
##
## $nNAcols
## DataFrame with 6 rows and 3 columns
##          name       nNA        pNA
##   <character> <integer>  <numeric>
## 1          S1         0 0.00000000
## 2          S2         2 0.00921786
## 3          S3         0 0.00000000
## 4          S4         1 0.00460893
## 5          S5         1 0.00460893
## 6          S6         0 0.00000000


We can see that the data only contains 0.003% missing values, corresponding to 4 NA values. This low proportion is due to a combination of the TMT labelling strategy and the stringent PSM quality control filtering. In particular, co-isolation interference when using TMT labels often results in very low quantification values for peptides which should actually be missing or ‘NA’. Nevertheless, we continue and check for sample-specific bias in the distribution of NAs by plotting a simple histogram. We also use colour to indicate the condition of each sample as to check for condition-specific bias.

## Plot histogram to visualize the distribution of NAs
nNA(cp_qf[["psms_filtered"]])$nNAcols %>%
  as_tibble() %>%
  mutate(Condition = rep(c("Treated", "Control"), each = 3)) %>%
  ggplot(aes(x = name, y = pNA, group = Condition, fill = Condition)) +
  geom_bar(stat = "identity", position = "dodge") +
  geom_hline(yintercept = 0.002, linetype = "dashed", color = "red") +
  labs(x = "Sample", y = "Missing values (%)") +
  theme_bw()

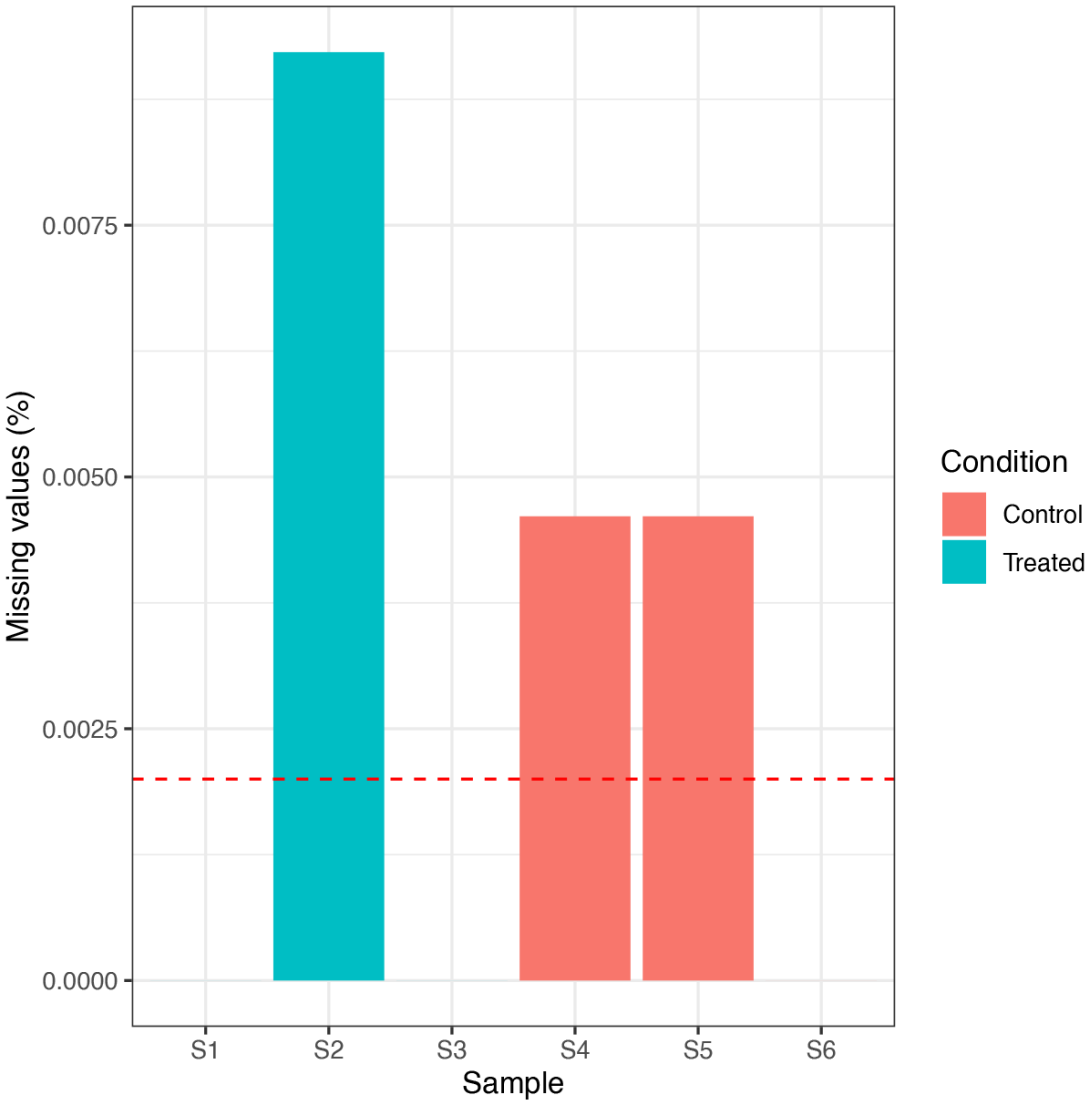


The percentage of missing values is sufficiently low that none of the samples need be removed. Further, there is no sample- or condition-specific bias in the data. We can get more information about the PSMs with NA values using the code below.

## Find out the range of missing values per PSM
nNA(cp_qf[["psms_filtered"]])$nNArows$nNA %>%
  table()

## .
##     0   1
## 21693   4


From this output we can see that the maximum number of NA values per PSM is one. This information is useful to know as it may inform the filtering strategy in the next step.

## Get indices of rows which contain NA
rows_with_na_indices <- which(nNA(cp_qf[["psms_filtered"]])$nNArows$nNA != 0)

## Subset rows with NA
rows_with_na <- cp_qf[["psms_filtered"]][rows_with_na_indices, ]

## Inspect rows with NA
assay(rows_with_na)

##         S1   S2   S3   S4   S5   S6
## 12087 11.0 17.0 13.3 22.1   NA 30.6
## 30824 45.0   NA 43.1 66.7 69.7 62.1
## 30846 34.3   NA 47.9 56.8 65.5 57.2
## 44791 22.8 28.7 19.6   NA  3.8 12.2


### Filtering out missing values

First we apply some standard filtering. Typically, it is desirable to remove features, here PSMs, with greater than 20% missing values. We can do this using the filterNA function in QFeatures, as outlined below. We pass the function the SummarizedExperiment and use the pNA = argument to specify the maximum proportion of NA values to allow.

## Check how many PSMs we will remove
nNA(cp_qf[["psms_filtered"]])$nNArows %>%
  as_tibble() %>%
  dplyr::count(pNA >= 20)

## # A tibble: 1 x 2
##   ‘pNA >= 20‘     n
##   <lgl>       <int>
## 1 FALSE       21697


Although the use-case data does not contain any PSMs with >20% missing values, we demonstrate how to apply the desired filter below.

## Remove PSMs with more than 20 % (0.2) NA values
cp_qf <- cp_qf %>%
  filterNA(pNA = 0.2,
           i = "psms_filtered")


Since previous exploration of missing data did not reveal any sample with an excessive number of NA values, we do not need to remove any samples from the analysis.

Although not covered here, users may wish to carry out condition-specific filtering in cases where the exploration of missing values revealed a condition- specific bias, or where the experimental question requires. This would be the case, for example, if one condition was transfected to express proteins of interest whilst the control condition lacked these proteins. Filtering of both conditions together could, therefore, lead to the removal of proteins of interest.

### Imputation (optional)

The final step is to consider whether to impute the remaining missing values within the data. Imputation refers to the replacement of missing values with probable values. Since imputation requires complex assumptions and can have substantial effects on downstream statistical analysis, we here choose to skip imputation. This is reasonable given that we only have 3 missing values at the PSM-level, and that some of these will likely be removed by aggregation. A more in-depth discussion of imputation will be provided below in the LFQ workflow.

### Summary of PSM data cleaning

Thus far we have checked that the experimental data we are using is of high quality by visualising the raw data and calculating TMT labelling efficiency. We then carried out non-specific data cleaning, data-specific filtering steps and management of missing data. Here, we present a combined summary of these PSM processing steps.

## Determine final number of PSMs, peptides and master proteins
psms_final <- cp_qf[["psms_filtered"]] %>%
  nrow() %>%
  as.numeric()

psms_removed_total <- original_psms - psms_final
psms_removed_total_prop <- ((psms_removed_total / original_psms) * 100) %>%
  round(digits = 2)

peps_final <- cp_qf[["psms_filtered"]] %>%
  rowData() %>%
  as_tibble() %>%
  pull(Sequence) %>%
  unique() %>%
  length() %>%
  as.numeric()

peps_removed_total <- original_peps - peps_final
peps_removed_total_prop <- ((peps_removed_total / original_peps) * 100) %>%
  round(digits = 2)

prots_final <- cp_qf[["psms_filtered"]] %>%
  rowData() %>%
  as_tibble() %>%
  pull(Master.Protein.Accessions) %>%
  unique() %>%
  length() %>%
  as.numeric()

prots_removed_total <- original_prots - prots_final
prots_removed_total_prop <- ((prots_removed_total / original_prots) * 100) %>%
  round(digits = 2)

## Print as table
data.frame("Feature" = c("PSMs",
                         "Peptides",
                         "Proteins"),
           "Number lost" = c(psms_removed_total,
                             peps_removed_total,
                             prots_removed_total),
           "Percentage lost" = c(psms_removed_total_prop,
                                 peps_removed_total_prop,
                                 prots_removed_total_prop),
           "Number remaining" = c(psms_final,
                                  peps_final,
                                  prots_final))

##    Feature Number.lost Percentage.lost Number.remaining
## 1     PSMs       27135           55.57            21697
## 2 Peptides       11727           45.16            14242
## 3 Proteins        1751           34.74             3289


### Logarithmic transformation of quantitative data

Once satisfied that the PSM-level data is clean and of high quality, the PSM-level quantitative data is log transformed. log2 transformation is a standard step when dealing with quantitative proteomics data since protein abundances are dramatically skewed towards zero. Such a skewed distribution is to be expected given that the majority of cellular proteins present at any one time are of relatively low abundance, whilst only a few highly abundant proteins exist. To perform the logarithmic transformation and generate normally distributed data we pass the PSM-level data in the QFeatures object to the logTransform function, as per the below code chunk.

## log2 transform quantitative data
cp_qf <- logTransform(object = cp_qf,
                      base = 2,
                      i = "psms_filtered",
                      name = "log_psms")
## Verify
cp_qf

## An instance of class QFeatures containing 3 assays:
##  [1] psms_raw: SummarizedExperiment with 48832 rows and 6 columns
##  [2] psms_filtered: SummarizedExperiment with 21697 rows and 6 columns
##  [3] log_psms: SummarizedExperiment with 21697 rows and 6 columns


### Aggregation of PSMs to proteins

For the aggregation itself we use the aggregateFeatures function and provide the base level from which we wish to aggregate, the log PSM-level data in this case. We also tell the function which column to aggregate, which is specified by the fcol argument. We will first aggregate from PSM to peptide to create explicit QFeatures links. This means grouping by PSM “Sequence”.

As well as grouping PSMs according to their peptide sequence, the quantitative values for each PSM must be aggregated into a single peptide-level value. The default aggregation method within aggregateFeatures is the robustSummary function from the MsCoreUtils package. ^19^ This method is a form of robust regression and is described in detail elsewhere. ^24^ Nevertheless, the user must decide which aggregation method is most appropriate for their data and biological question. Further, an understanding of the selected method is critical given that aggregation is a form of implicit imputation and has substantial effects on the downstream data. Indeed, aggregation methods have different ways of dealing with missing data, either by removal or propagation. Options of aggregation methods within the aggregateFeatures function include MsCoreUtils::medianPolish , MsCoreUtils::robustSummary , base::colMeans , base::colSums , and matrixStats::colMedians . Users should also be aware that some methods have specific input requirements. For example, robustSummary assumes that intensities have already been log transformed.

### Aggregating using robust summarisation

Here, we use robustSummary to aggregate from PSM to peptide-level. This method is currently considered to be state-of-the-art as it is more robust against outliers than other aggregation methods. ^24^^,^^25^ We also include na.rm = TRUE to exclude any NA values prior to completing the summarisation.

## Aggregate PSM to peptide
cp_qf <- aggregateFeatures(cp_qf,
                           i = "log_psms",
                           fcol = "Sequence",
                           name = "log_peptides",
                           fun = MsCoreUtils::robustSummary,
                           na.rm = TRUE)

## Your quantitative and row data contain missing values. Please read the
## relevant section(s) in the aggregateFeatures manual page regarding the
## effects of missing values on data aggregation.

## Verify
cp_qf

## An instance of class QFeatures containing 4 assays:
##  [1] psms_raw: SummarizedExperiment with 48832 rows and 6 columns
##  [2] psms_filtered: SummarizedExperiment with 21697 rows and 6 columns
##  [3] log_psms: SummarizedExperiment with 21697 rows and 6 columns
##  [4] log_peptides: SummarizedExperiment with 14242 rows and 6 columns


We are now left with a QFeatures object holding the PSM and peptide-level data in their own SummarizedExperiments. Importantly, an explicit link has been maintained between the two levels and this makes it possible to gain information about all PSMs that were aggregated into a peptide.

### Considerations for aggregating non-imputed data

If users did not impute prior to aggregation, NA values within the PSM-level data may have propagated into NaN values. This is because peptides only supported by PSMs containing missing values would not have any quantitative value to which a sum or median function, for example, can be applied. Therefore, we check for NaN and convert back to NA values to facilitate compatibility with downstream processing.

## Confirm the presence of NaN
assay(cp_qf[["log_peptides"]]) %>%
  is.nan() %>%
  table()

## .
## FALSE
## 85452

## Replace NaN with NA
assay(cp_qf[["log_peptides"]])[is.nan(assay(cp_qf[["log_peptides"]]))] <- NA


Next, using the same approach as above, we use the aggregateFeatures function to assemble the peptides into proteins. As before, we must pass several arguments to the function. Namely, the QFeatures object i.e. cp_qf, the data level we wish to aggregation from i.e. log_peptides, the column of the rowData defining how to aggregate the features i.e. by "Master.Protein.Accessions" and a name for the new data level e.g. "log_proteins". We again choose to use robustSummary as our aggregation method and we pass na.rm = TRUE to ignore NA values. Users can type ?aggregateFeatures to see more information. Users should be aware that peptides are grouped by their master protein accession and, therefore, downstream differential expression analysis will consider protein groups rather than individual proteins.

## Aggregate peptides to protein
cp_qf <- aggregateFeatures(cp_qf,
                           i = "log_peptides",
                           fcol = "Master.Protein.Accessions",
                           name = "log_proteins",
                           fun = MsCoreUtils::robustSummary,
                           na.rm = TRUE)

## Your quantitative and row data contain missing values. Please read the
## relevant section(s) in the aggregateFeatures manual page regarding the
## effects of missing values on data aggregation.

## Verify
cp_qf

## An instance of class QFeatures containing 5 assays:
##  [1] psms_raw: SummarizedExperiment with 48832 rows and 6 columns
##  [2] psms_filtered: SummarizedExperiment with 21697 rows and 6 columns
##  [3] log_psms: SummarizedExperiment with 21697 rows and 6 columns
##  [4] log_peptides: SummarizedExperiment with 14242 rows and 6 columns
##  [5] log_proteins: SummarizedExperiment with 3289 rows and 6 columns


Following aggregation, we have a total of 3289 proteins remaining within the data.

### Normalisation of quantitative data

After transforming the data, we normalise the protein-level abundances. Normalization is a process of correction whereby quantitative data is returned to its original, or ‘normal’, state. In expression proteomics, the aim of post-acquisition data normalization is to minimise the biases that arises due to experimental error and technological variation. Specifically, the removal of random variation and batch effects will allow samples to be aligned prior to downstream analysis. Importantly, however, users must also be aware of any normalization that has taken place within their sample preparation, as this will ultimately influence the presence of differentially abundant proteins downstream. An extensive review on normalization strategies, both experimental and computational, is provided in Ref. 26.

Unfortunately, there is not currently a single normalization method which performs best for all quantitative proteomics datasets. Within the Bioconductor packages, however, exists NormalyzerDE , a tool for evaluating different normalisation methods. ^27^ By passing a SummarizedExperiment object to the normalyzer function it is possible to generate a report comparing common normalisation strategies, such as total intensity (TI), median intensity (MedI), average intensity (AI), quantile (from the preprocessCore package), ^28^ NormFinder (NM), ^29^ Variance Stabilising Normalization (VSN, from the vsn package), ^30^ Robust Linear Regression (RLR), and LOESS (from the limma package). ^31^ A number of qualitative and quantitative evaluation measures are provided within the report, including total intensity, Pooled intragroup Coefficient of Variation (PCV), Pooled intragroup Median Absolute Deviation (PMDA), CV-intensity plots, MA-plots, Pearson and Spearman correlation.

Normalyzer accepts intensity data in a raw format, prior to log transformation. Therefore, we first generate a protein-level SummarizedExperiment from our PSM-level data prior to transformation.

## Aggregate from PSM directly to protein
cp_qf <- aggregateFeatures(cp_qf,
                           i = "psms_filtered",
                           fcol = "Master.Protein.Accessions",
                           name = "proteins_direct",
                           fun = MsCoreUtils::robustSummary,
                           na.rm = TRUE)


Hence, we will use the “proteins_direct” SummarizedExperiment here and the function will do the log2 transformation for us. A second important consideration is that missing values must be denoted ‘NA’, not zero, NaN or infinite. We can pass the SummarizedExperiment containing the protein data to the normalyzer function. With this, we provide a name for the report and the directory in which to save the report. The normalyzer function also expects two pieces of information, the sample name and corresponding experimental group. We previously annotated the data with this information through the sample and condition columns of the colData, so we tell the normalyzer function to look here.

## Generate normalyzer report
normalyzer(jobName = "normalyzer",
           experimentObj = cp_qf[["proteins_direct"]],
           sampleColName = "sample",
           groupColName = "condition",
           outputDir = ".")


**Figure 4.**
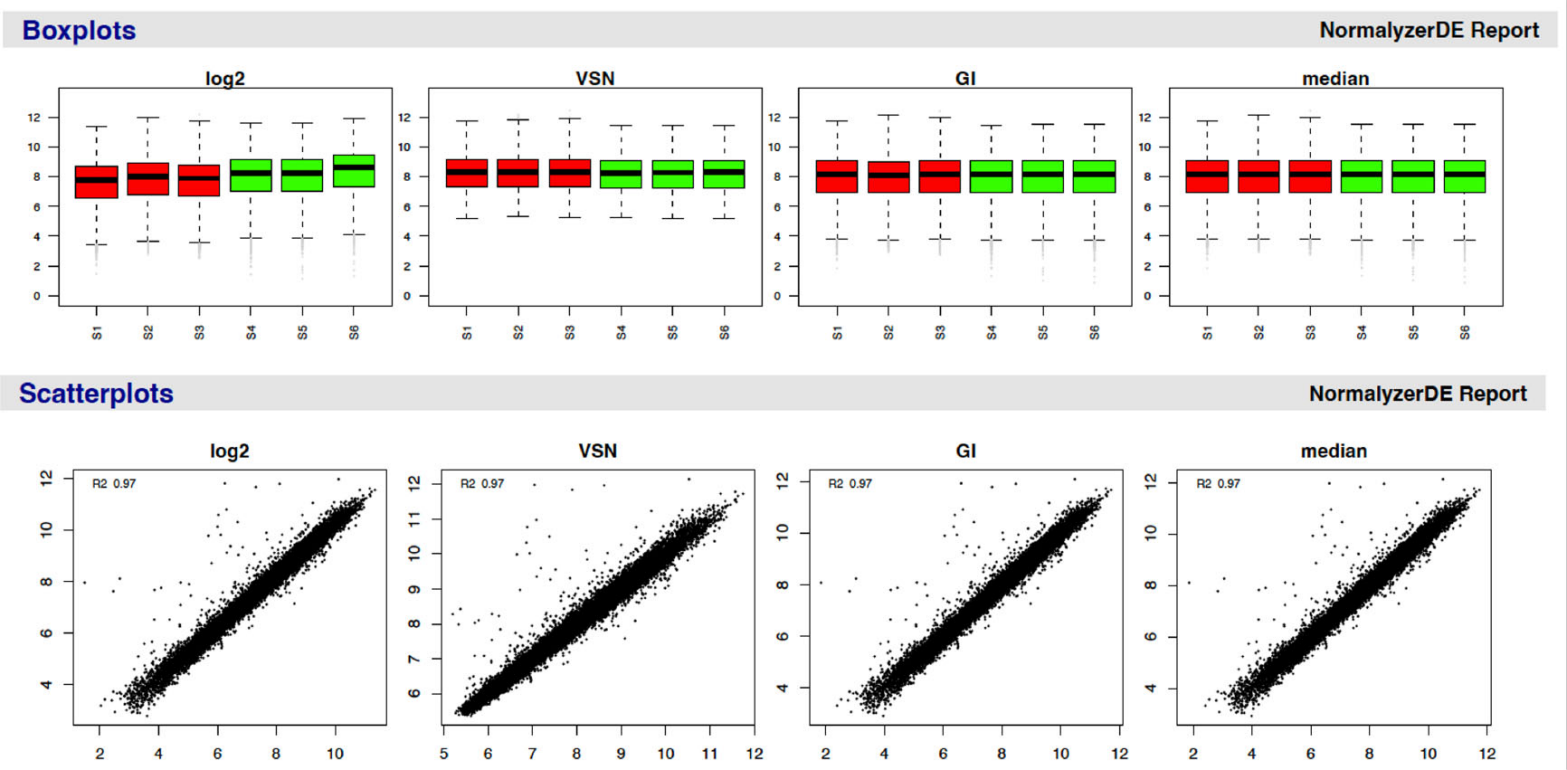
Example of plots generated by the normalyzer tool and provided in the .pdf report. Boxplots (top) and scatterplots (bottom) are two of the evaluation measures within the normalyzer report. Samples are grouped based on their condition to provide users with an easy way to evaluate the suitability of different normalization methods for their data. The log2 data can be used as a reference to compare the data pre- and post-normalization.

The function will take a few minutes to run, particularly if there are many samples. Once complete, the report can be accessed as a .pdf file containing plots such as those displayed in Figure 4.

Since the normalyzer report did not indicate any superior normalisation method in this case, we will apply a center median approach here. To do this, we pass the log transformed protein-level data to the normalize function in QFeatures. We specify the method of normalisation that we wish to apply i.e. method = "center.median" and name the new data level e.g. name = "log_norm_proteins". Of note, for users who wish to apply VSN normalisation the raw protein data must be passed (prior to any log transformation) as the log transformation is done internally when specify method = "vsn". All other methods require users to explicitly perform log transformation on their data before use. More details can be found in the QFeatures documentation, please type help("normalize,QFeatures-method").

## normalize the log transformed peptide data
cp_qf <- normalize(cp_qf,
                   i = "log_proteins",
                   name = "log_norm_proteins",
                   method = "center.median")

## Verify
cp_qf

## An instance of class QFeatures containing 7 assays:
##  [1] psms_raw: SummarizedExperiment with 48832 rows and 6 columns
##  [2] psms_filtered: SummarizedExperiment with 21697 rows and 6 columns
##  [3] log_psms: SummarizedExperiment with 21697 rows and 6 columns
##  [4] log_peptides: SummarizedExperiment with 14242 rows and 6 columns
##  [5] log_proteins: SummarizedExperiment with 3289 rows and 6 columns
##  [6] proteins_direct: SummarizedExperiment with 3289 rows and 6 columns
##  [7] log_norm_proteins: SummarizedExperiment with 3289 rows and 6 columns


To evaluate the effect of normalisation we plot a simple boxplot.

## Evaluate the effect of data normalization
pre_norm <- cp_qf[["log_proteins"]] %>%
  assay() %>%
  longFormat() %>%
  mutate(Condition = ifelse(colname %in% c("S1", "S2", "S3"),
                            "Treated", "Control")) %>%
  ggplot(aes(x = colname, y = value, fill = Condition)) +
  geom_boxplot() +
  labs(x = "Sample", y = "log2(abundance)", title = "Pre-normalization") +
  theme_bw()

post_norm <- cp_qf[["log_norm_proteins"]] %>%
  assay() %>%
  longFormat() %>%
  mutate(Condition = ifelse(colname %in% c("S1", "S2", "S3"),
                            "Treated", "Control")) %>%
  ggplot(aes(x = colname, y = value, fill = Condition)) +
  geom_boxplot() +
  labs(x = "Sample", y = "log2(abundance)", title = "Post-normalization") +
  theme_bw()

(pre_norm + theme(legend.position = "none")) +
  post_norm & plot_layout(guides = "collect")

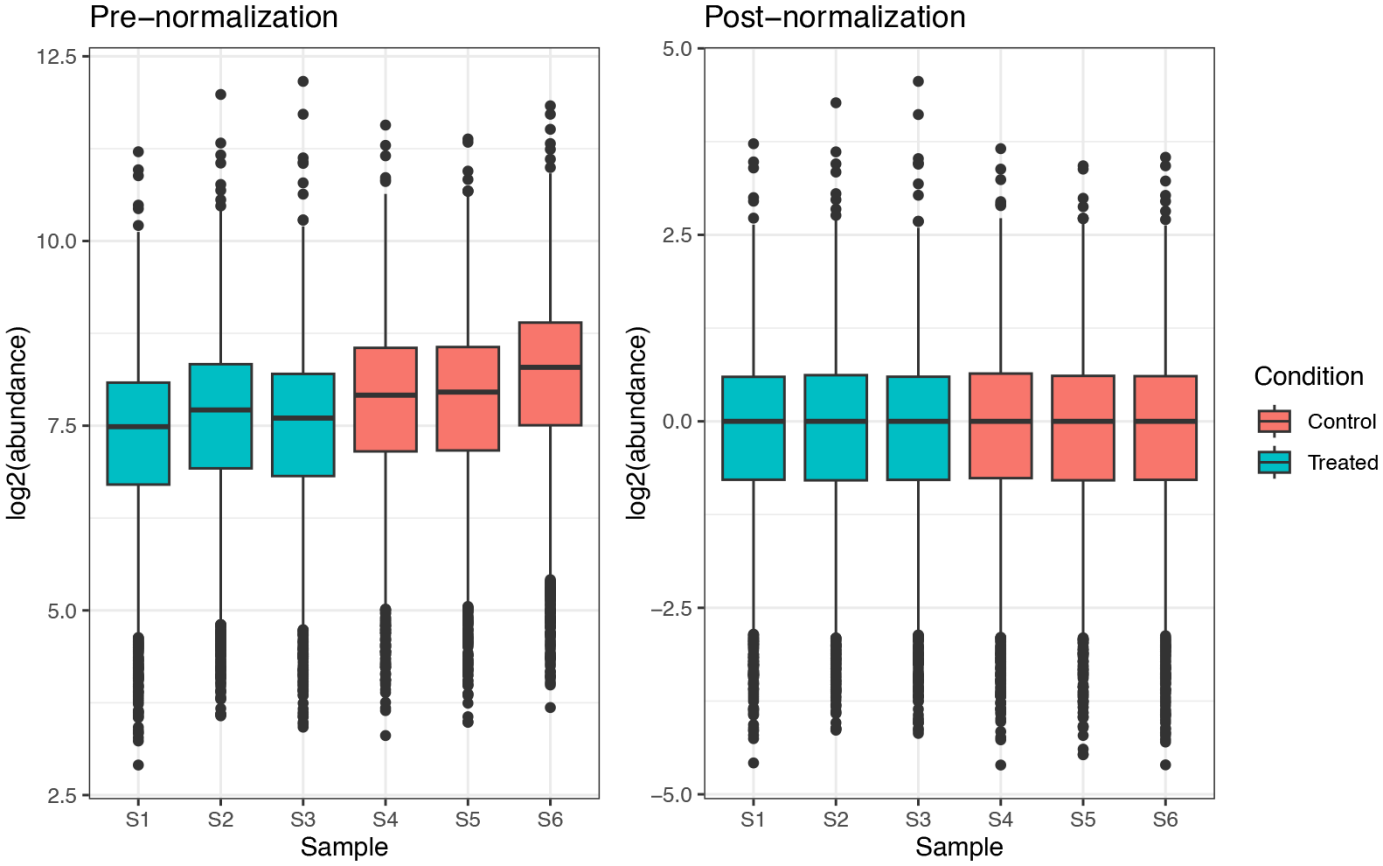


We can now generate a density plot to help us visualise what the process of log transformation and normalisation has done to the data. This is done using the plotDensities function from the limma package.

## visualize the process of log transformation and normalization
par(mfrow = c(1, 3))

cp_qf[["psms_filtered"]] %>%
  assay() %>%
  plotDensities(legend = "topright",
                main = "Raw PSMs")

cp_qf[["log_psms"]] %>%
  assay() %>%
  plotDensities(legend = FALSE,
                main = "log2(PSMs)")

cp_qf[["log_norm_proteins"]] %>%
  assay() %>%
  plotDensities(legend = FALSE,
                main = "log2(norm proteins)")

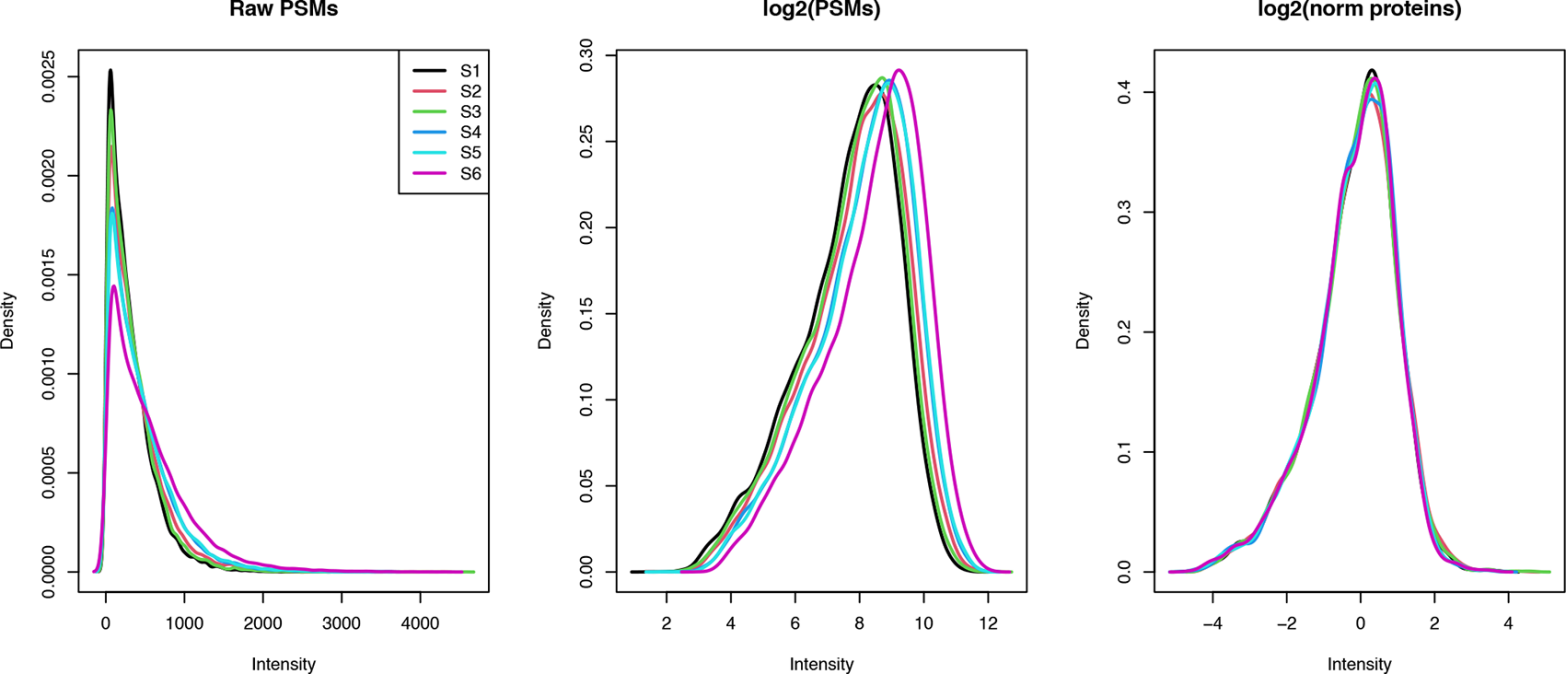


### Exploration of data using QFeatures links

### Creating assay links

After completing all data pre-processing, we now add explicit links between our final protein-level data and the raw PSM-level data which we created as an untouched copy. This allows us to investigate all data corresponding to the final proteins, including the data that has since been removed. To do this, we use the addAssayLinks function, demonstrated below. We can check that the assay links have been generated correctly by passing our QFeatures object to the AssayLink function along with the assay of interest ( i =).

## Add assay links from log_norm_proteins to psms_raw
cp_qf <- addAssayLink(object = cp_qf,
                      from = "psms_raw",
                      to = "log_norm_proteins",
                      varFrom = "Master.Protein.Accessions",
                      varTo = "Master.Protein.Accessions")

## Verify
assayLink(cp_qf,
          i = "log_norm_proteins")

## AssayLink for assay <log_norm_proteins>
## [from:psms_raw|fcol:Master.Protein.Accessions|hits:42678]


### Visualising aggregation

One of the characteristic attributes of the QFeatures infrastructure is that explicit links have been maintained throughout the aggregation process. This means that we can now access all data corresponding to a protein, its component peptides and PSMs. One way to do this is through the use of the subsetByFeature function which will return a new QFeatures object containing data for the desired feature across all levels. For example, if we wish to subset information about the protein “Q01581”, that is hydroxymethylglutaryl-CoA synthase, we could use the following code:

## Subset all data linked to the protein with accession Q01581
Q01581 <- subsetByFeature(cp_qf, "Q01581")

## Verify
Q01581

## An instance of class QFeatures containing 7 assays:
##  [1] psms_raw: SummarizedExperiment with 42 rows and 6 columns
##  [2] psms_filtered: SummarizedExperiment with 27 rows and 6 columns
##  [3] log_psms: SummarizedExperiment with 27 rows and 6 columns
##  [4] log_peptides: SummarizedExperiment with 15 rows and 6 columns
##  [5] log_proteins: SummarizedExperiment with 1 rows and 6 columns
##  [6] proteins_direct: SummarizedExperiment with 1 rows and 6 columns
##  [7] log_norm_proteins: SummarizedExperiment with 1 rows and 6 columns


We find that in this data the protein Q01581 has 15 peptides and 27 supporting its identification and quantitation. We also see that the original data prior to processing contained 42 PSMs in support of this protein.

Further, we can visualise the process of aggregation that has led to the protein-level abundance data for Q01581, as demonstrated below. Of note, this plot shows the protein data prior to normalisation.

## Define conditions
treament <- c("S1", "S2", "S3")
control <- c("S4", "S5", "S6")

## Plot abundance distributions across samples at PSM, peptide and protein-level
Q01581[, , c("log_psms", "log_peptides", "log_proteins")] %>%
  longFormat() %>%
  as_tibble() %>%
  mutate(assay_order = factor(
    assay,
    levels = c("log_psms", "log_peptides", "log_proteins"),
    labels = c("PSMs", "Peptides", "Protein")),
    condition = ifelse(colname %in% control, "control", "treatment")) %>%
  ggplot(aes(x = colname, y = value, colour = assay)) +
  geom_point(size = 3) +
  geom_line(aes(group = rowname)) +
  scale_x_discrete(limits = paste0("S", 1:6)) +
  facet_wrap(~assay_order) +
  labs(x = "Sample", y = "Abundance") +
  ggtitle("log2 Q01581 abundance profiles") +
  theme_bw()

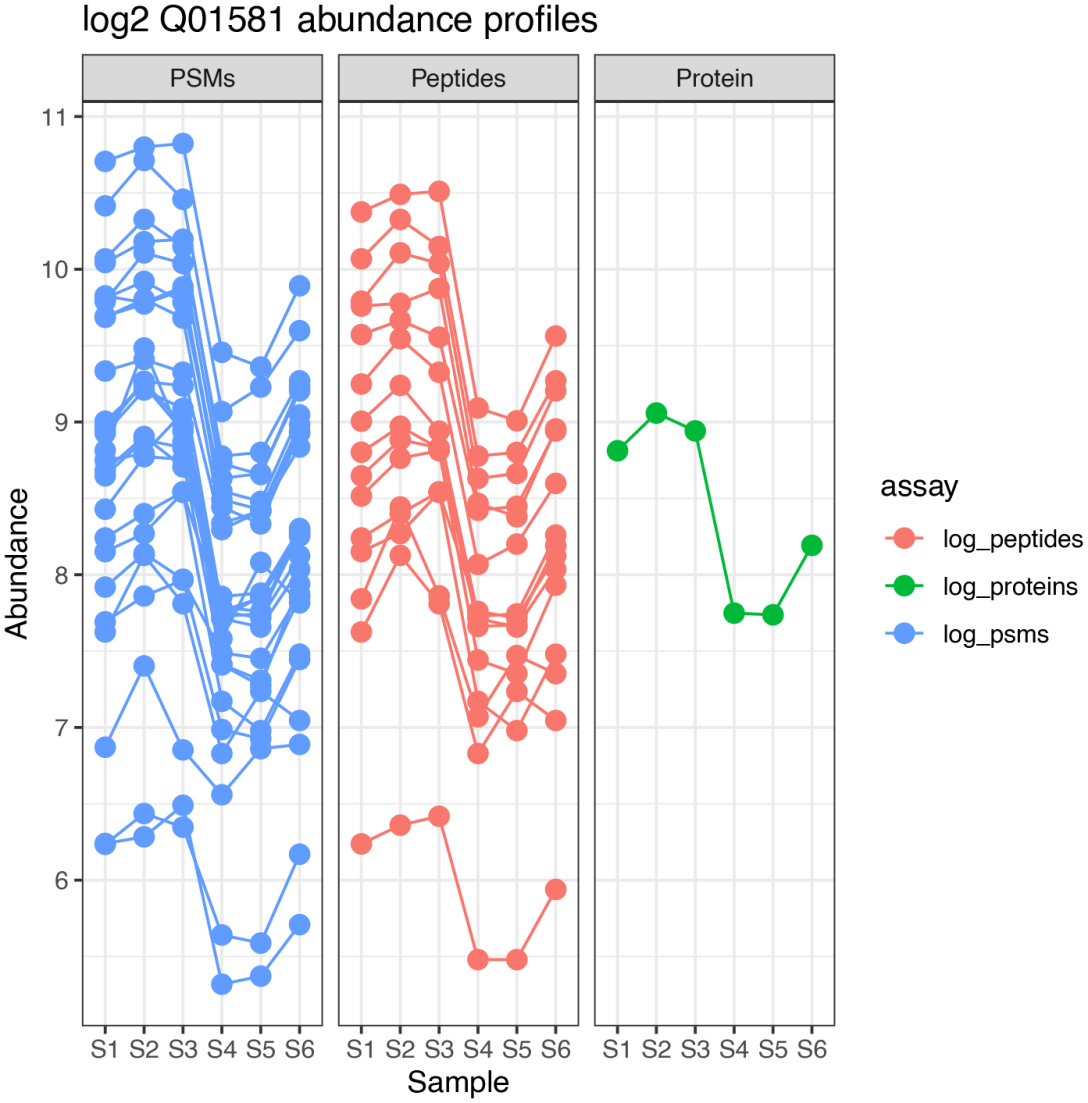


### Determining PSM and peptide support

Another benefit of the explicit links maintained within a QFeatures object is the ease at which we can determine PSM and peptide support per protein. When applying the aggregateFeatures function a column, termed ".n", is created within the rowData of the new SummarizedExperiment. This column indicates how many lower-level features were aggregated into each new higher-level feature. Hence, ".n" in the peptide-level data represents how many PSMs were aggregated into a peptide, whilst in the protein-level data it tells us how many peptides were grouped into a master protein. For ease of plotting, we will use the "proteins_direct" data generated above. Since this data was generated via direct aggregation of PSM to protein, ".n" this will tell us PSM support per protein. We plot these data as simple histograms.

## Plot PSM support per protein - .n in the proteins_direct SE
psm_per_protein <- cp_qf[["proteins_direct"]] %>%
  rowData() %>%
  as_tibble() %>%
  ggplot(aes(x = .n)) +
  geom_histogram(binwidth = 1, boundary = 0.5) +
  labs(x = "PSM support (shown up to 20)",
       y = "Frequency") +
  scale_x_continuous(expand = c(0, 0),
                     limits = c(0, 20.5),
                     breaks = seq(1, 20, 1)) +
  scale_y_continuous(expand = c(0, 0),
                     limits = c(0, 1000),
                     breaks = seq(0, 1000, 100)) +
  ggtitle("PSM support per protein") +
  theme_bw()

## Plot peptide support per protein - .n in the proteins SE
peptide_per_protein <- cp_qf[["log_proteins"]] %>%
  rowData() %>%
  as_tibble() %>%
  ggplot(aes(x = .n)) +
  geom_histogram(binwidth = 1, boundary = 0.5) +
  labs(x = "Peptide support (shown up to 20)",
       y = "Frequency") +
  scale_x_continuous(expand = c(0, 0),
                     limits = c(0, 20.5),
                     breaks = seq(1, 20, 1)) +
  scale_y_continuous(expand = c(0, 0),
                     limits = c(0, 1100),
                     breaks = seq(0, 1100, 100)) +
  ggtitle("Peptide support per protein") +
  theme_bw()

psm_per_protein + peptide_per_protein



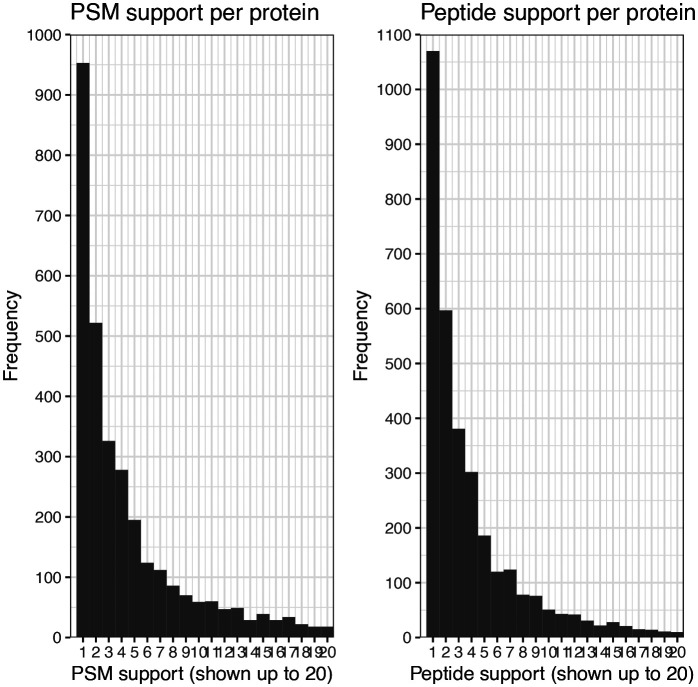


At this point, users may wish to include additional quality control filtering based on PSM and/or peptide support per protein. Given the extensive quality control filtering already applied in this workflow, we decide not to remove additional proteins based on PSM or peptide support.

### Data export

Finally, we save the protein-level data and export the QFeatures object into an .rda file so that we can re-load it later at convenience.

## Save protein-level SE
cp_proteins <- cp_qf[["log_norm_proteins"]]

## Export the final TMT QFeatures object
save(cp_qf, file = "cp_qf.rda")


## Label-free data processing workflow

Having discussed the processing of quantitative TMT-labelled data, we now move on to consider that of label-free quantitative (LFQ) data. As described previously, the cell culture supernatant fractions of triplicate control and treated HEK293 cells were kept label-free. As such, each sample was analysed using an independent mass spectrometry run without pre-fractionation. Again, a two-hour gradient in an Orbitrap Lumos Tribrid mass spectrometer coupled to an UltiMate 3000 HPLC system was applied. Given that much of the TMT pre-processing workflow also applies to label-free data, we only discuss steps which are different to those previously described. Readers are advised to refer to the TMT processing workflow for a more in-depth explanation of any shared steps.

### Identification search using Proteome Discoverer

As was the case for TMT labelled cell pellets, raw LFQ data from supernatant samples was searched using Proteome Discoverer 2.5. The GitHub repository associated with this manuscript can be found at https://github.com/CambridgeCentreForProteomics/f1000_expression_proteomics which contains the identification search along with an additional explanation of key parameters in an appendix. To begin processing LFQ data, users should export a peptide-level .txt file from the results of their identification search.

### Data import, housekeeping and exploration

Unlike the TMT-labelled use-case data which was processed from the PSM-level, the label-free use-case data can only be considered from the peptide-level up. This is because a retention time alignment algorithm (equivalent to match between runs) was applied to the PSM-level data. This means that peptides can be identified in samples even without a corresponding PSM, simply by sharing feature information across runs.

### Importing data into a QFeatures object

We locate the PeptideGroups .txt file and upload this into a QFeatures data container in the same way as before. Since the samples are already stored in the correct order, we simply identify the quantitative columns by their indices.

## Locate the PeptideGroups .txt file
sn_peptide <- "supernatant_lfq_results_peptides.txt"

## Identify columns containing quantitative data
sn_peptide %>%
  read.delim() %>%
  names()

##  [1] "Peptide.Groups.Peptide.Group.ID"
##  [2] "Checked"
##  [3] "Tags"
##  [4] "Confidence"
##  [5] "PSM.Ambiguity"
##  [6] "Sequence"
##  [7] "Modifications"
##  [8] "Modifications.all.possible.sites"
##  [9] "Qvality.PEP"
## [10] "Qvality.q.value"
## [11] "SVM_Score"
## [12] "Number.of.Protein.Groups"
## [13] "Number.of.Proteins"
## [14] "Number.of.PSMs"
## [15] "Master.Protein.Accessions"
## [16] "Master.Protein.Descriptions"
## [17] "Protein.Accessions"
## [18] "Number.of.Missed.Cleavages"
## [19] "Theo.MHplus.in.Da"
## [20] "Sequence.Length"
## [21] "Abundance.F1.Sample"
## [22] "Abundance.F2.Sample"
## [23] "Abundance.F3.Sample"
## [24] "Abundance.F4.Sample"
## [25] "Abundance.F5.Sample"
## [26] "Abundance.F6.Sample"
## [27] "Abundances.Count.F1.Sample"
## [28] "Abundances.Count.F2.Sample"
## [29] "Abundances.Count.F3.Sample"
## [30] "Abundances.Count.F4.Sample"
## [31] "Abundances.Count.F5.Sample"
## [32] "Abundances.Count.F6.Sample"
## [33] "Quan.Info"
## [34] "Found.in.File.in.F1"
## [35] "Found.in.File.in.F2"
## [36] "Found.in.File.in.F3"
## [37] "Found.in.File.in.F4"
## [38] "Found.in.File.in.F5"
## [39] "Found.in.File.in.F6"
## [40] "Found.in.Sample.in.S1.F1.Sample"
## [41] "Found.in.Sample.in.S2.F2.Sample"
## [42] "Found.in.Sample.in.S3.F3.Sample"
## [43] "Found.in.Sample.in.S4.F4.Sample"
## [44] "Found.in.Sample.in.S5.F5.Sample"
## [45] "Found.in.Sample.in.S6.F6.Sample"
## [46] "Found.in.Sample.Group.in.S1.F1.Sample"
## [47] "Found.in.Sample.Group.in.S2.F2.Sample"
## [48] "Found.in.Sample.Group.in.S3.F3.Sample"
## [49] "Found.in.Sample.Group.in.S4.F4.Sample"
## [50] "Found.in.Sample.Group.in.S5.F5.Sample"
## [51] "Found.in.Sample.Group.in.S6.F6.Sample"
## [52] "Confidence.by.Search.Engine.Sequest.HT"
## [53] "Charge.by.Search.Engine.Sequest.HT"
## [54] "Delta.Score.by.Search.Engine.Sequest.HT"
## [55] "Delta.Cn.by.Search.Engine.Sequest.HT"
## [56] "Rank.by.Search.Engine.Sequest.HT"
## [57] "Search.Engine.Rank.by.Search.Engine.Sequest.HT"
## [58] "Concatenated.Rank.by.Search.Engine.Sequest.HT"
## [59] "mz.in.Da.by.Search.Engine.Sequest.HT"
## [60] "Delta.M.in.ppm.by.Search.Engine.Sequest.HT"
## [61] "Delta.mz.in.Da.by.Search.Engine.Sequest.HT"
## [62] "RT.in.min.by.Search.Engine.Sequest.HT"
## [63] "Percolator.q.Value.by.Search.Engine.Sequest.HT"
## [64] "Percolator.PEP.by.Search.Engine.Sequest.HT"
## [65] "Percolator.SVMScore.by.Search.Engine.Sequest.HT"
## [66] "XCorr.by.Search.Engine.Sequest.HT"
## [67] "Top.Apex.RT.in.min"


In the code chunk below, we again use the readQFeatures function to import our data into R and create a QFeatures object. We find the abundance data is located in columns 21 to 26 and thus pass this to ecol. After import we annotate the colData.

## Create QFeatures object
sn_qf <- readQFeatures(table = sn_peptide,
                       ecol = 21:26,
                       sep = "\t",
                       name = "peptides_raw")

## Clean sample names
colnames(sn_qf[["peptides_raw"]]) <- paste0("S", 1:6)

## Annotate samples
sn_qf$sample <- paste0("S", 1:6)

sn_qf$condition <- rep(c("Treated", "Control"), each = 3)

## Verify and allocate colData to initial SummarizedExperiment
colData(sn_qf)

## DataFrame with 6 rows and 2 columns
##         sample   condition
##    <character> <character>
## S1          S1     Treated
## S2          S2     Treated
## S3          S3     Treated
## S4          S4     Control
## S5          S5     Control
## S6          S6     Control

colData(sn_qf[["peptides_raw"]]) <- colData(sn_qf)


### Preliminary data exploration

Next, we check the names of the features within the peptide-level rowData. These features differ from those found at the PSM-level and users should be aware that they have reduced post-search control over the quality of PSMs included in the peptide quantitation, and which method of aggregation is used to define these. Proteome Discoverer uses the sum of PSM quantitative values to calculate peptide-level values. Other third-party softwares may use different methods.

## Find out what information was imported
sn_qf[["peptides_raw"]] %>%
  rowData() %>%
  colnames()

##  [1] "Peptide.Groups.Peptide.Group.ID"
##  [2] "Checked"
##  [3] "Tags"
##  [4] "Confidence"
##  [5] "PSM.Ambiguity"
##  [6] "Sequence"
##  [7] "Modifications"
##  [8] "Modifications.all.possible.sites"
##  [9] "Qvality.PEP"
## [10] "Qvality.q.value"
## [11] "SVM_Score"
## [12] "Number.of.Protein.Groups"
## [13] "Number.of.Proteins"
## [14] "Number.of.PSMs"
## [15] "Master.Protein.Accessions"
## [16] "Master.Protein.Descriptions"
## [17] "Protein.Accessions"
## [18] "Number.of.Missed.Cleavages"
## [19] "Theo.MHplus.in.Da"
## [20] "Sequence.Length"
## [21] "Abundances.Count.F1.Sample"
## [22] "Abundances.Count.F2.Sample"
## [23] "Abundances.Count.F3.Sample"
## [24] "Abundances.Count.F4.Sample"
## [25] "Abundances.Count.F5.Sample"
## [26] "Abundances.Count.F6.Sample"
## [27] "Quan.Info"
## [28] "Found.in.File.in.F1"
## [29] "Found.in.File.in.F2"
## [30] "Found.in.File.in.F3"
## [31] "Found.in.File.in.F4"
## [32] "Found.in.File.in.F5"
## [33] "Found.in.File.in.F6"
## [34] "Found.in.Sample.in.S1.F1.Sample"
## [35] "Found.in.Sample.in.S2.F2.Sample"
## [36] "Found.in.Sample.in.S3.F3.Sample"
## [37] "Found.in.Sample.in.S4.F4.Sample"
## [38] "Found.in.Sample.in.S5.F5.Sample"
## [39] "Found.in.Sample.in.S6.F6.Sample"
## [40] "Found.in.Sample.Group.in.S1.F1.Sample"
## [41] "Found.in.Sample.Group.in.S2.F2.Sample"
## [42] "Found.in.Sample.Group.in.S3.F3.Sample"
## [43] "Found.in.Sample.Group.in.S4.F4.Sample"
## [44] "Found.in.Sample.Group.in.S5.F5.Sample"
## [45] "Found.in.Sample.Group.in.S6.F6.Sample"
## [46] "Confidence.by.Search.Engine.Sequest.HT"
## [47] "Charge.by.Search.Engine.Sequest.HT"
## [48] "Delta.Score.by.Search.Engine.Sequest.HT"
## [49] "Delta.Cn.by.Search.Engine.Sequest.HT"
## [50] "Rank.by.Search.Engine.Sequest.HT"
## [51] "Search.Engine.Rank.by.Search.Engine.Sequest.HT"
## [52] "Concatenated.Rank.by.Search.Engine.Sequest.HT"
## [53] "mz.in.Da.by.Search.Engine.Sequest.HT"
## [54] "Delta.M.in.ppm.by.Search.Engine.Sequest.HT"
## [55] "Delta.mz.in.Da.by.Search.Engine.Sequest.HT"
## [56] "RT.in.min.by.Search.Engine.Sequest.HT"
## [57] "Percolator.q.Value.by.Search.Engine.Sequest.HT"
## [58] "Percolator.PEP.by.Search.Engine.Sequest.HT"
## [59] "Percolator.SVMScore.by.Search.Engine.Sequest.HT"
## [60] "XCorr.by.Search.Engine.Sequest.HT"
## [61] "Top.Apex.RT.in.min"


We also determine the number of PSMs, peptides and proteins represented within the initial data. Since identical peptide sequences with different modifications are stored as separate entities, the output of dim will not tell us the number of peptides. Instead, we need to consider only unique peptide sequence entries, as demonstrated in the code chunk below.

## Determine the number of PSMs
original_psms <- sn_qf[["peptides_raw"]] %>%
  rowData() %>%
  as_tibble() %>%
  pull(Number.of.PSMs) %>%
  sum()

## Determine the number of peptides
original_peps <- sn_qf[["peptides_raw"]] %>%
  rowData() %>%
  as_tibble() %>%
  pull(Sequence) %>%
  unique() %>%
  length() %>%
  as.numeric()

## Determine the number of proteins
original_prots <- sn_qf[["peptides_raw"]] %>%
  rowData() %>%
  as_tibble() %>%
  pull(Master.Protein.Accessions) %>%
  unique() %>%
  length() %>%
  as.numeric()

## View
original_psms

## [1] 144302

original_peps

## [1] 20312

original_prots

## [1] 3941


Thus, the search identified 144302 PSMs corresponding to 20312 peptides and 3941 proteins. Finally, we take a look at some of the key parameters applied during the identification search. This is an important verification step, particularly for those using publicly available data with limited access to parameter settings.

## Check missed cleavages
sn_qf[["peptides_raw"]] %>%
  rowData() %>%
  as_tibble() %>%
  pull(Number.of.Missed.Cleavages) %>%
  table()

## .
##     0    1  2
## 22055 1248 72

## Check precursor mass tolerance
sn_qf[["peptides_raw"]] %>%
  rowData() %>%
  as_tibble() %>%
  pull(Delta.M.in.ppm.by.Search.Engine.Sequest.HT) %>%
  summary()

##     Min. 1st Qu. Median   Mean 3rd Qu.   Max.
## -9.9600  -0.2500 0.1500 0.6576  0.6900 9.9900

## Check fragment mass tolerance
sn_qf[["peptides_raw"]] %>%
  rowData() %>%
  as_tibble() %>%
  pull(Delta.mz.in.Da.by.Search.Engine.Sequest.HT) %>%
  summary()

##       Min.     1st Qu.   Median      Mean   3rd Qu.      Max.
## -0.0113400 -0.0001400 0.0000900 0.0006618 0.0004800 0.0142300

## Check peptide confidence allocations
sn_qf[["peptides_raw"]] %>%
  rowData() %>%
  as_tibble() %>%
  pull(Confidence) %>%
  table()

## .
##  High
## 23375


The preliminary data is as expected so we continue on to evaluate the quality of the raw data.

### Experimental quality control checks

### Quality control of the raw mass spectrometry data

To briefly assess the quality of the raw mass spectrometry data from which the search results were derived, we create simple plots. In contrast to the previous PSM processing workflow, we do not have access to information about ion injection times from the peptide-level file. However, we can still look at the peptide delta mass across retention time, as well as the frequency of peptides across the retention time gradient.

## Plot scatter plot of mass accuracy
sn_qf[["peptides_raw"]] %>%
  rowData() %>%
  as_tibble() %>%
  ggplot(aes(x = RT.in.min.by.Search.Engine.Sequest.HT,
             y = Delta.M.in.ppm.by.Search.Engine.Sequest.HT)) +
  geom_point(size = 0.5, shape = 4) +
  geom_hline(yintercept = 5, linetype = "dashed", color = "red") +
  geom_hline(yintercept = -5, linetype = "dashed", color = "red") +
  labs(x = "RT (min)", y = "Delta precursor mass (ppm)") +
  scale_x_continuous(limits = c(0, 120), breaks = seq(0, 120, 20)) +
  scale_y_continuous(limits = c(-10, 10), breaks = c(-10, -5, 0, 5, 10)) +
  ggtitle("Peptide retention time against delta precursor mass") +
  theme_bw()

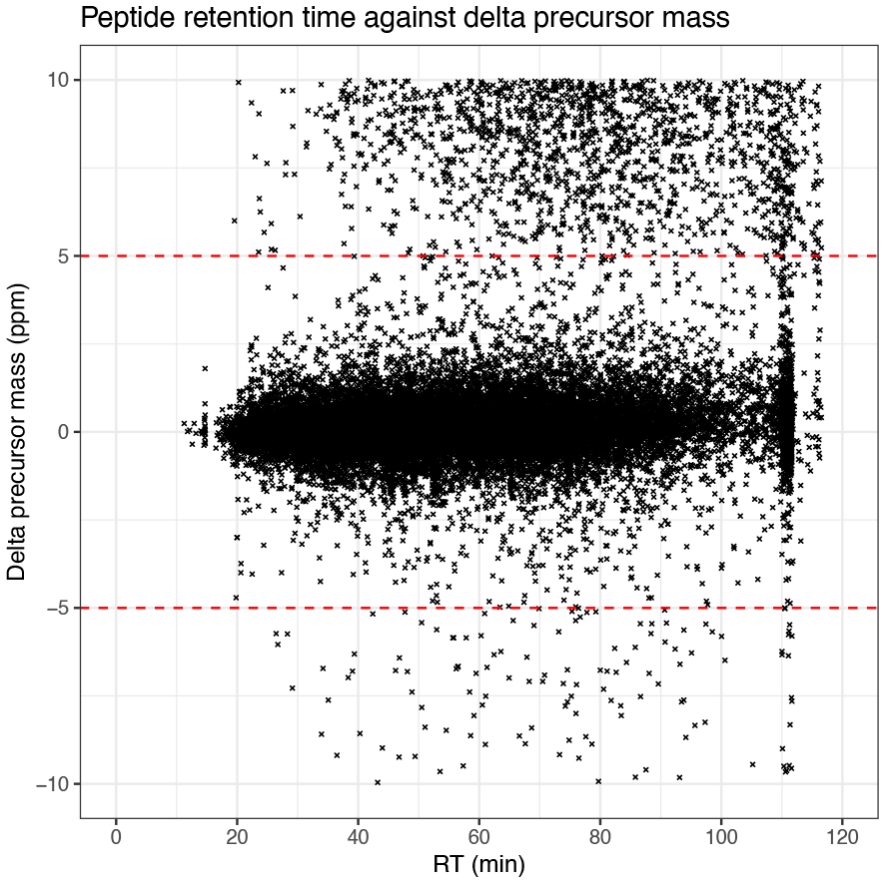


## Plot histogram of peptide retention time
sn_qf[["peptides_raw"]] %>%
  rowData() %>%
  as_tibble() %>%
  ggplot(aes(x = RT.in.min.by.Search.Engine.Sequest.HT)) +
  geom_histogram(binwidth = 1) +
  labs(x = "RT (min)", y = "Frequency") +
  scale_x_continuous(breaks = seq(0, 120, 20)) +
  ggtitle("Peptide frequency across retention time") +
  theme_bw()

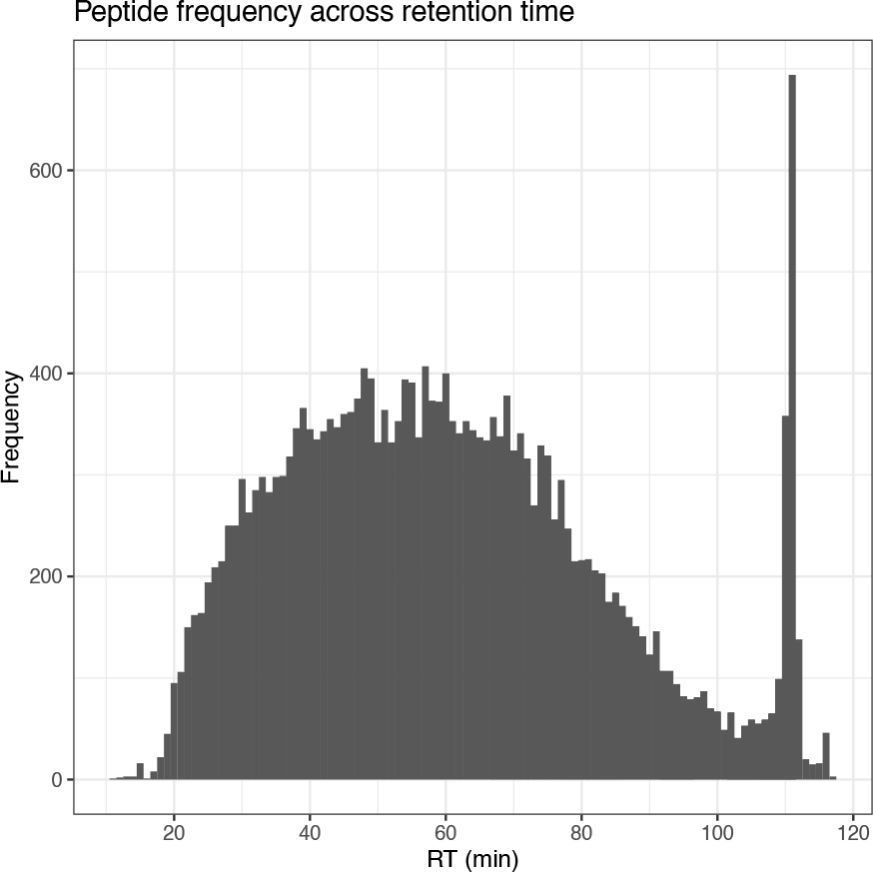


For a more in-depth discussion of these plots users should refer back to the TMT processing workflow. Since neither plot indicates any major problems with the MS runs, we continue on to basic data cleaning.

### Basic data cleaning

As discussed in detail above, there are several basic data cleaning steps which are non-specific and should be applied to all quantitative datasets, regardless of the quantitation method or data level (PSM, peptide or protein). These steps are as follows:

1. Removal of features without a master protein accession
2. Removal of features corresponding to protein groups which contain a contaminant
3. Removal of features without quantitative data
4. (Optional) Removal of features which are not unique to a protein group
5. Removal of features not allocated rank 1 during the identification search
6. Removal of features not annotated as unambiguous

In addition to these standard steps, LFQ data should be filtered to remove peptides that were not quantified based on a monoisotopic peak. The monoisotopic peak is that which comprises the most abundant natural isotope of each constituent element. For bottom-up proteomics, this typically translates to the peptides containing carbon-12 and nitrogen-14. When the different isotopes are well resolved, the monoisotopic peak usually provides the most accurate measurement.

Before we remove any data, we first create a second copy of the original SummarizedExperiment, as to retain a copy of the raw data for reference. As before we use the addAssay function.

## Add second copy of data to be filtered
data_copy <- sn_qf[["peptides_raw"]]

sn_qf <- addAssay(x = sn_qf,
                  y = data_copy,
                  name = "peptides_filtered")

## Verify
sn_qf

## An instance of class QFeatures containing 2 assays:
##  [1] peptides_raw: SummarizedExperiment with 23375 rows and 6 columns
##  [2] peptides_filtered: SummarizedExperiment with 23375 rows and 6 columns


Here, cleaning is done is two steps. The first is the removal of contaminant proteins using the self-defined find_cont function. Refer back to the TMT processing workflow for more details.

## Store row indices of peptides matched to a contaminant-containing protein group
cont_peptides <- find_cont(sn_qf[["peptides_filtered"]], cont_acc)

## Remove these rows from the data
if (length(cont_peptides) > 0)
  sn_qf[["peptides_filtered"]] <- sn_qf[["peptides_filtered"]][-cont_peptides, ]


Second, we carry out all remaining cleaning using the filterFeatures function as before.

sn_qf <- sn_qf %>%
  filterFeatures(~ !Master.Protein.Accessions == "",
                 i = "peptides_filtered") %>%
  filterFeatures(~ !Quan.Info == "NoQuanValues",
                 i ="peptides_filtered") %>%
  filterFeatures(~ !Quan.Info == "NoneMonoisotopic",
                 i = "peptides_filtered") %>%
  filterFeatures(~ Number.of.Protein.Groups == 1,
                 i = "peptides_filtered") %>%
  filterFeatures(~ Rank.by.Search.Engine.Sequest.HT == 1,
                 i = "peptides_filtered") %>%
  filterFeatures(~ PSM.Ambiguity == "Unambiguous",
                 i = "peptides_filtered")


As before, we check to see whether additional annotations remain within the “Quan.Info” column.

## Check for remaining annotations
sn_qf[["peptides_filtered"]] %>%
  rowData %>%
  as_tibble() %>%
  pull(Quan.Info) %>%
  table()

## .
##
## 17999


### Assessing the impact of non-specific data cleaning

As in the previous example, we assess the impact that cleaning has had on the data. Specifically, we determine the number and proportion of PSMs, peptides and proteins lost. Again, when we refer to the number of peptides we only consider unique peptide sequences, not those that differ in their modifications.

## Determine number of PSMs, peptides and proteins remaining
psms_remaining <- sn_qf[["peptides_filtered"]] %>%
  rowData() %>%
  as_tibble() %>%
  pull(Number.of.PSMs) %>%
  sum()

peps_remaining <- sn_qf[["peptides_filtered"]] %>%
  rowData() %>%
  as_tibble() %>%
  pull(Sequence) %>%
  unique() %>%
  length() %>%
  as.numeric()

prots_remaining <- sn_qf[["peptides_filtered"]] %>%
  rowData() %>%
  as_tibble() %>%
  pull(Master.Protein.Accessions) %>%
  unique() %>%
  length() %>%
  as.numeric()

## Determine the number of proportion of PSMs, peptides and proteins removed
psms_removed <- original_psms - psms_remaining
psms_removed_prop <- ((psms_removed /original_psms) * 100) %>%
  round(digits = 2)

peps_removed <- original_peps - peps_remaining
peps_removed_prop <- ((peps_removed / original_peps) * 100) %>%
  round(digits = 2)

prots_removed <- original_prots - prots_remaining
prots_removed_prop <- ((prots_removed / original_prots) * 100) %>%
  round(digits = 2)

## Present in a table
data.frame("Feature" = c("PSMs",
                         "Peptides",
                         "Proteins"),
           "Number lost" = c(psms_removed,
                             peps_removed,
                             prots_removed),
           "Percentage lost" = c(psms_removed_prop,
                                 peps_removed_prop,
                                 prots_removed_prop),
           "Number remaining" = c(psms_remaining,
                                  peps_remaining,
                                  prots_remaining))

##    Feature Number.lost Percentage.lost Number.remaining
## 1     PSMs       28140           19.50           116162
## 2 Peptides        3767           18.55            16545
## 3 Proteins         690           17.51             3251


### Peptide quality control filtering

When extracting data from the peptide-level .txt file rather than aggregating up from a PSM file, additional parameters exist within the peptide rowData. Such parameters include Quality PEP, Quality q-value, and SVM score, as well as similar scoring parameters provided by the search engine. Although we will not complete additional filtering based on these parameters in this workflow, users may wish to explore this option.

### Managing missing data

Having cleaned the peptide-level data we now move onto the management of missing data. This is of particular importance for LFQ workflows where the missing value challenge is amplified by intrinsic variability between independent MS runs. As before, the management of missing data can be divided into three steps: 1) exploring the presence and distribution of missing values, (2) filtering out missing values, and (3) optional imputation.

### Exploring the presence of missing values

The aim of the first step is to determine how many missing values are present within the data, and how they are distributed between samples and/or conditions.

## Are there any NA values within the peptide data?
sn_qf[["peptides_filtered"]] %>%
  assay() %>%
  anyNA()

## [1] TRUE

## How many NA values are there within the peptide data?
sn_qf[["peptides_filtered"]] %>%
  nNA()

## $nNA
## DataFrame with 1 row and 2 columns
##         nNA       pNA
##   <integer> <numeric>
## 1     15863   14.6888
##
## $nNArows
## DataFrame with 17999 rows and 3 columns
##              name       nNA       pNA
##       <character> <integer> <numeric>
## 1               1         4   66.6667
## 2               2         1   16.6667
## 3               3         0    0.0000
## 4               4         1   16.6667
## 5               5         0    0.0000
## ...           ...       ...       ...
## 17995       23371         0         0
## 17996       23372         0         0
## 17997       23373         0         0
## 17998       23374         0         0
## 17999       23375         0         0
##
## $nNAcols
## DataFrame with 6 rows and 3 columns
##          name       nNA       pNA
##   <character> <integer> <numeric>
## 1          S1      3699   20.5511
## 2          S2      1945   10.8062
## 3          S3      2048   11.3784
## 4          S4      3674   20.4122
## 5          S5      2673   14.8508
## 6          S6      1824   10.1339


As expected, the LFQ data contains a higher proportion of missing values as compared to the TMT-labelled data. There are 15863 missing (NA) values within the data, which corresponds to 15%. We check for sample- and condition-specific biases in the distribution of these NA values.

## Plot histogram to visualize sample-specific distribution of NAs
nNA(sn_qf[["peptides_filtered"]])$nNAcols %>%
  as_tibble() %>%
  mutate(Condition = rep(c("Treated", "Control"), each = 3)) %>%
  ggplot(aes(x = name, y = pNA, group = Condition, fill = Condition)) +
  geom_bar(stat = "identity") +
  geom_hline(yintercept = 14.7, linetype = "dashed", color = "red") +
  labs(x = "Sample", y = "Missing values (%)") +
  theme_bw()

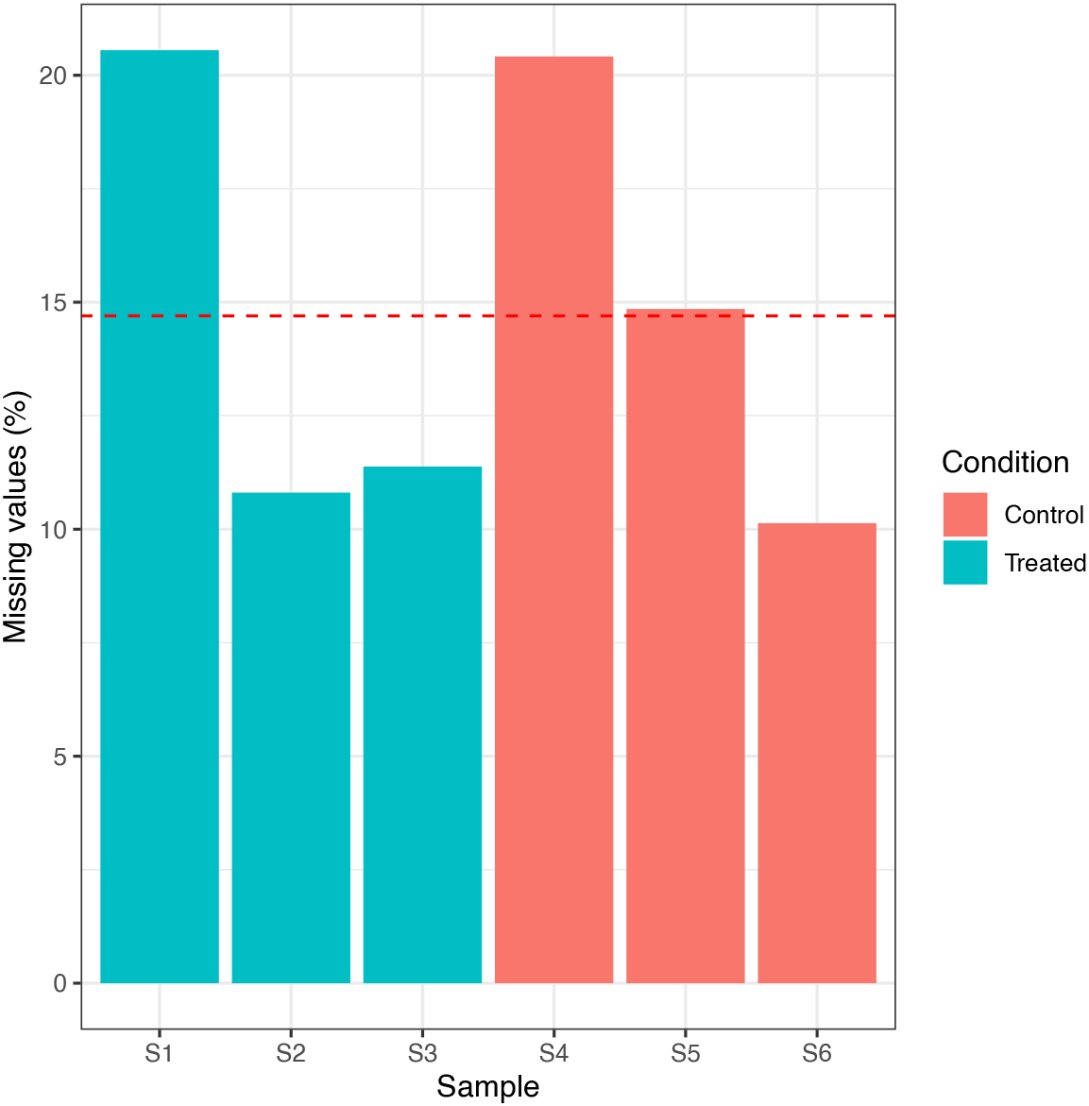


Whilst S1 and S4 have a slightly higher proportion of missing values, all of the samples are within an acceptable range to continue. Again, there is no evidence of a condition-specific bias in the data.

### Filtering out missing values

We next filter out features, here peptides, which comprise 20% or more missing values.

## Check how many peptides we will remove
which(nNA(sn_qf[["peptides_filtered"]])$nNArows$pNA >= 20) %>%
  length()

## [1] 4364

## Remove peptides with 2 or more NA values
sn_qf <- sn_qf %>%
  filterNA(pNA = 0.2,
           i = "peptides_filtered")


### Imputation (optional)

Finally, we check how many missing values remain in the data before making a decision as to whether imputation is required.

nNA(sn_qf[["peptides_filtered"]])$nNA

## DataFrame with 1 row and 2 columns
##         nNA       pNA
##   <integer> <numeric>
## 1      2452   2.99719


There are 2452 missing values remaining. The presence of proteins with single or low peptide support means that some of these NA values will likely be propagated upward during aggregation. Whilst NA values were traditionally problematic during the application of downstream statistical methods, there are now a number of algorithms that allow statistics to be completed on data containing missing values. For example, the MSqRob2 ^24^^,^^25^^,^^32^ package facilitates statistical differential expression analysis on datasets without the need for imputation and functions within the QFeatures infrastructure. Nevertheless, for the purpose of demonstration, we here choose to impute the raw intensity data.

As eluded to above, the most appropriate method to determine such probable values is dependent upon why the value is missing, that is whether it is MCAR or MNAR. Although the optimal imputation method is specific to each dataset, left-censored methods (e.g. minimal value approaches, limit of detection) have proven favorable for data with a high proportion of MNAR values whilst hot deck methods (e.g. k-nearest neighbours, random forest, maximum likelihood methods) are more appropriate when the majority of missing data is MCAR [e.g. Refs. 23, 33]. Within the QFeatures infrastructure imputation is carried out by passing the data to the impute function, please see ?impute for more information. To see which imputation methods are supported by this function we use the following code:

## Find out available imputation methods
MsCoreUtils::imputeMethods()

## [1] "bpca" "knn"  "QRILC" "MLE"   "MLE2" "MinDet" "MinProb"
## [8] "min"  "zero" "mixed" "nbavg" "with" "RF"     "none"


Unfortunately, it is very challenging to determine the reason(s) behind missing data, and in most cases experiments contain a mixture of MCAR and MNAR. For LFQ data where little is know about the cause of missing values it is advisable to use methods optimised for MCAR. Here we will use the baseline k-nearest neighbours (k-NN) imputation on the raw peptide intensities. Of note, users who wish to utilise an alternative imputation method should check whether their selected method has a requirement for normality. If the method requires data to display a normal distribution, users must log2 transform the data prior to imputation.

## Impute missing values using kNN
sn_qf <- impute(sn_qf,
                method = "knn",
                i = "peptides_filtered",
                name = "peptides_imputed")


Following imputation we check to ensure that the distribution of the data has not dramatically changed. To do so we create a density plot of the data pre- and post-imputation.

## visualise the impact of imputation
par(mfrow = c(1, 2))

sn_qf[["peptides_filtered"]] %>%
  assay() %>%
  log2() %>%
  plotDensities(main = "Pre-imputation",
                legend = FALSE)

sn_qf[["peptides_imputed"]] %>%
  assay() %>%
  log2() %>%
  plotDensities(main = "Post-imputation",
                legend = "topright")

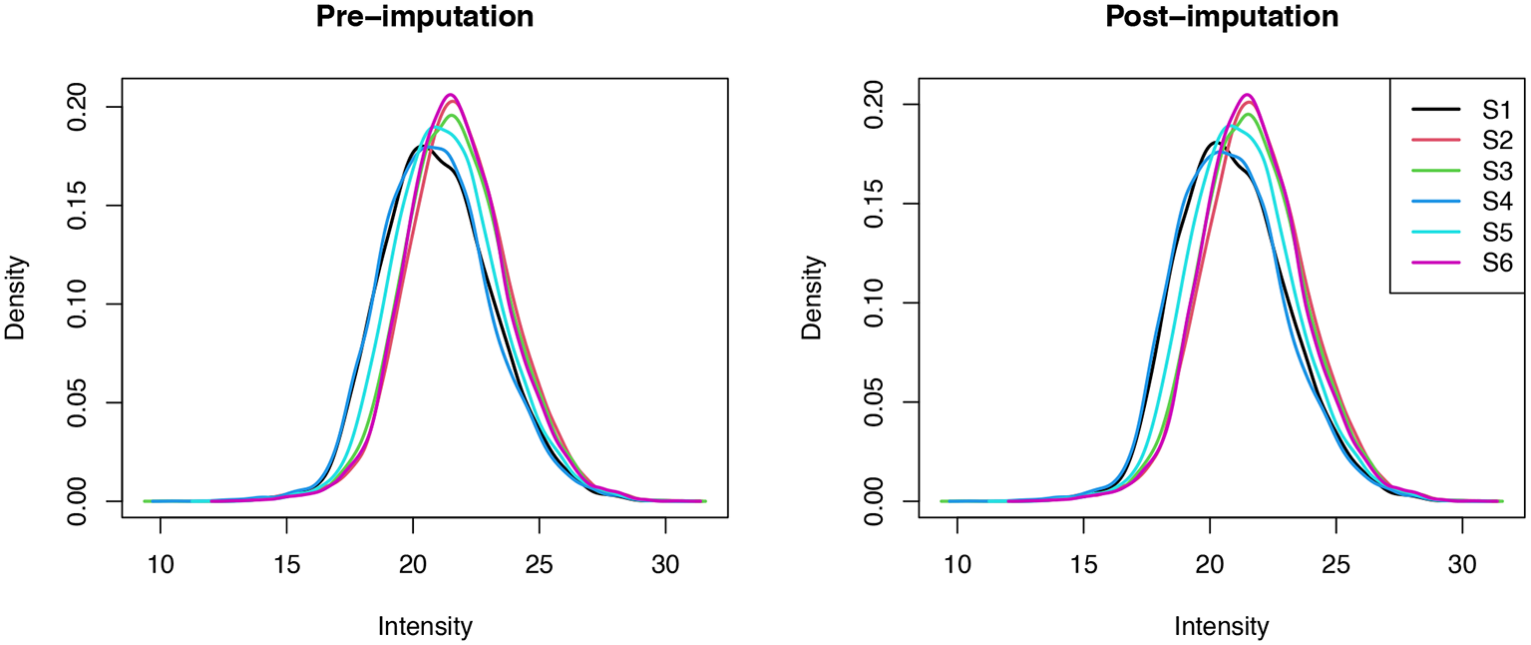


From this plot the change in the data appears to be minimal. We can further validate this by comparing the summary statistics of the data pre- and post-imputation.

## Determine the impact of imputation on summary statistics
pre_imputation_summary <- sn_qf[["peptides_filtered"]] %>%
  assay() %>%
  longFormat() %>%
  group_by(colname) %>%
  summarise(sum_intensity = sum(value, na.rm = TRUE),
            max_intensity = max(value, na.rm = TRUE),
            median_intensity = median(value, na.rm = TRUE))

post_imputation_summary <- sn_qf[["peptides_imputed"]] %>%
  assay() %>%
  longFormat() %>%
  group_by(colname) %>%
  summarise(sum_intensity = sum(value, na.rm = TRUE),
            max_intensity = max(value, na.rm = TRUE),
            median_intensity = median(value, na.rm = TRUE))

print(pre_imputation_summary)

## # A tibble: 6 x 4
##   colname sum_intensity max_intensity median_intensity
##   <chr>           <dbl>         <dbl>            <dbl>
## 1 S1       98919496611.    1477162278.        1948794.
## 2 S2      155722262777.    1678988256.        3553168.
## 3 S3      145509803642.    1804842981.        3251578.
## 4 S4       94892286529.    1087946291.        1873948.
## 5 S5      121590387110.    1307181986         2503109
## 6 S6      143538084562.    1608003894.        3282077.

print(post_imputation_summary)

## # A tibble: 6 x 4
##   colname sum_intensity max_intensity median_intensity
##   <chr>           <dbl>         <dbl>            <dbl>
## 1 S1       99811359317.    1477162278.        1812200.
## 2 S2      156124619343.    1678988256.        3478920.
## 3 S3      145754574641.    1804842981.        3201591
## 4 S4       96201734159.    1087946291.        1720642.
## 5 S5      122113590867.    1307181986         2440628.
## 6 S6      143994721088.    1608003894.        3199278.


Comparison of the two tables reveals minimal change within the data. However, we find that S1 and S4 display greater differences between pre- and post-imputation statistics because of the higher number of missing values which required imputation.

### Logarithmic transformation of quantitative data

In the following code chunk we log2 transform the peptide-level data to generate a near-normal distribution within the quantitative data. This is necessary prior to the use of robustSummary aggregation.

## log2 transform the quantitative data
sn_qf <- logTransform(object = sn_qf,
                      base = 2,
                      i = "peptides_imputed",
                      name = "log_peptides")

## Verify
sn_qf

## An instance of class QFeatures containing 4 assays:
##  [1] peptides_raw: SummarizedExperiment with 23375 rows and 6 columns
##  [2] peptides_filtered: SummarizedExperiment with 13635 rows and 6 columns
##  [3] peptides_imputed: SummarizedExperiment with 13635 rows and 6 columns
##  [4] log_peptides: SummarizedExperiment with 13635 rows and 6 columns


### Aggregation of peptide to protein

Now that we are happy with the peptide-level data, we aggregate upward to proteins using the aggregateFeatures function.

## Aggregate peptide to protein
sn_qf <- aggregateFeatures(sn_qf,
                           i = "log_peptides",
                           fcol = "Master.Protein.Accessions",
                           name = "log_proteins",
                           fun = MsCoreUtils::robustSummary,
                           na.rm = TRUE)

## Your row data contain missing values. Please read the relevant
## section(s) in the aggregateFeatures manual page regarding the effects
## of missing values on data aggregation.

## Verify
sn_qf

## An instance of class QFeatures containing 5 assays:
##  [1] peptides_raw: SummarizedExperiment with 23375 rows and 6 columns
##  [2] peptides_filtered: SummarizedExperiment with 13635 rows and 6 columns
##  [3] peptides_imputed: SummarizedExperiment with 13635 rows and 6 columns
##  [4] log_peptides: SummarizedExperiment with 13635 rows and 6 columns
##  [5] log_proteins: SummarizedExperiment with 2837 rows and 6 columns


### Normalisation of quantitative data

Finally, we complete the data processing by normalising quantitation between samples. This is done using the " center.median" method via the normalize function.

## normalize protein-level quantitation data
sn_qf <- normalize(sn_qf,
                   i = "log_proteins",
                   name = "log_norm_proteins",
                   method = "center.median")

## Verify
sn_qf

## An instance of class QFeatures containing 6 assays:
##  [1] peptides_raw: SummarizedExperiment with 23375 rows and 6 columns
##  [2] peptides_filtered: SummarizedExperiment with 13635 rows and 6 columns
##  [3] peptides_imputed: SummarizedExperiment with 13635 rows and 6 columns
##  [4] log_peptides: SummarizedExperiment with 13635 rows and 6 columns
##  [5] log_proteins: SummarizedExperiment with 2837 rows and 6 columns
##  [6] log_norm_proteins: SummarizedExperiment with 2837 rows and 6 columns


The final dataset is comprised of 2837 proteins. We will save the protein-level SummarizedExperiment file as well as exporting the final QFeatures object.

## Save protein-level SE
sn_proteins <- sn_qf[["log_norm_proteins"]]

## Export TMT final QFeatures object
save(sn_qf, file = "sn_qf.rda")


## Exploration of protein data

Having described the processing steps for quantitative proteomics data, we next demonstrate how to explore the protein-level data prior to statistical analysis. For this we will utilise the TMT-labelled cell pellet dataset since it contains a greater number of proteins.

### Correlation plots

We will first generate correlation plots between pairs of samples. To do this we use the corrplot package to calculate and plot the Pearson’s correlation coefficient between each sample pair. The cor function within the corrplot package will create a correlation matrix but requires a data.frame, matrix or a vector of class numeric as input. To convert the QFeatures assay data into a data.frame we use the as. data.frame function.

## Convert TMT CP protein assay into a dataframe
prot_df <- cp_qf[["log_norm_proteins"]] %>%
  assay() %>%
  as.data.frame()

## Calculate a correlation matrix between samples
corr_matrix <- cor(prot_df,
                   method = "pearson",
                   use = "pairwise.complete.obs")

print(corr_matrix)

##           S1        S2        S3        S4        S5        S6
## S1 1.0000000 0.9863382 0.9927432 0.9447089 0.9627190 0.9628944
## S2 0.9863382 1.0000000 0.9886960 0.9206049 0.9535162 0.9501522
## S3 0.9927432 0.9886960 1.0000000 0.9344640 0.9551061 0.9548680
## S4 0.9447089 0.9206049 0.9344640 1.0000000 0.9822722 0.9867422
## S5 0.9627190 0.9535162 0.9551061 0.9822722 1.0000000 0.9928376
## S6 0.9628944 0.9501522 0.9548680 0.9867422 0.9928376 1.0000000


Now we can visualise the correlation data using pairwise scatter plots and a correlation heat map.

## Plot correlation between two samples - S1 and S2 used as example
prot_df %>%
  ggplot(aes(x = `S1`, y = `S2`)) +
  geom_point(colour = "grey45", size = 0.5) +
  geom_abline(intercept = 0, slope = 1) +
  theme(panel.grid.major = element_blank(),
        panel.grid.minor = element_blank(),
        plot.background = element_rect(fill = "white"),
        panel.background = element_rect(fill = "white"),
        axis.title.x = element_text(size = 15, vjust = -2),
        axis.title.y = element_text(size = 15, vjust = 3),
        axis.text.x = element_text(size = 12, vjust = -1),
        axis.text.y = element_text(size = 12),
        axis.line = element_line(linewidth = 0.5, colour = "black"),
        plot.margin = margin(10, 10, 10, 10)) +
  xlim(-7.5, 5) +
  ylim(-7.5, 5) +
  labs(x = "log2(abundance S1)", y = "log2(abundance S2)") +
  coord_fixed(ratio = 1)

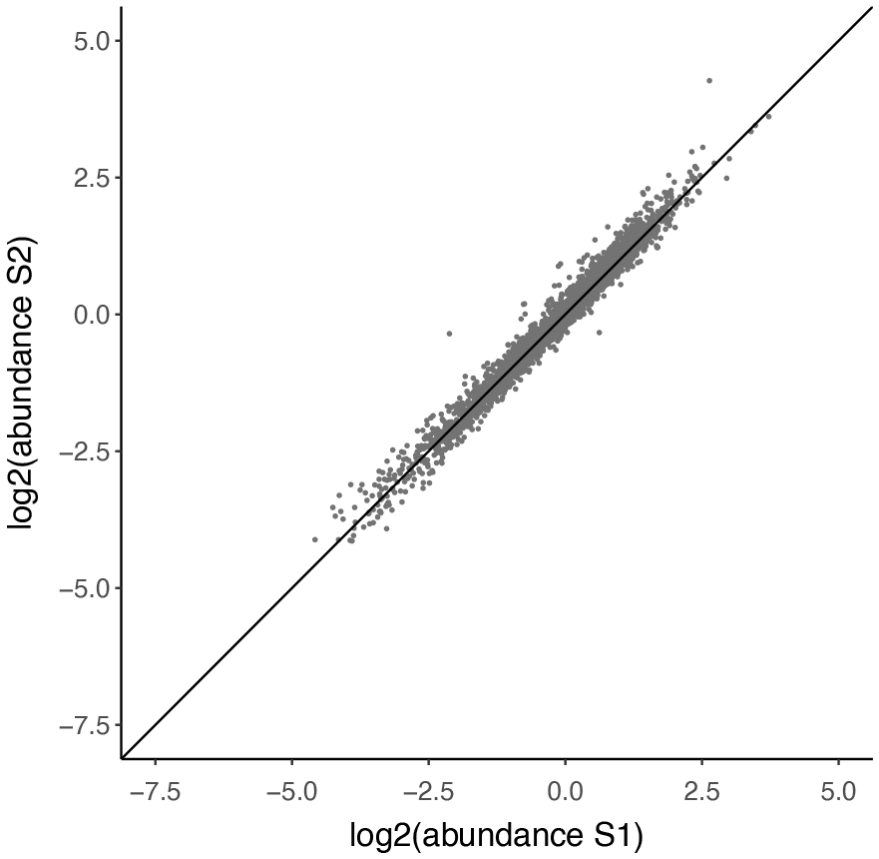


## Create colour palette for continuum
col <- colorRampPalette(c("#BB4444", "#EE9988", "#FFFFFF",
                          "#77AADD", "#4477AA"))

## Plot all pairwise correlations
prot_df %>%
  cor(method = "pearson",
      use = "pairwise.complete.obs") %>%
  corrplot(method = "color",
           col = col(200),
           type = "upper",
           addCoef.col = "white",
           diag = FALSE,
           tl.col = "black",
           tl.srt = 45,
           outline = TRUE)

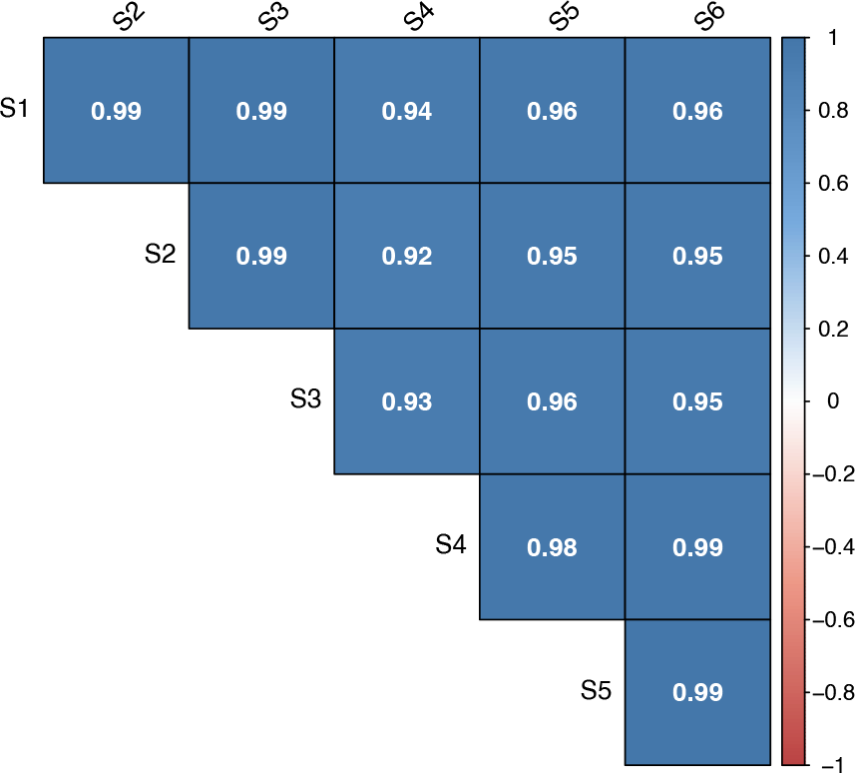


From these plots we can see that all replicate pairs have a Pearson’s correlation coefficient >0.98 whilst the correlation between pairs of control and treated samples is somewhat lower. Users may interpret this information as an early indication that some proteins may be differentially abundant between the two groups.

Of note, whilst correlation is widely applied as a measure of reproducibility, users are reminded that correlation coefficients alone are not informative of reproducibility. ^34^^,^^35^ This is especially true for expression proteomics data in which high correlation values are likely due to the majority of proteins remaining at similar levels regardless of cellular perturbation. Users are directed to Ref. 36 for additional information regarding how to determine the calculation of experimental reproducibility.

### Principal Component Analysis

Principal Component Analysis (PCA) is a dimensionality reduction method which aims to simplify complex datasets and facilitate the visualisation of multi-dimensional data. Here we use the prcomp function from the stats package to perform the PCA. Since PCA does not accept missing values and we did not impute the TMT data, the filterNA function can be used to remove any missing values that may be present in the protein-level data. We then extract and transpose the assay data before passing it to the prcomp function to carry out PCA.

## Carry out principal component analysis
prot_pca <- cp_qf[["log_norm_proteins"]] %>%
  filterNA() %>%
  assay() %>%
  t() %>%
  prcomp(scale = TRUE, center = TRUE)


We can get an idea of the outcome of the PCA by running the summary function on the results of the PCA.

## Get a summary of the PCA
summary(prot_pca)

## Importance of components:
##                           PC1     PC2     PC3      PC4      PC5       PC6
## Standard deviation    42.4845 26.7522 18.1650 15.15893 14.44395 5.474e-14
## Proportion of Variance 0.5488  0.2176  0.1003  0.06987  0.06343 0.000e+00
## Cumulative Proportion  0.5488  0.7664  0.8667  0.93657  1.00000 1.000e+00


Finally, we create a PCA plot. For additional PCA exploration and visualization tools users are directed to the factoextra package.

## Generate dataframe of each sample's PCA results
pca_df <- as.data.frame(prot_pca$x)

## Annotate samples with their corresponding condition
pca_df$condition <- cp_qf[["psms_raw"]]$condition

## Generate a PCA plot using PC1 and PC2
pca_df %>%
  ggplot(aes(x = PC1, y = PC2, colour = condition)) +
  geom_point(size = 4) +
  scale_color_brewer(palette = "Set2") +
  labs(colour = "Condition") +
  geom_hline(yintercept = 0, linetype = "dashed") +
  geom_vline(xintercept = 0, linetype = "dashed") +
  guides(colour = guide_legend(override.aes = list(size = 3))) +
  labs(x = "PC1 (42.5 %)", y = "PC2 (26.8 %)") +
  ggtitle("Protein-level PCA plot") +
  xlim(-100, 100) +
  ylim(-100, 100) +
  coord_fixed(ratio = 1) +
  theme_bw()

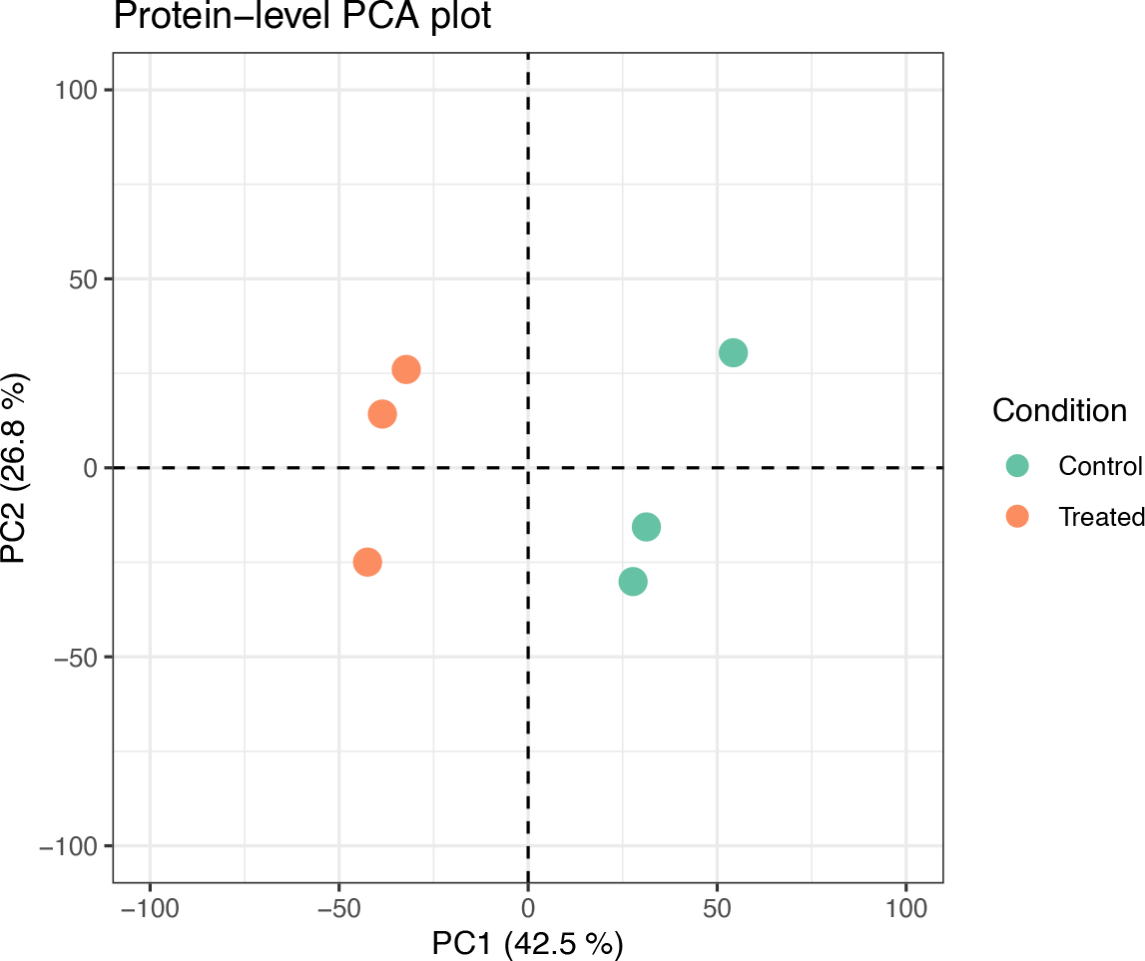


### Exploring potential batch effects

Before carrying out differential expression analysis, it is first necessary to explore the presence of batch effects within the data. Batch effects are derived from non-biological factors which impact the experimental data. These include reagents, instrumentation, personnel and laboratory conditions. In most cases the increased variation caused by batch effects will lead to reduced downstream statistical power. On the other hand, if correlated with the experimental sub- groups, batch effects can also lead to confounded results and the incorrect biological interpretation of differential expression. ^37^

Given that the use-case data was derived from a small experiment with only six samples and a single TMTplex, there are minimal batch effects to explore here. For users analysing larger experiments completed over long period of time, across several laboratories/individuals, or using multiple TMTplex reagents, it is advisable to annotate the PCA plot with all potential batch factors. If data is found to cluster based on any of these factors, batch effects should be incorporated into downstream analyses. For example, users can apply the removeBatchEffect function from the limma package.

### Discovery and biological interpretation of differentially abundant proteins

The last section of this workflow demonstrates how to gain biological insights from the resulting list of proteins. Again we will utilise the TMT-labelled cell pellet data, although the process would be exactly the same for the LFQ supernatant protein list. Users are reminded that although referred to as differential ‘expression’ analysis, abundance is determined by both protein synthesis and degradation.

### Extracting and organising protein-level data

We first extract the protein-level SummarizedExperiment from the cell pellet TMT QFeatures object and specify the study factors. Here we are interested in discovering differences between conditions, control and treated. As well as assigning these conditions to each sample, we can define the control group as the reference level such that differential abundance is reported relative to the control. This means that when we get the results of the statistical analysis, ‘upregulated’ will refer to increased abundance in treated cells relative to control controls.

## Extract protein-level data and associated colData
cp_proteins <- cp_qf[["log_norm_proteins"]]
colData(cp_proteins) <- colData(cp_qf[["log_norm_proteins"]])

## Create factor of interest
cp_proteins$condition <- factor(cp_proteins$condition)

## Check which level of the factor is the reference level and correct
cp_proteins$condition

## [1] Treated Treated Treated Control Control Control
## Levels: Control Treated

cp_proteins$condition <- relevel(cp_proteins$condition, ref = "Control")


### Differential expression analysis using limma

Bioconductor contains several packages dedicated to the statistical analysis of proteomics data. For example, MSstats and MSstatsTMT can be used to determine differential protein expression within both DDA and DIA datasets for LFQ and TMT, respectively. ^38^^,^^39^ Of note, MSstatsTMT includes additional functionality for dealing with larger, multi-plexed TMT experiments. For LFQ experiments, proDA , prolfqua and MSqRob2 can be utilised, among others. ^32^^,^^40^ Here, we will use the limma package. ^41^ limma is widely used for the analysis of large omics datasets and has several models that allow differential abundance to be assessed in multifactorial experiments. This is useful because it allows multiple factors, including TMTplex, to be integrated into the model itself, thus minimising the effects of confounding factors. In this example we will apply limma’s empirical Bayes moderated t-test, a method that is appropriate for small sample sizes. ^31^

We first use the model.matrix function to create a matrix in which each of the samples are annotated based on the factors we wish to model, here the condition group. This ultimately defines the ‘design’ of the model, that is how the samples are distributed between the groups of interest. We then fit a linear model to the abundance data of each protein by passing the data and model design matrix to the lmFit function. Finally, we update the estimated standard error for each model coefficient using the eBayes function. This function borrows information across features, here proteins, to shift the per-protein variance estimates towards an expected value based on the variance estimates of other proteins with similar mean intensity. This empirical Bayes technique has been shown to reduce the number of false positives for proteins with small variances as well as increase the power of detection for differentially abundant proteins with larger variances. ^42^ Further, we use the trend = TRUE argument when passing the eBayes function so that an intensity-dependent trend can be fitted to the prior variances. For more information about the limma trend method users are directed to Ref. 43.

## Design a matrix containing all of the factors we wish to model the effects of
model_design <- model.matrix(~ cp_proteins$condition)

## Verify
print(model_design)

##   (Intercept) cp_proteins$conditionTreated
## 1           1                            1
## 2           1                            1
## 3           1                            1
## 4           1                            0
## 5           1                            0
## 6           1                            0
## attr(,"assign")
## [1] 0 1
## attr(,"contrasts")
## attr(,"contrasts")$‘cp_proteins$condition‘
## [1] "contr.treatment"

## Create a linear model using this design
fitted_lm <- cp_proteins %>%
  assay() %>%
  lmFit(design = model_design)

## Update the model based on Limma eBayes algorithm
fitted_lm <- eBayes(fit = fitted_lm,
                    trend = TRUE)

## Save results of the test
limma_results <- topTable(fit = fitted_lm,
                          coef = "cp_proteins$conditionTreated",
                          adjust.method = "BH",
                          number = Inf) %>%
rownames_to_column("Protein") %>%
as_tibble() %>%
mutate(TP = grepl("ups", Protein))


Having applied the model to the data, we need to verify that this model was appropriate and that the statistical assumptions were met. To do this we first generate an SA plot using the plotSA function within limma. An SA plot shows the log2 residual standard deviation (sigma) against log average abundance and is a simple way to visualise the trend that has been fitted to the data.

## Plot residual SD against average log abundance
plotSA(fitted_lm,
       xlab = "Average log2(abundance)",
       ylab = "log2(sigma)",
       cex = 0.5)

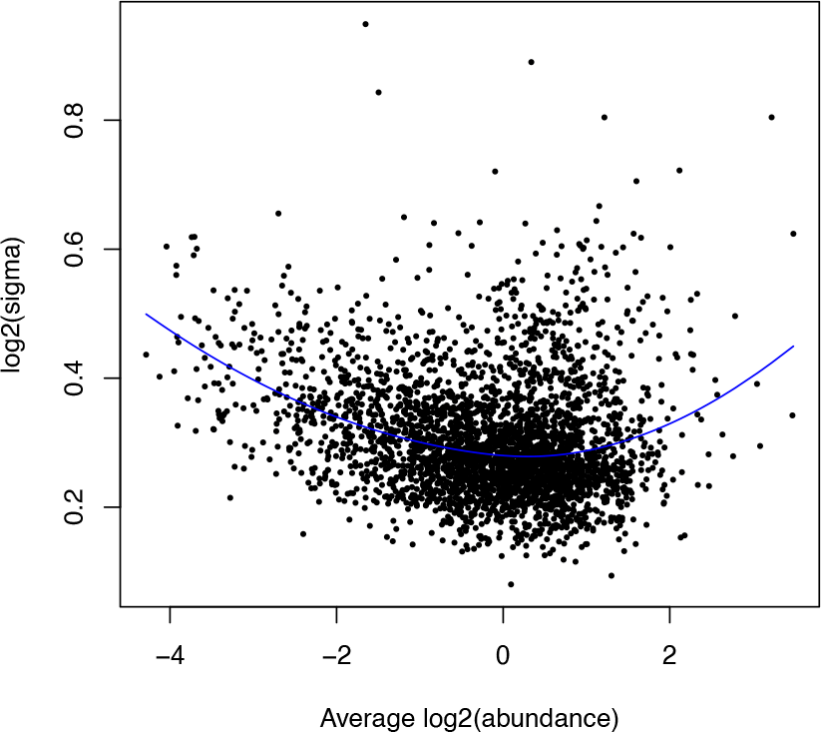


The residual standard deviation is a measure of model accuracy and is most easily conceptualised as a measurement of how far from the model prediction each data point lies. The smaller the residual standard deviation, the closer the fit between the model and observed data.

Next we will plot a p-value histogram. Importantly, this histogram shows the distribution of p-values prior to any multiple hypothesis test correction or FDR control. This means plotting the P.value variable, not the adj.P.Val.

## Plot histogram of raw p-values
limma_results %>%
  ggplot(aes(x = P.Value)) +
  geom_histogram(binwidth = 0.025) +
  labs(x = "P-value", y = "Frequency") +
  ggtitle("P-value distribution following Limma eBayes trend model") +
  theme_bw()

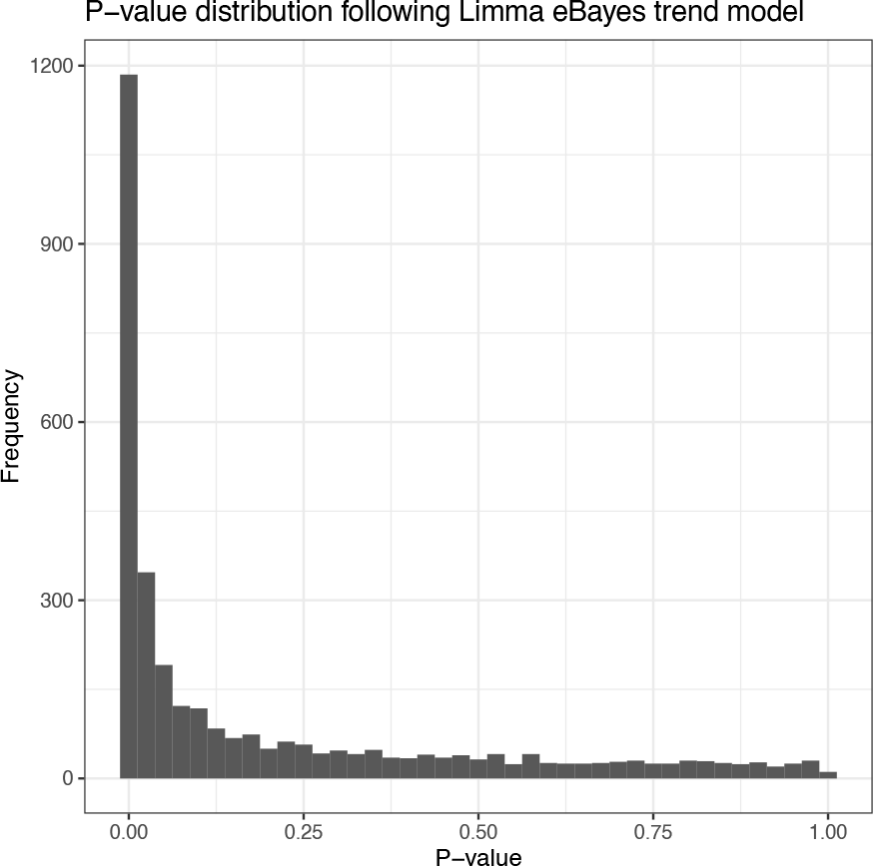


The figure displayed shows an anti-conservative p-value distribution. The flat distribution across the base of the graph represents the non-significant p-values spread uniformly between 0 and 1, whilst the peak close to 0 contains significant p-values, along with some false positives. For a more thorough explanation of interpreting p-value distributions, including why your data may not produce an anti-conservative distribution if your statistical model is inappropriate, please see Ref. 44. Now, having applied the statistical model and verified it’s suitability, we take an initial look at the outputs.

## Look at limma results table
head(limma_results)

## # A tibble: 6x8
##   Protein logFC AveExpr     t  P.Value  adj.P.Val      B TP
##   <chr>   <dbl>   <dbl> <dbl>    <dbl>      <dbl>  <dbl> <lgl>
## 1 Q9C0G0  2.97   -0.814  33.7 1.81e-10 0.000000596 14.1  FALSE
## 2 Q01581  1.50    0.588  28.4 7.87e-10 0.00000129  13.0  FALSE
## 3 P15104  1.32     1.36  23.7 3.69e- 9 0.00000404  11.7  FALSE
## 4 Q9UK41  1.46    -1.49  22.0 7.05e- 9 0.00000553  11.1  FALSE
## 5 P37268  1.32   -0.678  21.1 9.80e- 9 0.00000553  10.8  FALSE
## 6 P04183  1.29    0.939  21.1 1.01e- 8 0.00000553  10.8  FALSE


The results table contains several important pieces of information. Each master protein is represented by its accession number and has an associated log2 fold change, that is the log2 difference in mean abundance between conditions, as well as a log2 mean expression across all six samples, termed AveExpr. Since we carried out an empirical Bayes moderated t-test, each protein also has a moderated t-statistic and associated p-value. The moderated t-statistic can be interpreted in the same way as a standard t-statistic. Each protein also has an adjusted p-value which accounts for multiple hypothesis testing to control the overall FDR. The default method for multiple hypothesis corrections within the topTable function that we applied is the Benjamini and Hochberg (BH) adjustment, ^45^ although we could have specified an alternative. Finally, the B-statistic represents the log-odds that a protein is differentially abundant between the two conditions, and the data is presented in descending order with those with the highest log-odds of differential abundance at the top.

We can add annotations to this results table based on the user-defined significance thresholds. In the literature, for stringent analyses an FDR-adjusted p-value threshold of 0.01 is most frequently used, or 0.05 for exploratory analyses. Ultimately these thresholds are arbitrary and set by the user. The addition of a log-fold change ( logFC) threshold is at the users discretion and can be useful to determine significant results of biological relevance. When using a TMT labelling strategy the co-isolation interference can lead to substantial and uneven ratio compression, thus it is not recommended to apply a fold change threshold here.

## Add direction of log fold change relative to control
limma_results$direction <- ifelse(limma_results$logFC > 0,
                                  "up", "down") %>%
  as.factor()

## Add significance thresholds
limma_results$significance <- ifelse(limma_results$adj.P.Val < 0.01,
                                     "sig", "not.sig") %>%
  as.factor()

## Verify
str(limma_results)

## tibble [3,289 x 10] (S3: tbl_df/tbl/data.frame)
##  $ Protein      : chr [1:3289] "Q9C0G0" "Q01581" "P15104" "Q9UK41" …
##  $ logFC        : num [1:3289] 2.97 1.5 1.32 1.46 1.32 …
##  $ AveExpr      : num [1:3289] -0.814 0.588 1.359 -1.489 -0.678 …
##  $ t            : num [1:3289] 33.7 28.4 23.7 22 21.1 …
##  $ P.Value      : num [1:3289] 1.81e-10 7.87e-10 3.69e-09 7.05e-09 9.80e-09 …
##  $ adj.P.Val    : num [1:3289] 5.96e-07 1.29e-06 4.04e-06 5.53e-06 5.53e-06 …
##  $ B            : num [1:3289] 14.1 13 11.7 11.1 10.8 …
##  $ TP           : logi [1:3289] FALSE FALSE FALSE FALSE FALSE FALSE …
##  $ direction    : Factor w/ 2 levels "down","up": 2 2 2 2 2 2 2 1 1 2 …
##  $ significance : Factor w/ 2 levels "not.sig","sig": 2 2 2 2 2 2 2 2 2 2 …


In the next code chunk, we use the decideTests function to determine how many proteins are significantly up- and down- regulated in the treated compared to control HEK293 cells. We tell this function to classify the significance of each t-statistic based on a BH-adjusted p-value of 0.01. If we had not used TMT labels and wished to include a logFC threshold, we could have included lfc = as an argument. The function will then output a numerical matrix containing either -1, 0, or 1 for each protein in each condition, where a value of -1 indicates significant downregulation, 0 not significant and 1 significant upregulation. To simplify interpretation, we print a summary of this matrix.

## Get a summary of statistically significant results
fitted_lm %>%
  decideTests(adjust.method = "BH",
              p.value = 0.01) %>%
  summary()

##       (Intercept) cp_proteins$conditionTreated
## Down         1448                          395
## NotSig        414                         2569
## Up           1427                          325


From this table we can see that 395 proteins were downregulated in treated HEK293 cells compared to the control group whilst 325 were upregulated. Given that no logFC threshold was applied some of the significant differences in abundance may be small. Further, these results mean little without any information about which proteins these were and what roles they play within the cell. We subset the significant proteins so that we can investigate them further.

## Subset proteins that show significantly different abundance
sig_proteins <- subset(limma_results,
                       adj.P.Val <= 0.01)

length(sig_proteins$Protein)

## [1] 720


### Visualising differentially abundant proteins

Before looking deeper into which proteins have differential abundance, we first create some simple plots to visualise the results. Volcano plots and MA plots are two of the common visualisations used in this instance. When plotting the former, users are advised to plot raw p-values rather than their derivative BH-adjusted p-values. Point colours can be used to indicate significance based on BH-adjusted p-values, as is shown in the code chunk below.

## Generate a volcano plot
limma_results %>%
  ggplot(aes(x = logFC, y = -log10(P.Value))) +
  geom_point(aes(colour = significance:direction), size = 0.5) +
  scale_color_manual(
  values = c("black", "black", "deepskyblue", "red"), name = "",
  labels = c("Downregulated insignificant",
             "Upregulated insignificant",
             "Downregulated significant",
             "Upregulated significant")) +
  theme(axis.title.x = element_text(size = 15, vjust = -2),
        axis.title.y = element_text(size = 15, vjust = 2),
        axis.text.x = element_text(size = 12, vjust = -1),
        axis.text.y = element_text(size = 12),
        plot.background = element_rect(fill = "white"),
        panel.background = element_rect(fill = "white"),
        axis.line = element_line(linewidth = 0.5, colour = "black"),
        plot.margin = margin(10, 10, 10, 10),
        legend.position = c(0.25, 0.9)) +
  labs(x = "log2(FC)", y = "-log10(p-value)") +
  xlim(-3.1, 3.1)

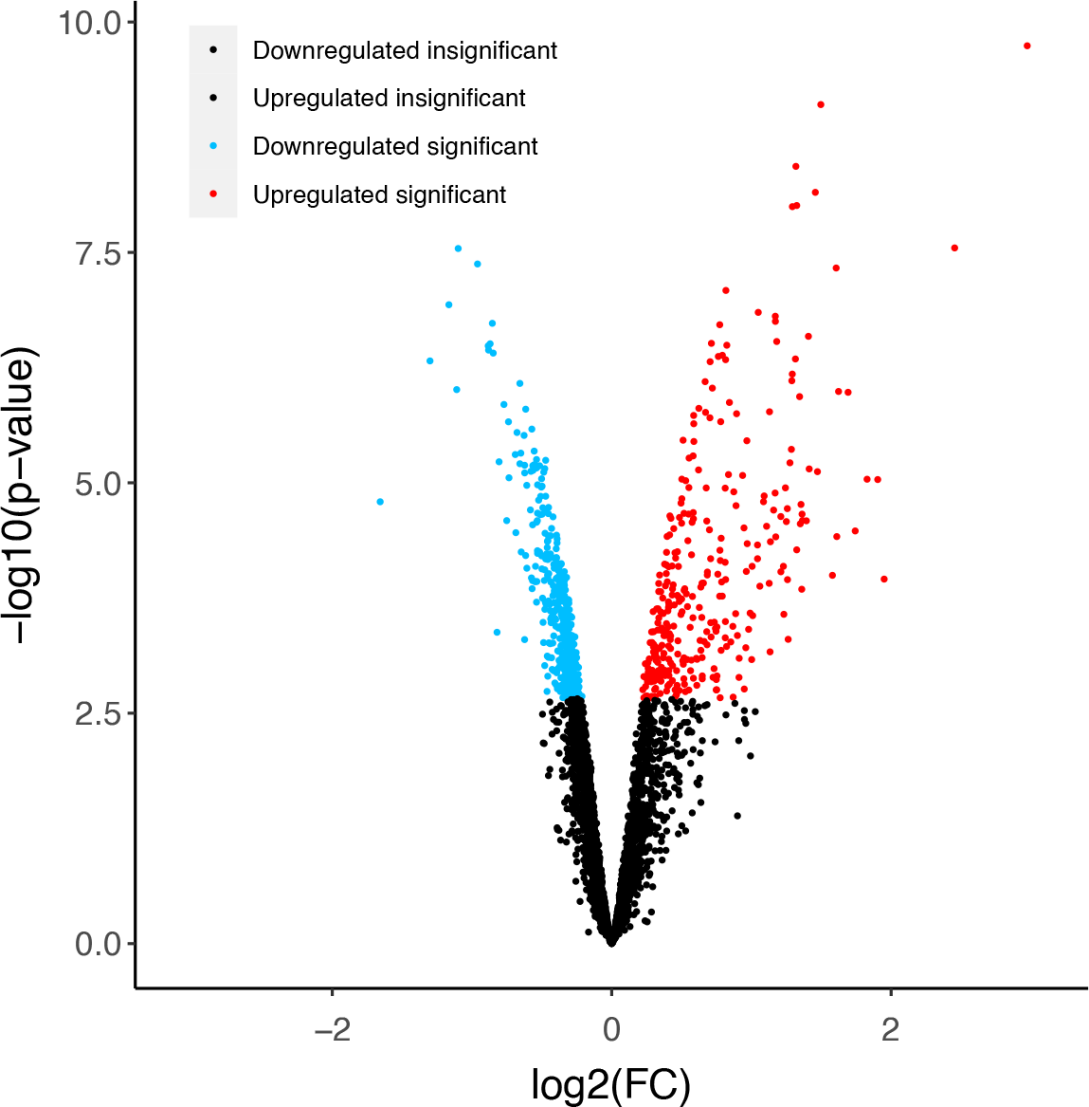


## Generate MA plot
limma_results %>%
  ggplot(aes(x = AveExpr, y = logFC)) +
  geom_point(aes(colour = significance:direction), size = 0.5) +
  scale_color_manual(
  values = c("black", "black", "deepskyblue", "red"), name = "",
  labels = c("Downregulated insignificant",
             "Upregulated insignificant",
             "Downregulated significant",
             "Upregulated significant")) +
  theme(axis.title.x = element_text(size = 15, vjust = -2),
        axis.title.y = element_text(size = 15, vjust = 2),
        axis.text.x = element_text(size = 12, vjust = -1),
        axis.text.y = element_text(size = 12),
        plot.background = element_rect(fill = "white"),
        panel.background = element_rect(fill = "white"),
        axis.line = element_line(linewidth = 0.5, colour = "black"),
        plot.margin = margin(10, 10, 10, 10),
        legend.position = c(0.25, 0.9)) +
  xlab("log2(mean abundance)") +
  ylab("log2(FC)") +
  xlim(-5, 3.5)

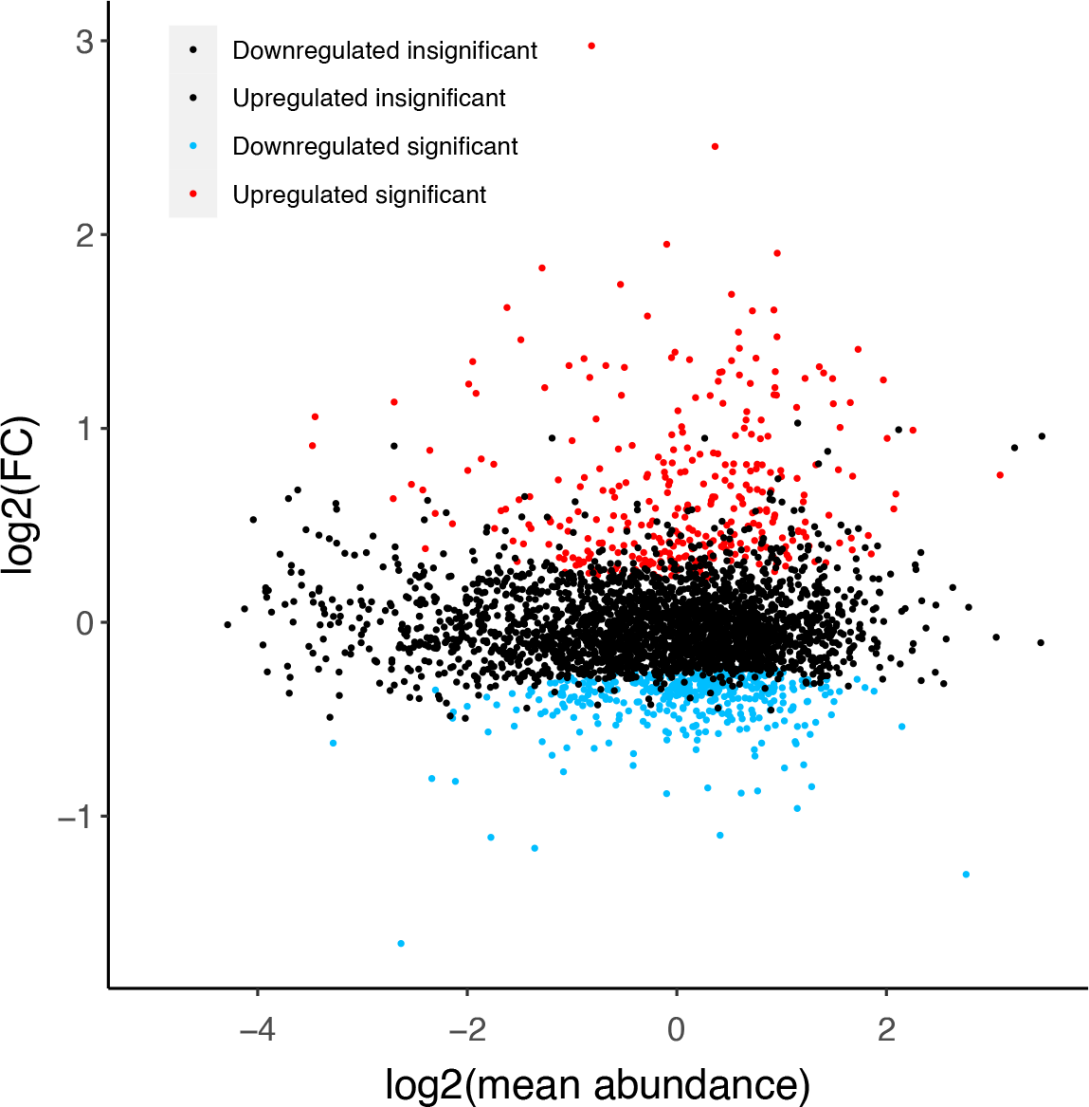


### Gene Ontology enrichment analysis

The final step in the processing workflow is to apply Gene Ontology (GO) enrichment analyses to gain a biological understanding of the proteins which were either up or downregulated in HEK293 cells upon treatment. GO terms provide descriptions for genes and their corresponding proteins in the form of Molecular Functions (MF), Biological Processes (BP) and Cellular Components (CC). By carrying out GO enrichment analysis we can determine whether the frequency of any of these terms is higher than expected in the proteins of interest compared to all of the proteins which were detected. Such results can indicate whether proteins that were increased or decreased in abundance in treated HEK293 cells represent particular cellular locations, biological pathways or cellular functions.

Although GO enrichment analysis can be carried out online using websites such as GOrilla ^46^ or PantherDB, ^47^^,^^48^ we advise against this due to a lack of traceability and reproducibility. Instead, readers are advised to make use of GO enrichment packages within the Bioconductor infrastructure. Many such packages exist, including topGO , ^49^ GOfuncR , ^50^ and clusterProfiler . ^51^ Here we will use enrichGO function in the clusterProfiler package.

First, we subset the accessions of proteins that we consider to be significantly up or downregulated. These will be our proteins of interest.

## Subset significantly upregulated and downregulated proteins
sig_up <- limma_results %>%
  filter(direction == "up") %>%
  filter(significance == "sig") %>%
  pull(Protein)

sig_down <- limma_results %>%
  filter(direction == "down") %>%
  filter(significance == "sig") %>%
  pull(Protein)


Next, we input the UniProt IDs of up and downregulated proteins into the GO enrichment analyses, as demonstrated below. Importantly, we provide the protein list of interest as the foreground and a list of all proteins identified within the study as the background, or ‘universe’. The keyType argument is used to tell the function that our protein accessions are in UniProt format. This allows mapping from UniProt ID back to a database containing the entire human genome ( org.Hs.eg.db). We also inform the function which GO categories we wish to consider, here “ALL”, meaning BP, MF and CC.

As well as the information outlined above, there is the opportunity for users to specify various thresholds for statistical significance. These include thresholds on original and adjusted p-values (using the pvalueCutoff argument) as well as q-values (via the qvalueCutoff argument). Although many papers often use ‘q- value’ to mean ‘BH-adjusted p-value’, the two are not always the same and users should be explicit about the statistical thresholds that they have applied. For exploratory purposes we will use the standard BH method for FDR control and set p-value, BH-adjusted p-value, and q-value thresholds of 0.05.

## Search for enriched GO terms within upregulated proteins
ego_up <- enrichGO(gene = sig_up,
        universe = limma_results$Protein,
        OrgDb = org.Hs.eg.db,
        keyType = "UNIPROT",
        ont = "ALL",
        pAdjustMethod = "BH",
        pvalueCutoff = 0.05,
        qvalueCutoff = 0.05,
        readable = TRUE)

## Check results
ego_up

## #
## # over-representation test
## #
## #…@organism     Homo sapiens
## #…@ontology     GOALL
## #…@keytype      UNIPROT
## #…@gene     chr [1:325] "Q9C0G0" "Q01581" "P15104" "Q9UK41" "P37268" "P04183" "Q9UHI8" …
## #…pvalues adjusted by ’BH’ with cutoff <0.05
## #…2 enriched terms found
## ’data.frame’: 2 obs. of 10 variables:
##  $ ONTOLOGY    : chr "CC" "CC"
##  $ ID          : chr "GO:0005758" "GO:0031970"
##  $ Description : chr "mitochondrial intermembrane space" "organelle envelope lumen"
##  $ GeneRatio   : chr "15/319" "15/319"
##  $ BgRatio     : chr "45/3228" "49/3228"
##  $ pvalue      : num 1.32e-05 4.18e-05
##  $ p.adjust    : num 0.00507 0.008
##  $ qvalue      : num 0.00506 0.00798
##  $ genelD      : chr "CHCHD2/TIMM9/AK2/TIMM8B/COA4/COA6/MIX23/TIMM8A/DIABLO/TIMM13/TIMM10/TRIAP1/CYCS/COX17/CAT"
##  $ Count : int 15 15
## #…Citation
##  T Wu, E Hu, S Xu, M Chen, P Guo, Z Dai, T Feng, L Zhou, W Tang, L Zhan, X Fu, S Liu, X Bo, and G Yu.
##  clusterProfiler 4.0: A universal enrichment tool for interpreting omics data.
##  The Innovation. 2021, 2(3):100141


We can see from the results that there are 2 significantly enriched terms associated with the upregulated proteins. Next, we take a look at the downregulated proteins.

## Search for enriched GO terms within downregulated proteins
ego_down <- enrichGO(gene = sig_down,
                     universe = limma_results$Protein,
                     OrgDb = org.Hs.eg.db,
                     keyType = "UNIPROT",
                     ont = "ALL",
                     pAdjustMethod = "BH",
                     pvalueCutoff = 0.05,
                     qvalueCutoff = 0.05,
                     readable = TRUE)

## Check results
ego_down

## #
## # over-representation test
## #
## #…@organism     Homo sapiens
## #…@ontology     GOALL
## #…@keytype      UNIPROT
## #…@gene     chr [1:395] "Q53EL6" "P08243" "P35716" "Q92878" "P26583" "Q92522" "O43657" …
## #…pvalues adjusted by ’BH’ with cutoff <0.05
## #…69 enriched terms found
## ’data.frame’:    69 obs. of 10 variables:
##  $ ONTOLOGY    : chr "BP" "BP" "BP" "BP" …
##  $ ID          : Chr "GO:0006310" "GO:0006520" "GO:0000725" "GO:0006302" …
##  $ Description : chr "DNA recombination" "amino acid metabolic process" "recombinational repair" "double-strand break repair" …
##  $ GeneRatio   : chr "36/378" "35/378" "22/378" "31/378" …
##  $ BgRatio     : chr "94/3166" "112/3166" "55/3166" "98/3166" …
##  $ pvalue      : num 2.27e-11 2.48e-08 8.41e-08 1.20e-07 1.01e-06 …
##  $ p.adjust    : num 5.66e-08 3.09e-05 7.00e-05 7.48e-05 5.06e-04 …
##  $ qvalue      : num 5.37e-08 2.93e-05 6.63e-05 7.09e-05 4.80e-04 …
##  $ genelD      : chr "RAD50/HMGB2/H1-10/RADX/MRE11/H1-0/H1-2/ZMYND8/HMGB3/MCM5/NUCKS1/RAD21/PRKDC/SFPQ/MCM4/XRCC6/H1-3/MCM7/TFRC/XRCC"| __truncated__ "ASNS/PHGDH/SDSL/SARS1/YARS1/AARS2/HMGCL/IARS2/GARS1/AARS1/HIBADH/PYCR1/MCCC2/ACADSB/DHFR/MARS1/SLC25A12/ETFA/PS"| __truncated__ "RADX/MRE11/ZMYND8/MCM5/NUCKS1/RAD21/SFPQ/MCM4/XRCC6/MCM7/XRCC5/PPP4R2/POGZ/YY1/MCM3/MCM2/VPS72/PARP1/BRD8/MCM6/FUS/RECQL" "RAD50/HMGB2/RADX/MRE11/DEK/ZMYND8/MCM5/NUCKS1/RAD21/PRKDC/TP53/SFPQ/SMARCC2/MCM4/XRCC6/HPF1/MCM7/XRCC5/HMGB1/PP"| __truncated__ …
##  $ Count       : int 36 35 22 31 20 56 57 18 14 56 …
## # …Citation
##  T Wu, E Hu, S Xu, M Chen, P Guo, Z Dai, T Feng, L Zhou, W Tang, L Zhan, X Fu, S Liu, X Bo, and G Yu.
##  clusterProfiler 4.0: A universal enrichment tool for interpreting omics data.
##  The Innovation. 2021, 2(3):100141


The downregulated proteins contain 69 significantly enriched GO terms. There are many ways in which users can represent these results visually. Here, we create a barplot using the barplot function from the enrichplot package. ^52^ Users are directed to the vignette of the enrichplot package for additional visualisation options and guidance. We plot the first 10 GO terms i.e. the 10 GO terms with the greatest enrichment.

## Plot the results
barplot(ego_down,
        x = "Count",
        showCategory = 10,
        font.size = 12,
        label_format = 28,
        colorBy = "p.adjust")

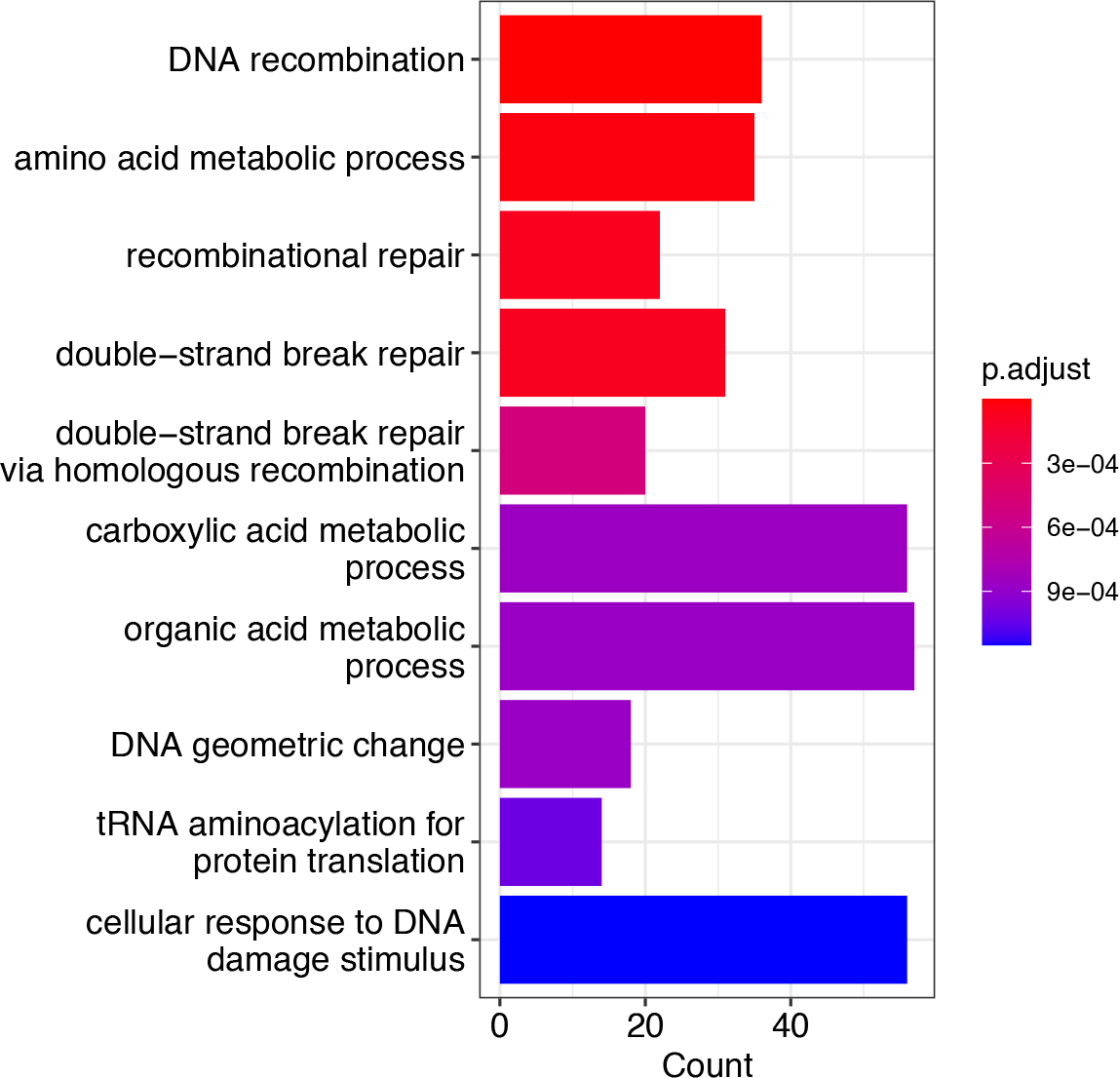


## Writing and exporting data

Finally, we export the results of our statistical analyses as .csv files.

## Save results of Limma statistics
write.csv(gene_results, file = "all_limma_results.csv")

## Save subsets of upregulated and downregulated proteins
write.csv(sig_upregulated, file = "upregulated_results.csv")
write.csv(sig_downregulated, file = "downregulated_results.csv"))

## Save results of GO enrichment
write.csv(ego_up, file = "upregulated_go_enrichment.csv")
write.csv(ego_down, file = "downregulated_go_enrichment.csv")


Users can also use the ggsave function to export any of the figures generated.

## Discussion and conclusion

Expression proteomics is becoming an increasingly important tool in modern molecular biology. As more researchers participate in expression proteomics, either by collecting data or accessing data collected by others, there is a need for clear illustration(s) of how to deal with such complex data.

Existing bottom-up proteomics workflows for differential expression analysis either provide pipelines with limited user control and flexibility (e.g., MSstats and MSstatsTMT ^38^^,^^39^), can only be applied to specific data formats (e.g., Proteus which is limited to input from MaxQuant ^53^), or provide very limited commentary. The latter directly contributes to a problematic disconnect between researchers and their data whereby the users do not understand if or why each step is necessary for their given dataset and biological question. This can prevent researchers from refining a workflow to fit their specific needs. Finally, the majority of proteomics workflows utilise data.frame or tibble structures which limits their traceability, as is the case for protti, promor and prolfqua. ^54^^–^^56^

The workflow presented here outlines in completion how to process, analyse and interpret LFQ and TMT expression proteomics data derived from a bottom-up DDA experiment. Critically, we emphasize quality control and data-guided decisions with an extensive explanation of all key steps and how they may differ in various scenarios (e.g., the quantitation method, instrumentation and biological question). Our workflow takes advantage of the relatively recent QFeatures infrastructure to ensure explicit and transparent data pre-processing as well as to provide an easy way for users to trace back through their analyses. These features are particularly important for beginners who wish to gain a better understanding of their data and how it changes throughout this workflow.

No single workflow can demonstrate the processing, analysis and interpretation of all proteomics data. Our workflow is currently suitable for DDA datasets with label-free or TMT-based quantitation. We do not include examples of experiments that combine data from multiple TMTplexes, although the code provided could easily be expanded to include such a scenario. This workflow provides an in-depth user-friendly pipeline for both new and experienced proteomics data analysts.

## Session information and getting help

The workflows provided involve use of functions from many different R/Bioconductor packages. The sessionInfo function provides an easy way to summarize all packages and corresponding their versions used to generate this document. Should software updates lead to the generation of errors or different results to those demonstrated here, such changes should be easily traced.

## Print session information
sessionInfo()

## R version 4.3.0 (2023-04-21)
## Platform: x86_64-apple-darwin20 (64-bit)
## Running under: macOS Ventura 13.4
##
## Matrix products: default
## BLAS: /Library/Frameworks/R.framework/Versions/4.3-x86_64/Resources/lib/libRblas.0.dylib
## LAPACK: /Library/Frameworks/R.framework/Versions/4.3-x86_64/Resources/lib/libRlapack.dylib; LAPACK version 3.11.0
##
## locale:
## [1] en_GB.UTF-8/en_GB.UTF-8/en_GB.UTF-8/C/en_GB.UTF-8/en_GB.UTF-8
##
## time zone: Europe/London
## tzcode source: internal
##
## attached base packages:
## [1] stats4    stats    graphics    grDevices    utils   datasets   methods
## [8] base
##
## other attached packages:
##  [1] patchwork_1.1.2             enrichplot_1.20.0
##  [3] clusterProfiler_4.8.1       org.Hs.eg.db_3.17.0
##  [5] AnnotationDbi_1.62.1        limma_3.56.2
##  [7] Biostrings_2.68.1           XVector_0.40.0
##  [9] corrplot_0.92               NormalyserDE_1.18.0
## [11] tibble_3.2.1                dplyr_1.1.2
## [13] stringr_1.5.0               ggplot2_3.4.2
## [15] QFeatures_1.10.0            MultiAssayExperiment_1.26.0
## [17] SummarizedExperiment_1.30.2 Biobase_2.60.0
## [19] GenomicRanges_1.52.0        GenomeInfoDb_1.36.0
## [21] IRanges_2.34.0              S4Vectors_0.38.1
## [23] BiocGenerics_0.46.0         MatrixGenerics_1.12.2
## [25] matrixStats_1.0.0
##
## loaded via a namespace (and not attached):
## [1] splines_4.3.0           bitops_1.0-7               ggplotify_0.1.0
## [4] cellranger_1.1.0        polyclip_1.10-4            preprocessCore_1.62.1
## [7] rpart_4.1.19            lifecycle_1.0.3            lattice_0.21-8
## [10] MASS_7.3-60            backports_1.4.1            magrittr_2.0.3
## [13] Hmisc_5.1-0            rmarkdown_2.22             yaml_2.3.7
## [16] sp_1.6-1               cowplot_1.1.1              MsCoreUtils_1.12.0
## [19] DBI_1.1.3              RColorBrewer_1.1-3         abind_1.4-5
## [22] zlibbioc_1.46.0        purrr_1.0.1                AnnotationFilter_1.24.0
## [25] ggraph_2.1.0           RCurl_1.98-1.12            yulab.utils_0.0.6
## [28] nnet_7.3-19            tweenr_2.0.2               sandwich_3.0-2
## [31] git2r_0.32.0           GenomeInfoDbData_1.2.10    ggrepel_0.9.3
## [34] tidytree_0.4.2         terra_1.7-37               nortest_1.0-4
## [37] codetools_0.2-19       DelayedArray_0.26.3        DOSE_3.26.1
## [40] ggforce_0.4.1          tidyselect_1.2.0           RcmdrMisc_2.7-2
## [43] aplot_0.1.10           raster_3.6-20              farver_2.1.1
## [46] viridis_0.6.3          base64enc_0.1-3            jsonlite_1.8.5
## [49] e1071_1.7-13           tidygraph_1.2.3            Formula_1.2-5
## [52] tools_4.3.0            treeio_1.24.1              Rcpp_1.0.10
## [55] glue_1.6.2             BiocBaseUtils_1.2.0        gridExtra_2.3
## [58] xfun_0.39              qvalue_2.32.0              usethis_2.2.0
## [61] withr_2.5.0            BiocManager_1.30.21        fastmap_1.1.1
## [64] fansi_1.0.4            digest_0.6.31              R6_2.5.1
## [67] gridGraphics_0.5-1     colorspace_2.1-0           GO.db_3.17.0
## [70] RSQLite_2.3.1          utf8_1.2.3                 tidyr_1.3.0
## [73] generics_0.1.3         data.table_1.14.8          class_7.3-22
## [76] graphlayouts_1.0.0     httr_1.4.6                 htmlwidgets_1.6.2
## [79] S4Arrays_1.0.4         scatterpie_0.2.1           pkgconfig_2.0.3
## [82] gtable_0.3.3           blob_1.2.4                 impute_1.74.1
## [85] shadowtext_0.1.2       htmltools_0.5.5            carData_3.0-5
## [88] bookdown_0.34          fgsea_1.26.0               ProtGenerics_1.32.0
## [91] clue_0.3-64            scales_1.2.1               png_0.1-8
## [94] ggfun_0.1.0            knitr_1.43                 rstudioapi_0.14
## [97] reshape2_1.4.4         nlme_3.1-162               checkmate_2.2.0
## [100] proxy_0.4-27          cachem_1.0.8               zoo_1.8-12
## [103] parallel_4.3.0        HDO.db_0.99.1              foreign_0.8-84
## [106] pillar_1.9.0          grid_4.3.0                 vctrs_0.6.3
## [109] car_3.1-2             cluster_2.1.4              htmlTable_2.4.1
## [112] evaluate_0.21         cli_3.6.1                  compiler_4.3.0
## [115] rlang_1.1.1           crayon_1.5.2               labeling_0.4.2
## [118] plyr_1.8.8            forcats_1.0.0              fs_1.6.2
## [121] stringi_1.7.12        viridisLite_0.4.2          BiocParallel_1.34.2
## [124] munsell_0.5.0         lazyeval_0.2.2             GOSemSim_2.26.0
## [127] Matrix_1.5-4.1        hms_1.1.3                  bit64_4.0.5
## [130] KEGGREST_1.40.0       haven_2.5.2                igraph_1.5.0
## [133] memoise_2.0.1         BiocWorkflowTools_1.26.0   ggtree_3.8.0
## [136] fastmatch_1.1-3       bit_4.0.5                  readxl_1.4.2
## [139] downloader_0.4        gson_0.1.0                 ape_5.7-1


Users are advised to update R itself as well as packages as required. Bioconductor packages can be updated using the BiocManager::install() function, as shown below.

if (!require("BiocManager", quietly = TRUE)) {
  install.packages("BiocManager")
}
BiocManager::install()


## Author contributions

C. H. conceptualisation, investigation, methodology, project administration, software, validation, writing – original draft preparation, review and editing; C. S. D. software and writing - review and editing; T. K. methodology, supervision, software, writing - review and editing; K. S. L. funding acquisition, supervision, writing - review and editing; L. M. B. conceptualisation, methodology, supervision, writing - review and editing.

## Data Availability

This workflow is written in the R statistical programming language and uses freely available open-source software packages from CRAN and Bioconductor. Version numbers for all packages are shown in the Session information section. Raw mass spectrometry data is freely available online through the ProteomeXchange Consortium via the PRIDE repository with identifier PXD041794. All processed data is available at http://doi.org/10.5281/zenodo.7837375, and at GitHub repository https://github.com/CambridgeCentreFor Proteomics/f1000_expression_proteomics .

## References

[1] Pina-Jiménez E Calzada F Bautista E : Incomptine a induces apoptosis, ROS production and a differential protein expression on non-hodgkin’s lymphoma cells. *Int. J. Mol. Sci.* September 2021;22(19):10516. 10.3390/ijms221910516 34638856 PMC8508949

[2] Amiri-Dashatan N Ahmadi N Rezaei-Tavirani M : Identification of differential protein expression and putative drug target in metacyclic stage of leishmania major and leishmania tropica: A quantitative proteomics and computational view. *Comp. Immunol. Microbiol. Infect. Dis.* April 2021;75:101617. 10.1016/j.cimid.2021.101617 33581562

[3] Anitua E Fuente M de la Muruzabal F : Differential profile of protein expression on human keratocytes treated with autologous serum and plasma rich in growth factors (PRGF). *PLoS One.* October 2018;13(10):e0205073. 10.1371/journal.pone.0205073 30312303 PMC6193583

[4] Dupree EJ Jayathirtha M Yorkey H : A critical review of bottom-up proteomics: The good, the bad, and the future of this field. *Proteomes.* July 2020;8(3):14. 10.3390/proteomes8030014 32640657 PMC7564415

[5] Obermaier C Griebel A Westermeier R : Principles of protein labeling techniques. *Methods in Molecular Biology.* New York: Springer;2015;153–165. 10.1007/978-1-4939-2550-6_13 25820721

[6] Fernández-Costa C Martínez-Bartolomé S McClatchy DB : Impact of the identification strategy on the reproducibility of the DDA and DIA results. *J. Proteome Res.* June 2020;19(8):3153–3161. 10.1021/acs.jproteome.0c00153 32510229 PMC7898222

[7] Alex H Noble WS Wolf-Yadlin A : Technical advances in proteomics: new developments in data-independent acquisition. *F1000Res.* March 2016;5:419. 10.12688/f1000research.7042.1 27092249 PMC4821292

[8] R Development Core Team: *R: A Language and Environment for Statistical Computing.* Vienna, Austria: R Foundation for Statistical Computing;2011. 3-900051-07-0. Reference Source

[9] Huber W Carey VJ Gentleman R : Orchestrating high-throughput genomic analysis with bioconductor. *Nat. Methods.* January 2015;12(2):115–121. 10.1038/nmeth.3252 25633503 PMC4509590

[10] Hutchings C Dawson C Krueger T : A Bioconductor workflow for processing, evaluating and interpreting expression proteomics data. 2023. Reference Source 10.12688/f1000research.139116.1 PMC10683783 38021401

[11] McAlister GC Nusinow DP Jedrychowski MP : MultiNotch MS3 enables accurate, sensitive, and multiplexed detection of differential expression across cancer cell line proteomes. *Anal. Chem.* July 2014;86(14):7150–7158. 10.1021/ac502040v 24927332 PMC4215866

[12] Ting L Rad R Gygi SP : MS3 eliminates ratio distortion in isobaric multiplexed quantitative proteomics. *Nat. Methods.* October 2011;8(11):937–940. 10.1038/nmeth.1714 21963607 PMC3205343

[13] Plubell DL Wilmarth PA Zhao Y : Extended multiplexing of tandem mass tags (TMT) labeling reveals age and high fat diet specific proteome changes in mouse epididymal adipose tissue. *Mol. Cell. Proteomics.* May 2017;16(5):873–890. 10.1074/mcp.m116.065524 28325852 PMC5417827

[14] Brenes A Hukelmann J Bensaddek D : Multibatch TMT reveals false positives, batch effects and missing values. *Mol. Cell. Proteomics.* October 2019;18(10):1967–1980. 10.1074/mcp.ra119.001472 31332098 PMC6773557

[15] Perez-Riverol Y Bai J Bandla C : The PRIDE database resources in 2022: a hub for mass spectrometry-based proteomics evidences. *Nucleic Acids Res.* November 2021;50(D1):D543–D552. 10.1093/nar/gkab1038 34723319 PMC8728295

[16] Deutsch EW Bandeira N Perez-Riverol Y : The ProteomeXchange consortium at 10 years: 2023 update. *Nucleic Acids Res.* November 2022;51(D1):D1539–D1548. 10.1093/nar/gkac1040 36370099 PMC9825490

[17] Gatto L Vanderaa C : QFeatures: Quantitative features for mass spectrometry data.R package version 1.9.2.2023. Reference Source

[18] Morgan M Obenchain V Hester J : SummarizedExperiment: SummarizedExperiment container.R package version 1.29.1.2022. Reference Source

[19] Rainer J Vicini A Salzer L : A modular and expandable ecosystem for metabolomics data annotation in r. *Metabolites.* February 2022;12(2):173. 10.3390/metabo12020173 35208247 PMC8878271

[20] Frankenfield AM Ni J Ahmed M : Protein contaminants matter: Building universal protein contaminant libraries for DDA and DIA proteomics. *J. Proteome Res.* July 2022;21(9):2104–2113. 10.1021/acs.jproteome.2c00145 35793413 PMC10040255

[21] Pages H Aboyoun P Gentleman R : Biostrings: Efficient Manipulation of Biological Strings.R package version 2.66.0.2022.

[22] Karpievitch Y Stanley J Taverner T : A statistical framework for protein quantitation in bottom-up MS-based proteomics. *Bioinformatics.* June 2009;25(16):2028–2034. 10.1093/bioinformatics/btp362 19535538 PMC2723007

[23] Lazar C Gatto L Ferro M : Accounting for the multiple natures of missing salues in label-free quantitative proteomics data sets to compare imputation strategies. *J. Proteome Res.* March 2016;15(4):1116–1125. 10.1021/acs.jproteome.5b00981 26906401

[24] Sticker A Goeminne L Martens L : Robust summarization and inference in proteome-wide label-free quantification. *Mol. Cell. Proteomics.* July 2020;19(7):1209–1219. 10.1074/mcp.ra119.001624 32321741 PMC7338080

[25] Goeminne LJE Gevaert K Clement L : Peptide-level robust ridge regression improves estimation, sensitivity, and specificity in data-dependent quantitative label-free shotgun proteomics. *Mol. Cell. Proteomics.* February 2016;15(2):657–668. 10.1074/mcp.m115.055897 26566788 PMC4739679

[26] O’Rourke MB Town SEL Dalla PV : What is normalization? the strategies employed in top-down and bottom-up proteome analysis workflows. *Proteomes.* August 2019;7(3):29. 10.3390/proteomes7030029 31443461 PMC6789750

[27] Willforss J Chawade A Levander F : NormalyzerDE: Online tool for improved normalization of omics expression data and high-sensitivity differential expression analysis. *J. Proteome Res.* October 2018;18(2):732–740. 10.1021/acs.jproteome.8b00523 30277078

[28] Bolstad B : preprocessCore: A collection of pre-processing functions.R package version 1.60.2.2023.

[29] Andersen CL Jensen JL Ørntoft TF : Normalization of real-time quantitative reverse transcription-PCR data: A model-based variance estimation approach to identify genes suited for normalization, applied to bladder and colon cancer data sets. *Cancer Res.* August 2004;64(15):5245–5250. 10.1158/0008-5472.can-04-0496 15289330

[30] Huber W Heydebreck A von Sültmann H : Variance stabilization applied to microarray data calibration and to the quantification of differential expression. *Bioinformatics.* July 2002;18(suppl_1):S96–S104. 10.1093/bioinformatics/18.suppl_1.s96 12169536

[31] Smyth GK : Linear models and empirical bayes methods for assessing differential expression in microarray experiments. *Stat. Appl. Genet. Mol. Biol.* January 2004;3(1):1–25. 10.2202/1544-6115.1027 16646809

[32] Goeminne LJE Sticker A Martens L : MSqRob takes the missing hurdle: Uniting intensity- and count-based proteomics. *Anal. Chem.* March 2020;92(9):6278–6287. 10.1021/acs.analchem.9b04375 32227882

[33] Liu M Dongre A : Proper imputation of missing values in proteomics datasets for differential expression analysis. *Brief. Bioinform.* June 2021;22(3). 10.1093/bib/bbaa112 32520347

[34] Irizarry R : Correlation is not a measure of reproducibility. 2015. Reference Source

[35] Bunting KV Steeds RP Slater LT : A practical guide to assess the reproducibility of echocardiographic measurements. *J. Am. Soc. Echocardiogr.* December 2019;32(12):1505–1515. 10.1016/j.echo.2019.08.015 31653530

[36] Darbani B Stewart CN : Reproducibility and reliability assays of the gene expression-measurements. *J. Biol. Res (Thessalon). * May 2014;21(1). 10.1186/2241-5793-21-3 25984486 PMC4376515

[37] Leek JT Scharpf RB Bravo HC : Tackling the widespread and critical impact of batch effects in high-throughput data. *Nat. Rev. Genet.* September 2010;11(10):733–739. 10.1038/nrg2825 20838408 PMC3880143

[38] Choi M Chang C-Y Clough T : MSstats: an r package for statistical analysis of quantitative mass spectrometry-based proteomic experiments. *Bioinformatics.* May 2014;30(17):2524–2526. 10.1093/bioinformatics/btu305 24794931

[39] Huang T Choi M Tzouros M : MSstatsTMT: Statistical detection of differentially abundant proteins in experiments with isobaric labeling and multiple mixtures. *Mol. Cell. Proteomics.* October 2020;19(10):1706–1723. 10.1074/mcp.ra120.002105 32680918 PMC8015007

[40] Wolski WE Nanni P Grossmann J : prolfqua: A comprehensive R-package for proteomics differential expression analysis. *J. Proteome Res.* March 2023;22(4):1092–1104. 10.1021/acs.jproteome.2c00441 36939687 PMC10088014

[41] Ritchie ME Phipson B Di W : limma powers differential expression analyses for RNA-sequencing and microarray studies. *Nucleic Acids Res.* January 2015;43(7):e47–e47. 10.1093/nar/gkv007 25605792 PMC4402510

[42] Phipson B Lee S Majewski IJ : Robust hyperparameter estimation protects against hypervariable genes and improves power to detect differential expression. *Ann. Appl. Stat.* June 2016;10(2):946–963. 10.1214/16-aoas920 28367255 PMC5373812

[43] Law CW Chen Y Shi W : voom: precision weights unlock linear model analysis tools for RNA-seq read counts. *Genome Biol.* 2014;15(2):R29. 10.1186/gb-2014-15-2-r29 24485249 PMC4053721

[44] Robinson D : How to interpret a p-value histogram. 2014. Reference Source

[45] Benjamini Y Hochberg Y : Controlling the false discovery rate: a practical and powerful approach to multiple testing. *J. R. Stat. Soc.* 1995;57(1):289–300. 10.1111/j.2517-6161.1995.tb02031.x

[46] Eden E Navon R Steinfeld I : GOrilla: a tool for discovery and visualization of enriched GO terms in ranked gene lists. *BMC Bioinformatics.* February 2009;10(1). 10.1186/1471-2105-10-48 19192299 PMC2644678

[47] Mi H Muruganujan A Thomas PD : PANTHER in 2013: modeling the evolution of gene function, and other gene attributes, in the context of phylogenetic trees. *Nucleic Acids Res.* November 2012;41(D1):D377–D386. 10.1093/nar/gks1118 23193289 PMC3531194

[48] Thomas PD Ebert D Muruganujan A : PANTHER: Making genome-scale phylogenetics accessible to all. *Protein Sci.* November 2021;31(1):8–22. 10.1002/pro.4218 34717010 PMC8740835

[49] Alexa A ": topGO: Enrichment Analysis for Gene Ontology.R package version 2.50.0.2022.

[50] Grote S : GOfuncR: Gene ontology enrichment using FUNC.R package version 1.18.0.2022.

[51] Tianzhi W Erqiang H Shuangbin X : clusterProfiler 4.0: A universal enrichment tool for interpreting omics data. *Innovation.* August 2021;2(3):100141. 10.1016/j.xinn.2021.100141 34557778 PMC8454663

[52] Yu G : enrichplot: Visualization of Functional Enrichment Result.R package version 1.18.3.2022.

[53] Gierlinski M Gastaldello F Cole C : Proteus: an R package for downstream analysis of maxquant output. *bioRxiv.* 2018. 10.1101/416511

[54] Ranathunge C Patel SS Pinky L : promor: a comprehensive R package for label-free proteomics data analysis and predictive modeling. *bioRxiv.* 2023. 10.1101/2022.08.17.503867 PMC10010602 36922981

[55] Quast J-P Schuster D Picotti P : protti: an R package for comprehensive data analysis of peptide- and protein-centric bottom-up proteomics data. *Bioinform. Adv.* December 2021;2(1). 10.1093/bioadv/vbab041 36699412 PMC9710675

[56] Wolski WE Nanni P Grossmann J : Ralph Schlapbach, and Christian Panse. prolfqua: A comprehensive r-package for proteomics differential expression analysis. *bioRxiv.* 2022. 10.1101/2022.06.07.494524 PMC10088014 36939687

