# Supplementary material for: A Bioconductor workflow for processing, evaluating, and interpreting expression proteomics data
Source: F1000Res. 2023 Oct 24;12:1402. [Version 1] doi: 10.12688/f1000research.139116.1 (PMC10683783; doi:10.12688/f1000research.139116.1)
Supplement: Peer review history

# Reviewer report: Reviewer response for version 1

**DOI:** 10.5256/f1000research.152361.r221408

**Published:** 2023-12-18

**Authors:** Amit Kumar Yadav

**Affiliations:** 1 Translational Health Science and Technology Institute (THSTI), Faridabad, Haryana, India

The article by Hutchings et al titled "A Bioconductor Workflow for Processing, Evaluating, and Interpreting Expression Proteomics Data" provides a detailed overview tutorial of the interpretation of quantitative proteomics data through R environment using TMT and LFQ workflows as examples.

**Major comments:**

1. The use of open-source tools ensures transparency and reproducibility. The open-source R software packages from the Bioconductor project also add credibility to the workflow.
2. The workflow described is comprehensive and is a good tutorial for analysis. The article describes the comprehensive workflow with R commands, covering data import, quality control, differential expression analysis, and gene ontology enrichment analysis. This approach ensures a holistic understanding of expression proteomics.
3. The described use-case examples from TMT and LFQ provides practical relevance and enhances the applicability of the workflow.
4. The target audience is for beginners who are also familiar with R. It may also be helpful to optionally provide shiny app (for GUI based application) that biologists who are unfamiliar with CLI, can also use.

**Minor Comments:**

1. This article deals with Proteome Discoverer output. Maybe inclusion of MaxQuant or other workflows may add to it reach.
2. The difference between razor and shared peptides is not clear. Perhaps rephrasing would help a newcomer to proteomics.
3. Page 40, (Under section Visualising Aggregation) “…15 peptides and 27 supporting…”. Did the author mean 27 PSMs? It is missing in the sentence. Please clarify if these were peptides or PSMs?
4. PCA is not considered fit when there are lot of missing values as in LFQ analysis. Should these be removed before PCA?
5. Authors have conflated modified and non-modified forms of the same peptides during analysis. This may not be able to detect modified changes if differential in nature.
6. Perhaps “quantitative proteomics” is a more appropriate term than “Expression proteomics”.

The article appears to be a valuable contribution to the field of quantitative proteomics, especially for R users, providing a comprehensive and user-friendly workflow.

Are the conclusions about the tool and its performance adequately supported by the findings presented in the article?

Yes

Is the rationale for developing the new software tool clearly explained?

Yes

Is the description of the software tool technically sound?

Yes

Are sufficient details of the code, methods and analysis (if applicable) provided to allow replication of the software development and its use by others?

Yes

Is sufficient information provided to allow interpretation of the expected output datasets and any results generated using the tool?

Yes

Reviewer Expertise:

quantitative proteomics, bioinformatics, computational biology, proteome informatics

I confirm that I have read this submission and believe that I have an appropriate level of expertise to confirm that it is of an acceptable scientific standard.

# Reviewer report: Reviewer response for version 1

**DOI:** 10.5256/f1000research.152361.r221410

**Published:** 2023-11-27

**Authors:** Marie Locard-Paulet

**Affiliations:** 1 Institut de Pharmacologie et de Biologie Structurale (IPBS), Toulouse, France

The authors provide the full description of a R-based workflow applied to quantitative proteomics data analysis (TMT and label-free data). It makes use of QFeature objects, a structure that is very well suited for bottom-up proteomics data analysis. I think that this is a very nice step-by-step tutorial for MS-based proteomics data analysis with R for people who start in the field and have limited coding skills. Beyond its educational aspect, it provides the backbones of a data analysis pipeline that can be modified and published alongside any manuscript that includes MS-based proteomics data analysis.

 The manuscript is very well written, and the authors provide very clear and detailed instructions on how to use the workflow. The figures are also very clear and informative. They present the data, detail what quality control to perform to assess if the data are suitable for statistical analysis. This workflow contains a lot of useful QC plots and checks that are often overlooked, including TMT labelling efficiency calculation. It makes it a good step-by-step guide on how to analyze TMT-labelled bottom-up data. I really like the description on how to double check quality metrics such as S/N and isolation interference to adapt them to the data.

 The raw data is available in PRIDE and all the fasta files and the Proteome Discoverer output files are in Zenodo. The code is in a github repository. I easily found all the data associated with this manuscript.

 Major comments:

1. It should be made clear that this script is tailored for Proteome Discoverer outputs (and maybe even for a given version of Proteome Discoverer). For most of the plotting/filtering/analysis steps, changing input would only require adapting the column names/headers, and most of the time it is very well discussed by the authors. Nevertheless, right now this script is only adapted to Proteome Discoverer outputs and this should be made clear in the abstract and the introduction, as well as the discussion.
2. Aggregation of PSM quantification values to peptides and proteins:
  Here, the authors choose to aggregate all PSM intensities corresponding to the same sequence (stripped of its modifications). I would aggregate the PSM quantities per peptidoform (sequence + localized modifications) since two ions can have the same sequence but with modifications that are differentially regulated between the conditions compared. This should be mentioned.
3. For the GO-term enrichment: only the majority protein accessions are reported in the output of the limma analysis. So there is only one gene per feature. How do you make sure that it is the most representative annotation for a given protein group? I am fully aware that this issue is ignored by the community (for lack of alternative strategy), and that most of the time people just pick one accession per group for GO-term enrichment analysis. So, I am not asking the authors to find a solution. Nevertheless, it would be nice to mention that the output of the enrichment will depend on what accession is picked per group.

Minor comments:

1. I would remove contaminants before estimation of TMT labelling efficiency.
2. I find the paragraph “Additional considerations regarding protein isoforms” unclear. The authors should rephrase sentences such as this one: “PSMs or peptides that were previously mapped to one protein and one protein group could instead be mapped to multiple proteins and one protein group.” Maybe they should use the term “canonical protein” to be more precise?
  In any cases, the issue of peptide uniqueness does not depend only on the presence of isoforms in the fasta file but also on the strategy that was chosen for protein inference. I agree with the authors that people should precisely describe what peptides/PSMs were used for quantification, and it is good to mention it. Nevertheless, this level of detail on general MS-based proteomics concept may not be necessary here. (So the entire paragraph on isoforms could maybe be removed, especially since isoforms are not mentioned later).
3. In the paragraph “Removing PSMs that are not rank 1”:
  I think that the “PSM category” that is discussed a bit later is specific of Proteome Discoverer. Some search engines report PSMs of equal score (this would correspond to the “pretty rank” in Mascot). I don’t think that all this should necessarily be discussed in this manuscript, but I insist on the fact that the workflow is tuned for Proteome Discoverer output and this should be made clear in the introduction/abstract.
4. In the paragraph “Managing missing data” for the TMT:
  the authors mention MCAR, MAR, and MNAR, but do they all apply here? I would expect TMT-labelled data to mostly have missing values due to ions being under the limit of detection because when an ion is fragmented, the reporter ions used for relative quantification are often all detected. Isn’t it the case?
  Knowing this should restrict the choice for a more suited strategy of replacement of missing values.
  Still on missing values: page 33, it is stated that “Typically, it is desirable to remove features, here PSMs, with greater than 20% missing values”. Why this number? Is this accepted by the entire community? (this comment also applies to the same step in the LFQ analysis.
  Regarding missing value replacement (discussion on LFQ, page 55): it is really nice to provide references of strategies applied to replacement of missing values. It is indeed a tricky decision to make (how to replace? Should we replace?) and there is no one-size-fits-them-all method. Why do you choose to replace at peptide level and not protein level?
5. Page 38: what do the authors mean by “report did not indicate any superior normalization method”? How would we know what normalization method works best? This point is because I am curious, not to correct any obvious mistake. It is great to try out different normalization strategies, but I don’t really see how to pick one based on the boxplots of normalized intensities (Figure 4).
6. The authors normalize the data after protein aggregation and not at the PSM level. Is this general practice? Wouldn’t it make sense to normalize before aggregation?
7. Page 44; “Data import, housekeeping and exploration”:
  the authors mention that LFQ analysis cannot be performed at PSM level but has to be done at peptide level. I think that this is dependent on the software tool that is used, and this may be specific of Proteome Discoverer. Match between run is performed at the ion level, but the intensity retrieved at this step can be reported at PSM level. I think that this is the case in MaxQuant “evidence.txt” tables, if I am not mistaken.
8. Page 63: DEqMS could be mentioned since it is specifically developed for proteomics statistical analysis. (DOI: 10.1074/mcp.TIR119.001646)
9. This is more of a naïve question regarding using Limma in this context: does it make sense to model the variance depending on intensity after missing value replacement? I know that this manuscript may not be the place to discuss this, but I would be interested to know what the authors’ opinion is on this question.
10. Additional suggestions (only suggestions that may totally be ignored/dismissed by the authors if they don’t think that they’ll improve their manuscript):
  The data is available in PRIDE (PXD041794), but the information necessary to match TMT channel/sample to conditions/replicates is only available in the associated paper. It would be great to add the Table 1 of the manuscript and a link to the zenodo repository in PRIDE alongside the data. An even better solution would be to provide the metadata in the SDRF-Proteomics format ( https://www.nature.com/articles/s41467-021-26111-3; there is a GUI to generate these files now: https://lessdrf.streamlit.app/). This would facilitate data reuse and transparency.

Small comments / typos:

- Page 3, in the code at the bottom of the page: the “,” is missing after “stringr”.
- Paragraph "Assessing the impact of non-specific data cleaning”, in the table (and other similar tables or mentions of number of protein groups and peptide stripped sequences): “proteins” should be replaced by “protein groups” since this is what is actually counted. If the authors wanted to be more precise, they could also specify that the “peptides” correspond to peptide sequences stripped of all modifications.
- Bar plot of missing value proportion page 32: what does the red dashed line correspond to?
- Page 37, when running the function `normalizer`, I got an error “No RT column specified (column named 'RT') or option not specified Skipping RT normalization.” And could not get the expected report. I am not familiar with the tool and did not investigate further.
- I don’t think that “softwares” can be used. There is no “s” at the end.
- Page 51: “cleaning is done is two steps” should be “cleaning is done in two steps”
- Page 54: in the bar plot of missing value count, what is the dashed red line?
- Page 55: “If the method requires data to display a normal distribution, users must log2 transform the data prior to imputation.” -> is there a reason to perform missing values replacement before log transformation? If not, this step could be moved to after log transform?
- Page 66: “If we had not used TMT labels and wished to include a logFC threshold, we could have included lfc = as an argument”. The characters of “ *lfc = as*” are in a different police, I think that this is a bit unclear since the “as” should be regular text. Also, maybe you could explain “have included *lfc =* followed by the minimum absolute log2-transformed fold change”.  
- Page 72: “summarize all packages and corresponding their versions used to generate” -> the sentence does not seem correct to me.

Are the conclusions about the tool and its performance adequately supported by the findings presented in the article?

Yes

Is the rationale for developing the new software tool clearly explained?

Yes

Is the description of the software tool technically sound?

Yes

Are sufficient details of the code, methods and analysis (if applicable) provided to allow replication of the software development and its use by others?

Yes

Is sufficient information provided to allow interpretation of the expected output datasets and any results generated using the tool?

Yes

Reviewer Expertise:

I work on MS-based proteomics data analysis and computational mass spectrometry.

I confirm that I have read this submission and believe that I have an appropriate level of expertise to confirm that it is of an acceptable scientific standard.

# Reviewer report: Reviewer response for version 1

**DOI:** 10.5256/f1000research.152361.r221416

**Published:** 2023-11-27

**Authors:** Diogo Borges Lima

**Affiliations:** 1 Leibniz - Forschungsinstitut für Molekulare Pharmakologie, Berlin, Germany

The article entitled "A Bioconductor workflow for processing, evaluating, and interpreting expression proteomics data" regards a full and comprehensive workflow for performing not only qualitative but also quantitative proteomics analysis.

 The manuscript is very well written and describes in detail the whole workflow for quantifying proteomics data. The authors focused on the LFQ analysis and TMT (for labeled datasets). However, this workflow relies on the results from a search engine that will provide the identified peptides. On this manuscript the authors used results from Proteome Discoverer.

 I recommended the publication, but I would like to pinpoint some minor comments:

 a) Although the authors mentioned the quantitation analyses by using TMT for labeled datasets, why they didn't show the analysis by using SILAC, once it uses XIC (the same strategy used by LFQ)?

 b) The authors used Proteome Discoverer as search engine, and the provided a template to import the results into the workflow. They could also provide other templates to turn this workflow more versatile, such as FragPipe, Patternlab for Proteomics, etc.

Are the conclusions about the tool and its performance adequately supported by the findings presented in the article?

Yes

Is the rationale for developing the new software tool clearly explained?

Yes

Is the description of the software tool technically sound?

Yes

Are sufficient details of the code, methods and analysis (if applicable) provided to allow replication of the software development and its use by others?

Yes

Is sufficient information provided to allow interpretation of the expected output datasets and any results generated using the tool?

Yes

Reviewer Expertise:

I have experience in proteomics analyses (qualitative and quantitative); XL-MS analysis. I also develop software for identifying and quantifying proteomics datasets.

I confirm that I have read this submission and believe that I have an appropriate level of expertise to confirm that it is of an acceptable scientific standard.
